# Applying an ecosystem services framework on nature and mental health to recreational blue space visits across 18 countries

**DOI:** 10.1038/s41598-023-28544-w

**Published:** 2023-03-06

**Authors:** Joanne K. Garrett, Mathew P. White, Lewis R. Elliott, James Grellier, Simon Bell, Gregory N. Bratman, Theo Economou, Mireia Gascon, Mare Lõhmus, Mark Nieuwenhuijsen, Ann Ojala, Anne Roiko, Matilda van den Bosch, Catharine Ward Thompson, Lora E. Fleming

**Affiliations:** 1 European Centre for Environment and Human Health, Knowledge Spa, Royal Cornwall Hospital, University of Exeter Medical School, Truro, Cornwall TR1 3HD UK; 2 Cognitive Science HUB, University of Vienna, Vienna, Austria; 3 Institute of Psychology, Jagiellonian University, ul. Ingardena 6, 30-060 Krakow, Poland; 4 Estonian University of Life Sciences, Tartu, Estonia; 5 OPENspace Research Centre, Edinburgh School of Architecture and Landscape Architecture, University of Edinburgh, Edinburgh, EH3 9DF UK; 6 School of Environmental and Forest Sciences, University of Washington, Seattle, USA; 7 College of Engineering, Mathematics, and Physical Sciences, University of Exeter, Exeter, UK; 8 Climate and Atmosphere Research Centre, The Cyprus Institute, Aglandjia, Cyprus; 9 ISGlobal, Barcelona, Spain; 10 Universitat Pompeu Fabra (UPF), Barcelona, Spain; 11 CIBER Epidemiología y Salud Pública (CIBERESP), Madrid, Spain; 12 Institute of Environmental Medicine, Karolinska Institute, Solna, Sweden; 13 Natural Resources Institute Finland (Luke), Helsinki, Finland; 14 Cities Research Institute and Menzies Health Institute Queensland, Griffith University, Gold Coast, Australia; 15 School of Population and Public Health, University of British Columbia, Vancouver, Canada; 16 Department of Forest and Conservation Sciences, University of British Columbia, Vancouver, Canada

**Keywords:** Ecosystem services, Psychology

## Abstract

The effects of ‘nature’ on mental health and subjective well-being have yet to be consistently integrated into ecosystem service models and frameworks. To address this gap, we used data on subjective mental well-being from an 18-country survey to test a conceptual model integrating mental health with ecosystem services, initially proposed by Bratman et al. We analysed a range of individual and contextual factors in the context of 14,998 recreational visits to blue spaces, outdoor environments which prominently feature water. Consistent with the conceptual model, subjective mental well-being outcomes were dependent upon on a complex interplay of environmental type and quality, visit characteristics, and individual factors. These results have implications for public health and environmental management, as they may help identify the bluespace locations, environmental features, and key activities, that are most likely to impact well-being, but also potentially affect recreational demand on fragile aquatic ecosystems.

## Introduction

Ecosystem services (ESS) are the contributions to individuals and society that result from the natural environment and from healthy ecosystem processes. Viewing relationships between society and the natural world through the lens of ESS has become a major scientific and policy paradigm^1^. Large, international, and interdisciplinary teams have characterised both direct and indirect goods and services (i.e. provisioning services such as fish to eat and timber to build with; and regulating services, such as climate mitigation), and the services and processes that underpin these (i.e. supporting services such as nutrient cycling)^1,2^. Such work developing analysis and understanding of ESS may contribute to improved management and conservation of the natural world and towards raising our awareness of the dangers society face if we continue to mismanage natural resources. However, considerable research and policy gaps remain^1^.

Ultimately, the fundamental constituents of human well-being extend beyond the provision of necessities that fulfil basic needs. Clean air and water, sufficient food, and materials for shelter are critical for survival, but more extensive resources are required in addition to satisfy all the constituents of well-being. For example, the Millennium Ecosystem Assessment (MA), an assessment of the human health and well-being impacts of changes in biodiversity and ecosystems across the world, draws on Maslow’s hierarchy of human needs^3^ in recognizing the importance of ‘basic materials for a good life’ as well as identifying other key ‘constituents of well-being’. This include ‘feelings of personal safety’, ‘feeling well’, ‘good social relationships’ and ‘opportunities to be able to achieve what an individual values doing and being’^2^. Fundamentally, the MA recognises that human well-being is multidimensional, including aspects of social, mental, and physical health, in alignment with the definition of health of the World Health Organisation (WHO).

To date, applications of the ESS framework have primarily focused on the management of goods and services that satisfy our basic needs for survival and, to some extent, regulating services, such as urban greening actions for heat regulation^4^. Less attention has been given to mental health and well-being and the conditions necessary for humans to flourish^5,6^. Comparatively few studies analyse “cultural ecosystem services”, consisting of the non-material benefits people derive from nature, such as spiritual, educational, and recreational resources^7,8^. Until recently, an over-arching framework for bringing together an ESS perspective with these perspectives on mental health and well-being has been lacking.

To address this gap, Bratman and colleagues^9^ proposed a four-step conceptual model as the basis for integrating mental health and well-being into the ESS framework. The four steps for assessment of “psychological ecosystem services” were proposed as characterisation of: natural features, exposure, experience, and effects. Although a hypothetical example of how the conceptual model might be used in practice was provided (i.e. how the presence of street trees might be related to stress and antidepressant prescriptions), the authors called for detailed empirical research that worked through all the proposed steps using appropriate data that allowed consideration of different contexts and outcomes^9^.

Our aim was to operationalise, and consider the utility of, this model by providing an empirical example. We focused on people’s most recent recreational visits to ‘bluespace’ environments as a case study of an ecosystem-mental health interaction. Blue spaces are defined as outdoor environments, either natural or manmade, that prominently feature water and are accessible to people^10^. Few international datasets exist that contain sufficient information on recreational visits to allow full application of the model. One such dataset is the BlueHealth International Survey (BIS^11^), which resulted from an 18-country survey focused on bluespace environments, collected as part of the EU Horizon 2020-funded BlueHealth project (https://bluehealth2020.eu), which was specifically focused on the links between blue spaces and human health and well-being^10,11^.

**Figure 1:**
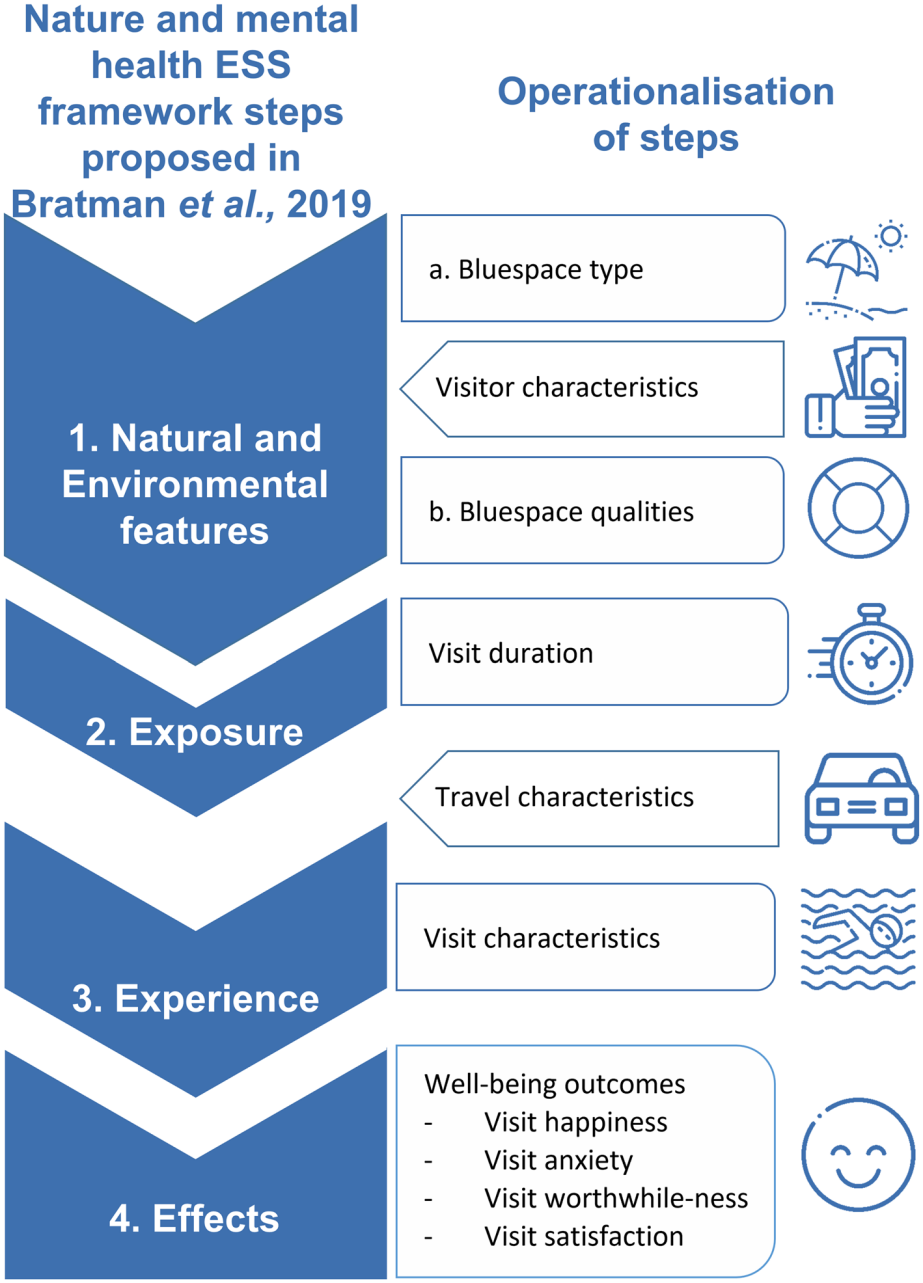
Operationalisation of the conceptual model. Conceptual model showing the ecosystem service framework proposed in Bratman et al.^9^; and our operationalisation of these steps and covariates (visitor and travel characteristics). (Icons from Kiranshastry, freepik, smashicons, srip & xnimrodx at flaticon.com).

Although relatively under-researched, evidence suggests that blue spaces may be particularly good at promoting mental health^12^. Figure 1 presents an overview of our operationalisation of the Bratman et al.^9^ conceptual model to specific bluespace visits. We applied this approach using data from nearly 15,000 (out of nearly 19,000 in total) respondents to the BIS who had reported visiting a bluespace in the four weeks prior to the survey and described their most recent visit.

At Step 1, we operationalise ‘natural features’ in terms of bluespace type, e.g. lakes or beaches, and quality, which we call here ‘*Natural and Environmental features*’, to incorporate both natural and anthropogenic aspects of the location. We also include ‘Visitor characteristics’ at this stage, as certain individual- or population-level characteristics may be correlated with visiting certain types of bluespace. For example, in England, females were found to be more likely to visit coastal environments compared to males and those living in the north east of the country were most likely to visit compared to other regions^13^. The ‘Bluespace qualities’ examined included water quality, presence of wildlife, personal safety, presence of litter/vandalism and facilities, which have been found to be important for visit-related well-being outcomes in previous literature^14,15^.

Step 2 of the Bratman et al.^9^ original conceptual model notes that ‘*Exposure*’ metrics typically focus on proximity of the particular natural environment space to reflect day-to-day exposure, and points to the limitations of estimating exposure by geography. Given that the current application of the model focused on specific recreational visits, and in line with the original authors’ call to integrate other metrics, our ‘*Exposure*’ metric here was operationalised as *‘*Visit duration’^14,16,17^. Visit duration may be influenced by travel characteristics (e.g. time taken to travel to the setting) which we therefore include at this step.

For Step 3, ‘*Experience*’, we focused on two facets of visit experience: the reported primary activity engaged in during the visit (e.g. walking, swimming); and visit companions in terms of other adults and children. Activity type and companions have both been found to be associated with well-being outcomes of visits to nature^14,17^, with the opportunity to spend time with friends and family being an important motive for visiting blue spaces in particular^13,18^.

For Step 4, *‘Effects’*, our mental health outcome was *subjective mental well-being.* This is a multi-faceted concept that focuses on individuals’ own appraisals of their everyday lives^19^. Specifically, the BIS adapted the headline items (happiness yesterday; anxiety yesterday; worthwhile-ness of activities and pursuits; and life satisfaction) used in international surveys by the OECD^20^ to evaluate a visit experience. These measure the following four facets respectively: positive emotions, negative emotions (together constituting “hedonic well-being”), “eudaimonic well-being”, and “evaluative well-being” (see White et al.^21^ for evidence that different types of nature exposure are associated with these different facets in different ways).

Following the conceptual model’s structure, the three main research questions (RQ) for our application were:

- *RQ1) Which ‘natural and environmental features’, in terms of bluespace type (1a) and perceived qualities (1b), are associated with the highest levels of recalled visit mental well-being?;*
- *RQ2) How is exposure, as operationalised by duration of visit, related to recalled visit mental well-being?; and*
- *RQ3) What experiences in blue spaces, in terms of activities (3a) and companions (3b), are associated with the most positive recalled visit mental well-being outcomes?*

Different components of the conceptual model may interact in complex ways, e.g. the interplay between the various socio-demographic factors with environmental qualities. Detailed exploration of all potential interactions of this kind is not possible in a single paper. Nevertheless, we explored one interaction in more detail, as an example of the types of analyses that can be conducted. Given the importance of species diversity in ESS frameworks and the relatively small literature on relationships between biodiversity and promotion of mental health^22,23^, we investigated whether the relationship between the presence of wildlife, as a contextual proxy for biodiversity, and visit satisfaction varied for different bluespace settings. This resulted in a fourth research question:

- *RQ4) Does the relationship between wildlife presence and recalled visit well-being vary by bluespace setting?*

In short, the present research used data from a large international survey of recent recreational visits to blue spaces to consider the utility of applying the Bratman et al.^9^ conceptual model, incorporating mental health into the ESS framework, to analyse relationships between different aspects of nature visits and mental well-being outcomes.

## Results

### Descriptive statistics

A representative sample (by age, sex and, in most countries, region of residence [see “Materials and method” section]) of approximately 1000 respondents in each of 18 different countries/regions (14 European, as well as Queensland [Australia], Canada, California [USA], and Hong Kong [China]) self-completed an online survey over four seasonal waves across 2017–2018. Full details of the survey are available online^11^. Of the full sample (n = 18,838), 14,998 individuals reported at least one recreational visit to a bluespace in the four weeks prior to the survey and were asked more details about their most recent visit. Due to missing response values, the samples used for subsequent modelling were slightly lower (n = 14,891 for models investigating anxiety and worthwhile-ness; and n = 14,892 for models investigating happiness and satisfaction; see “Materials and method” section). Details of all the variables and their categorisations are explained in the “Materials and method” section and summarised in Table S1.

### Natural and environmental features

#### Bluespace type

From a list of 17, the most visited bluespace type was a seaside promenade (17.0%), followed by a natural or artificial lake (13.0%) and urban river/canal (12.8%). The bluespace type visited least often was a salt marsh, estuary, or lagoon (0.5%, see Table S2 for all locations).

#### Bluespace qualities

Respondents were asked to state their level of agreement with four statements about their perceptions of the environmental qualities: “I felt safe”, “the area was free from litter/vandalism”, “there were good facilities (e.g. parking, footpaths, toilets)” and “there was wildlife to see and enjoy”. Response options ranged from strongly disagree (− 3) to strongly agree (+ 3). The most common responses were “agree” (+ 2) for each of the four qualities (safety = 35.0%; litter = 29.4%; facilities = 24.2%; wildlife = 22.4%; Table S2). Respondents were also asked to rate the quality of the water with response options of “poor”, “sufficient”, “good” or “excellent”, corresponding to the EU’s bathing water quality designations^10,24^. The most frequent perceived water quality rating was “Good” (46.6%; Table S2).

#### Exposure

Respondents were asked how long they spent at the bluespace (in 10 min intervals, up to four hours). These were categorised into six time periods: 0 to < 30 min (19.2% of all visits); 30 to < 60 min (20.2%); 60 to < 90 min (20.1%); 90 to < 120 min (7.2%); 120 to 180 min (16.5%); and ≥ 180 min (16.2%; Table S2).

#### Experiences

The most common activity (chosen from a list of 31 activities, re-categorised to 18 for current purposes; Tables S1,S2) was “Walking without a dog” (30.3%) followed by “Walking with a dog” (10.5%). The least common activities were non-water related sports (n = 156, 1.0%), and conservation (e.g. litter-picking; n = 42, 0.3%). Respondents were asked how many adults and children accompanied them on their visit. This was categorised to visiting alone (35.5%), visiting with other adults only (37.4%), with both adults and children (21.0%) and with children only (6.2%; Table S2).

#### Effects

Study participants were asked the extent to which the visit made them feel: (a) happy (positive emotions); (b) anxious (negative emotions); (c) how worthwhile they found the visit (eudaimonic well-being); and (d) how satisfied they were with the visit (evaluative well-being). These subjective mental well-being outcomes were measured on seven-point Likert scales from strongly disagree (− 3) to strongly agree (+ 3). Mean values for happiness, worthwhile-ness and satisfaction ranged from +1.81 to +1.91, and was − 1.85 for anxiety (indicating a disagreement with the statement that the visit made them feel anxious) (Table S3).

Descriptive data for visitor characteristics, general mental well-being (to control for people with different levels of well-being visiting different types of bluespace), and travel-related features of the trip, which were all used as covariates in the main analyses, are presented in Tables S2,S3.

### Exploration of the research questions

Our multi-step linear modelling process is presented in Fig. 1. Models were fitted for each well-being outcome separately. We present the fully adjusted results in this section (step 3 with all covariates, Fig. 1). The models explain a relatively high proportion of the variability in the outcome variable, with pseudo − *R*^2^ values ranging from 0.22 (visit anxiety) to 0.39 (visit satisfaction) (Tables S4–S7).

#### Research question 1—natural and environmental features & well-being

##### 1a) Bluespace type

**Figure 2:**
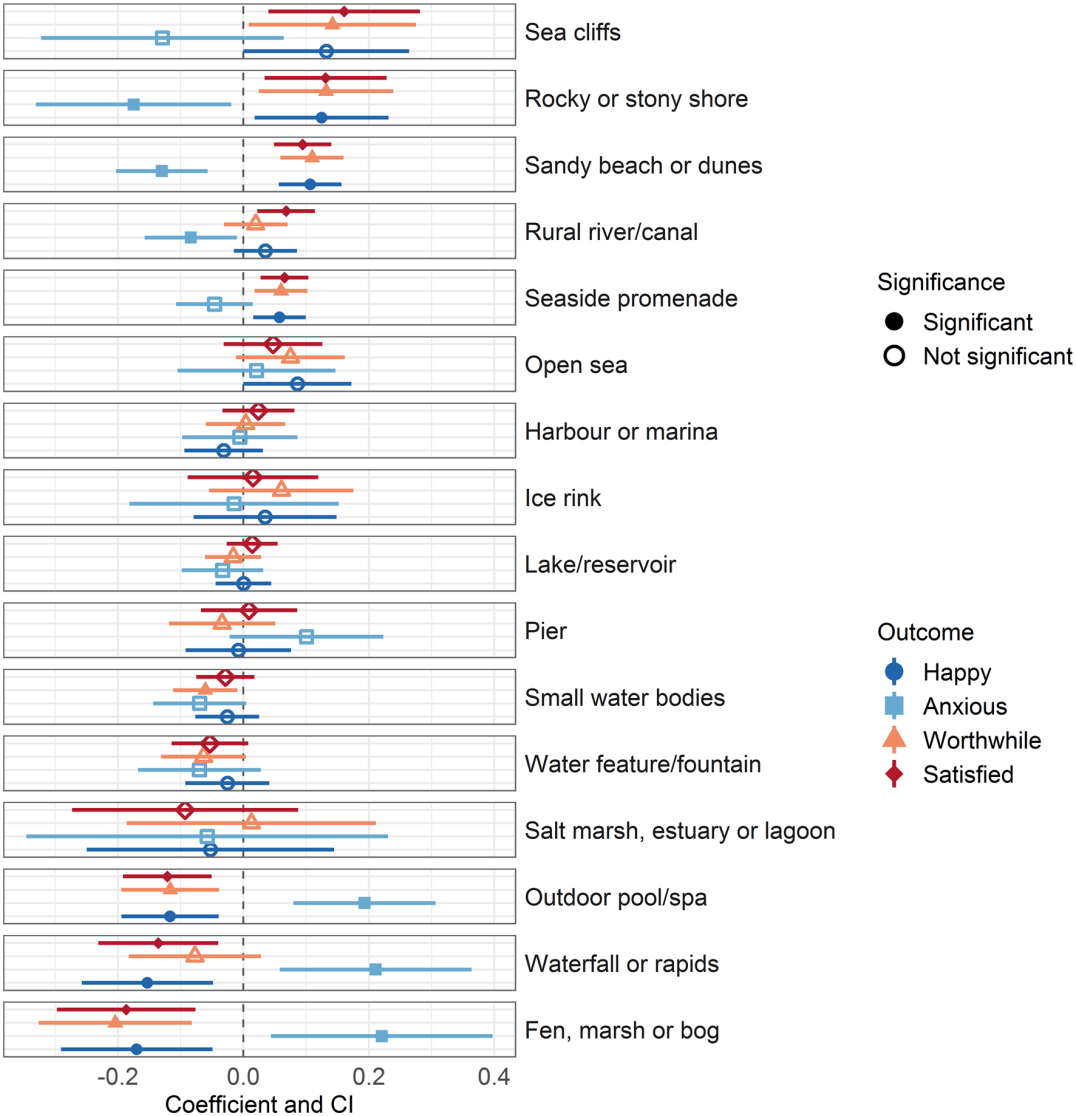
Well-being outcomes associated with bluespace type. Fully adjusted linear model (Step 3) coefficients and 95% confidence intervals, associated with each bluespace type and well-being outcome in comparison to the overall mean. Significance (at *p* < 0.05) can be observed where the 95% CI does not cross 0 (displayed with a dashed horizontal line). Note that negative coefficients for anxiety are associated with better well-being.

Bluespace types were modelled with sum contrasts. As such, coefficients are in comparison to the grand mean, which is the mean of the mean well-being outcome for each bluespace type adjusted for the other variables and covariates. For example, visiting a seaside promenade was associated with a 0.06 (95% CI = 0.02, 0.10) higher happiness value compared to the grand mean while visiting an outdoor pool was associated with − 0.12 (95% CI = − 0.19, − 0.04) happiness value compared to the grand mean (Tables 1,S4). Descriptions of significant results below indicate where *p* < 0.05 and the 95% confidence interval does not cross zero. Well-being responses varied by bluespace type, in both unadjusted and adjusted models (Table 1, Tables S4–S7). Figure 2 presents the fully adjusted coefficients [and 95% confidence intervals (CI)], corresponding with Step 3, associated with each bluespace type for each well-being outcome, ordered by their modelled effect size in association with visit satisfaction.

**Table 1:** Well-being by environmental features, exposure and experience.

Names	Happy	Anxious	Worthwhile	Satisfied
Coefficient [95% confidence interval]				
Intercept	0.55 *** [0.44, 0.67]	− 1.50 *** [− **1.68, **− **1.31]**	0.64 *** [0.53, 0.76]	0.74 *** [0.64, 0.84]
RQ1 Natural and environmental features				
(1a) Bluespace type				
Seaside promenade	0.06 ** [0.02, 0.10]	− 0.05 [− 0.11, 0.01]	0.06 ** [0.02, 0.10]	0.07 *** [0.03, 0.10]
Lake/reservoir	0.00 [− 0.04, 0.04]	− 0.03 [− 0.10, 0.03]	− 0.02 [− 0.06, 0.03]	0.01 [− 0.03, 0.05]
Sandy beach or dunes	0.11 *** [0.06, 0.16]	− 0.13 *** [− **0.20, **− **0.06]**	0.11 *** [0.06, 0.16]	0.09 *** [0.05, 0.14]
Small water bodies	− 0.03 [− 0.08, 0.03]	− 0.07 [− 0.14, 0.00]	− 0.06 * [− **0.11, **− **0.01]**	− 0.03 [− 0.08, 0.02]
Rural river/canal	0.04 [− 0.02, 0.09]	− 0.08 * [− **0.16, **− **0.01]**	0.02 [− 0.03, 0.07]	0.07 ** [0.02, 0.11]
Harbour or marina	− 0.03 [− 0.09, 0.03]	− 0.01 [− 0.10, 0.09]	0.00 [− 0.06, 0.07]	0.02 [− 0.03, 0.08]
Water feature/fountain	− 0.03 [− 0.09, 0.04]	− 0.07 [− 0.17, 0.03]	− 0.06 [− 0.13, 0.00]	− 0.05 [− 0.11, 0.01]
Outdoor pool/spa	− 0.12 ** [− **0.19, **− **0.04]**	0.19 *** [0.08, 0.31]	− 0.12 ** [− **0.19, **− **0.04]**	− 0.12 *** [− **0.19, **− **0.05]**
Pier	− 0.01 [− 0.09, 0.08]	0.10 [− 0.02, 0.22]	− 0.03 [− 0.12, 0.05]	0.01 [− 0.07, 0.09]
Open sea	0.09 [− 0.00, 0.17]	0.02 [− 0.11, 0.15]	0.07 [− 0.01, 0.16]	0.05 [− 0.03, 0.13]
Waterfall or rapids	− 0.15 ** [− **0.26, **− **0.05]**	0.21 ** [0.06, 0.36]	− 0.08 [− 0.18, 0.03]	− 0.14 ** [− **0.23, **− **0.04]**
Ice rink	0.03 [− 0.08, 0.15]	− 0.01 [− 0.18, 0.15]	0.06 [− 0.05, 0.18]	0.02 [− 0.09, 0.12]
Rocky or stony shore	0.12 * [0.02, 0.23]	− 0.18 * [− **0.33, **− **0.02]**	0.13 * [0.02, 0.24]	0.13 ** [0.03, 0.23]
Fen, marsh or bog	− 0.17 ** [− **0.29, **− **0.05]**	0.22 * [0.04, 0.40]	− 0.20 ** [− **0.33, **− **0.08]**	− 0.19 *** [− **0.30, **− **0.08]**
Sea cliffs	0.13 [− 0.00, 0.26]	− 0.13 [− 0.32, 0.06]	0.14 * [0.01, 0.28]	0.16 ** [0.04, 0.28]
Salt marsh, estuary or lagoon	− 0.05 [− 0.25, 0.14]	− 0.06 [− 0.35, 0.23]	0.01 [− 0.19, 0.21]	− 0.09 [− 0.27, 0.09]
Urban river/canal	–	–	–	–
(1b) Perceived bluespace qualities				
Safety	0.28 *** [0.27, 0.30]	− 0.20 *** [− **0.22, **− **0.18]**	0.27 *** [0.26, 0.29]	0.27 *** [0.26, 0.29]
Presence of wildlife	0.07 *** [0.06, 0.08]	0.06 *** [0.05, 0.07]	0.06 *** [0.05, 0.07]	0.05 *** [0.04, 0.05]
Absence of litter/vandalism	0.04 *** [0.03, 0.05]	− 0.01 [− 0.03, 0.00]	0.04 *** [0.03, 0.05]	0.05 *** [0.04, 0.06]
Presence of good facilities	0.03 *** [0.01, 0.04]	0.03 *** [0.02, 0.05]	0.03 *** [0.02, 0.04]	0.03 *** [0.02, 0.04]
Water quality				
Excellent	0.20 *** [0.15, 0.25]	− 0.11 ** [− **0.18, **− **0.04]**	0.16 *** [0.11, 0.20]	0.20 *** [0.15, 0.24]
Good	0.05 * [0.01, 0.09]	− 0.04 [− 0.09, 0.02]	0.02 [− 0.02, 0.05]	0.03 [− 0.00, 0.07]
Sufficient (ref)				
Poor	− 0.01 [− 0.07, 0.06]	− 0.02 [− 0.11, 0.07]	− 0.02 [− 0.09, 0.04]	− 0.10 *** [− **0.15, **− **0.04]**
RQ2. Exposure				
Visit duration				
> = 3 h	0.45 *** [0.39, 0.51]	− 0.23 *** [− **0.32, **− **0.15]**	0.51 *** [0.45, 0.57]	0.45 *** [0.39, 0.50]
2–< 3 h	0.39 *** [0.33, 0.44]	− 0.14 *** [− **0.22, **− **0.06]**	0.43 *** [0.38, 0.49]	0.36 *** [0.31, 0.41]
1.5–< 2 h	0.37 *** [0.31, 0.43]	− 0.16 *** [− **0.25, **− **0.06]**	0.40 *** [0.34, 0.47]	0.31 *** [0.26, 0.37]
1–< 1.5 h	0.31 *** [0.26, 0.36]	− 0.07 [− 0.14, 0.00]	0.33 *** [0.28, 0.38]	0.28 *** [0.24, 0.33]
30 min–< 1 h	0.21 *** [0.16, 0.26]	0.02 [− 0.05, 0.09]	0.24 *** [0.19, 0.29]	0.20 *** [0.15, 0.24]
< 30 min (ref)				
RQ3. Experience				
Activity				
Walking without a dog	**–**	–	**–**	–
Walking with a dog	0.10 *** [0.05, 0.16]	− 0.05 [− 0.12, 0.03]	0.10 *** [0.04, 0.15]	0.09 *** [0.05, 0.14]
Socialising	0.12 *** [0.07, 0.18]	− 0.13 ** [− **0.22, **− **0.05]**	0.06 * [0.01, 0.12]	0.08 ** [0.03, 0.14]
Appreciating nature	0.06 [− 0.00, 0.12]	− 0.20 *** [− **0.29, **− **0.11]**	− 0.03 [− 0.09, 0.04]	0.02 [− 0.04, 0.08]
Swimming	0.04 [− 0.02, 0.11]	− 0.08 [− 0.18, 0.01]	0.06 [− 0.01, 0.12]	0.04 [− 0.02, 0.10]
Other	− 0.13 *** [− **0.19, **− **0.07]**	0.04 [− 0.05, 0.13]	− 0.14 *** [− **0.20, **− **0.08]**	− 0.11 *** [− **0.17, **− **0.06]**
Playing with children	0.24 *** [0.17, 0.31]	− 0.21 *** [− **0.31, **− **0.11]**	0.19 *** [0.12, 0.26]	0.19 *** [0.13, 0.25]
Running/nordic walking	− 0.08 * [− **0.15, **− **0.01]**	0.30 *** [0.20, 0.40]	− 0.07 [− 0.14, 0.00]	− 0.11 ** [− **0.17, **− **0.04]**
Sunbathing/paddling	0.12 ** [0.04, 0.19]	− 0.21 *** [− **0.31, **− **0.10]**	0.03 [− 0.04, 0.10]	0.04 [− 0.03, 0.10]
Eating or drinking	0.04 [− 0.03, 0.11]	− 0.18 ** [− **0.28, **− **0.07]**	− 0.02 [− 0.10, 0.05]	0.03 [− 0.04, 0.09]
Quiet activities	0.09 * [0.01, 0.17]	− 0.27 *** [− **0.39, **− **0.16]**	0.06 [− 0.02, 0.14]	0.06 [− 0.01, 0.13]
Winter activities	− 0.12 ** [− **0.21, **− **0.03]**	0.28 *** [0.15, 0.41]	− 0.12 ** [− **0.21, **− **0.04]**	− 0.04 [− 0.11, 0.04]
Cycling	− 0.02 [− 0.10, 0.06]	0.03 [− 0.09, 0.15]	− 0.05 [− 0.14, 0.03]	− 0.06 [− 0.14, 0.01]
Watersports/boating	− 0.01 [− 0.09, 0.08]	0.05 [− 0.08, 0.17]	0.04 [− 0.05, 0.13]	0.05 [− 0.03, 0.13]
Visiting an attraction	0.04 [− 0.05, 0.14]	− 0.10 [− 0.24, 0.04]	0.01 [− 0.09, 0.10]	0.05 [− 0.04, 0.13]
Fishing	0.11 [− 0.00, 0.23]	− 0.00 [− 0.17, 0.16]	− 0.06 [− 0.17, 0.06]	− 0.05 [− 0.15, 0.06]
Sport	− 0.22 ** [− **0.35, **− **0.08]**	0.30 ** [0.11, 0.50]	− 0.13 [− 0.26, 0.01]	− 0.22 *** [− **0.34, **− **0.10]**
Conservation	− 0.41 ** [− **0.66, **− **0.16]**	0.63 *** [0.26, 1.00]	0.07 [− 0.18, 0.33]	− 0.10 [− 0.33, 0.13]
Visit companions				
Adults and children	− 0.05 * [− **0.10, **− **0.01]**	0.23 *** [0.16, 0.29]	− 0.03 [− 0.08, 0.01]	− 0.05 * [− **0.09, **− **0.01]**
Other children only	− 0.01 [− 0.08, 0.05]	0.14 ** [0.05, 0.23]	− 0.01 [− 0.07, 0.06]	− 0.04 [− 0.10, 0.02]
Other adults only	0.04 [− 0.00, 0.07]	− 0.02 [− 0.07, 0.04]	0.05 ** [0.02, 0.09]	0.04 ** [0.01, 0.08]
Alone (ref)				
N	14,892	14,891	14,891	14,892
Pseudo-R^2^	0.38	0.22	0.36	0.39
AIC	38,848	50,088	39,067	36,171

Fully adjusted linear mixed effects model (Step 3) results for each well-being outcome including natural and environmental features, exposure and experience. Full results, including covariates, are in Tables S4–S7. Note that a negative co-efficient for anxiety indicates disagreeing that the visit made them anxious.

Models adjust for age, sex, perceived financial strain, limiting illness/disability, garden access, employment status, educational attainment, ethnicity, relationship status, dog ownership, survey wave, underlying well-being, travel origin, travel mode, travel time and country (random effect).

***p < 0.001; **p < 0.01; *p < 0.05.

Significant values are in [bold].

The five bluespace types associated with significantly higher visit satisfaction than the grand mean were predominantly coastal environments—sea cliffs; rocky/stony shore; sandy beach/dunes; and seaside promenade—and rural river/canal. Of these, only sandy beach and rocky shore had significantly higher scores for happiness, worthwhile-ness and satisfaction, and lower levels of visit anxiety, than the grand mean.

Visiting outdoor pools, waterfalls or fen, marshes or bogs were associated with significantly worse levels of visit well-being than the grand mean, with higher levels of visit anxiety and lower levels of happiness, satisfaction, and worthwhile-ness (Fig. 2, Tables 1 and S4–S7). Visits to the open sea, harbour or marina, ice rinks, lakes, piers, small water bodies, fountains, and salt marshes were not found to be significantly different to the grand mean for any of the well-being responses.

##### 1b) Bluespace qualities

Of the environmental characteristics, safety consistently showed the strongest relationship with the mental well-being outcomes, with significant positive relationships with happiness, worthwhile-ness and satisfaction; and a significant negative relationship with anxiety (increasing safety related to reducing anxiety). In general, the other characteristics exhibited significant, but much weaker, relationships with each of the three positive well-being outcomes.

**Figure 3:**
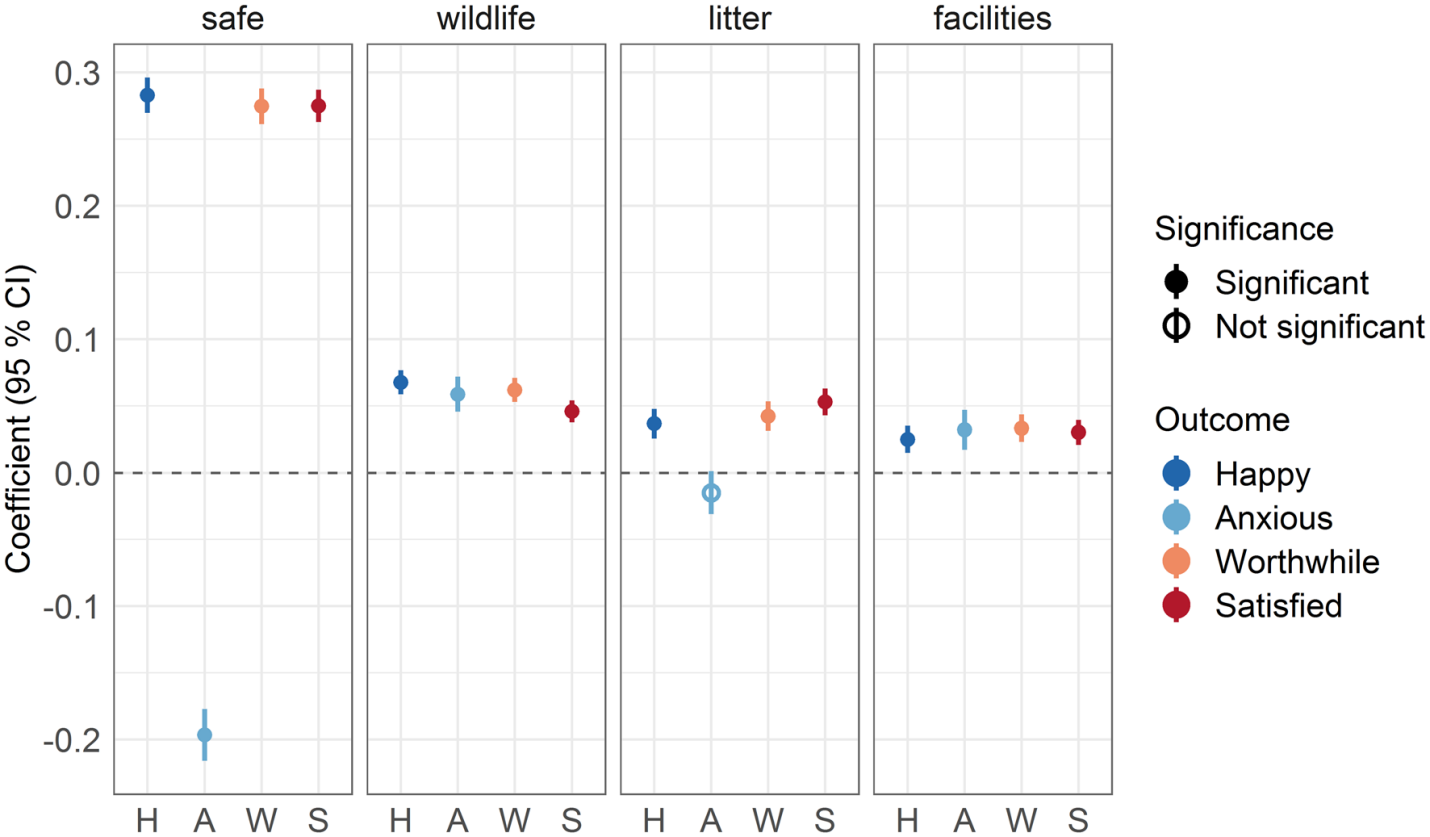
Well-being outcomes associated with perceived environmental qualities. Fully adjusted linear mixed effects model (Step 3) coefficients for the environmental qualities perceived safety, perceived presence of wildlife, perceived absence of litter and perceived presence of good facilities and each well-being outcome. Significance is demonstrated where the 95% CI does not cross 0 (displayed with a dashed horizontal line). Note that “H”, Happy, “A”, Anxious, “W”, Worthwhile and “S”, Satisfied.

The pattern with anxiety differs slightly, in comparison to the other three outcomes. Specifically, the greater the perceived presence of wildlife and the better the facilities, the *higher* the self-reported anxiety was on the visit. The absence of litter/vandalism was not statistically significantly related to anxiety, although the point estimate was in the expected direction (Fig. 3).

Perceptions of water quality were also associated with mental well-being outcomes. Compared to visits associated with “sufficient” water quality, visits where water quality was perceived as ‘excellent’ were associated with significantly greater happiness (*β* = 0.20; 95% CI = 0.15, 0.25), worthwhile-ness (*β* = 0.16; 95% CI = 0.11, 0.20) and satisfaction (*β* = 0.20; 95% CI = 0.15, 0.24), as well as significantly lower anxiety (*β* = − 0.11; 95% CI = − 0.18, − 0.04; Table 1). Perceived “good” water quality was also associated with significantly higher levels of visit happiness (vs.* “*sufficient”; *β* = 0.05; 95% CI = 0.01, 0.09) and “poor” water quality was associated with significantly lower levels of visit satisfaction (vs. “sufficient”; *β* = − 0.10; 95% CI = − 0.15, − 0.04).

#### Research question 2—exposure & well-being

Visit duration was positively associated with happiness, worthwhile-ness and satisfaction, and negatively associated with anxiety (Table 1). Compared to short visits of < 30 min, all duration categories of 30 min or more were associated with sequentially better well-being outcomes such that the longest visits (≥ 180 min) were associated with the most positive outcomes (happiness: *β* = 0.45; 95% CI = 0.39, 0.51; anxiety: *β* = − 0.23; 95% CI = − 0.32, − 0.15; worthwhile: *β* = 0.51; 95% CI = 0.45, 0.57; satisfaction: *β* = 0.45; 95% CI = 0.39, 0.50; Tables 1, S4–S7). However, anxiety was only significantly lower for visits 90 min or longer compared to < 30 min.

#### Research question 3—experience & well-being

##### 3a) Activity

Well-being outcomes for visit activity were modelled with sum contrasts (the same approach as for bluespace types), with coefficients representing the comparison with the grand mean. Playing with children and socialising were associated with significantly better well-being compared to the average visit for all outcomes (e.g. happiness, playing with children: *β* = 0.24, 95% CI = 0.17, 0.31; socialising: *β* = 0.12, 95% CI = 0.07, 0.18). Conservation activities, by contrast, were associated with significantly lower levels of visit happiness and significantly higher levels of visit anxiety compared to the grand mean (happiness: *β* = − 0.4, 95% CI = − 0.66, − 0.16; anxiety: *β* = 0.63, 95% CI = 0.26–1.00), though they were unrelated to visit worthwhile-ness and satisfaction.

Walking with a dog was associated with significantly better well-being for three measures, while winter activities, running, sport and other were associated with significantly worse well-being for three measures. Appreciating nature, sunbathing/paddling, eating/drinking and quiet activities were all associated with significantly lower levels of visit anxiety compared to the grand mean, and, of these, sunbathing/paddling and quiet activities were also associated with significantly higher levels of visit happiness than the grand mean (Tables 1, S4–S7).

No significant differences in mental well-being outcomes compared to the grand mean were found for fishing, watersports, cycling, swimming or visiting an attraction. Overall, variation in well-being outcomes as a function of activity type was greater than as a function of bluespace type.

**Figure 4:**
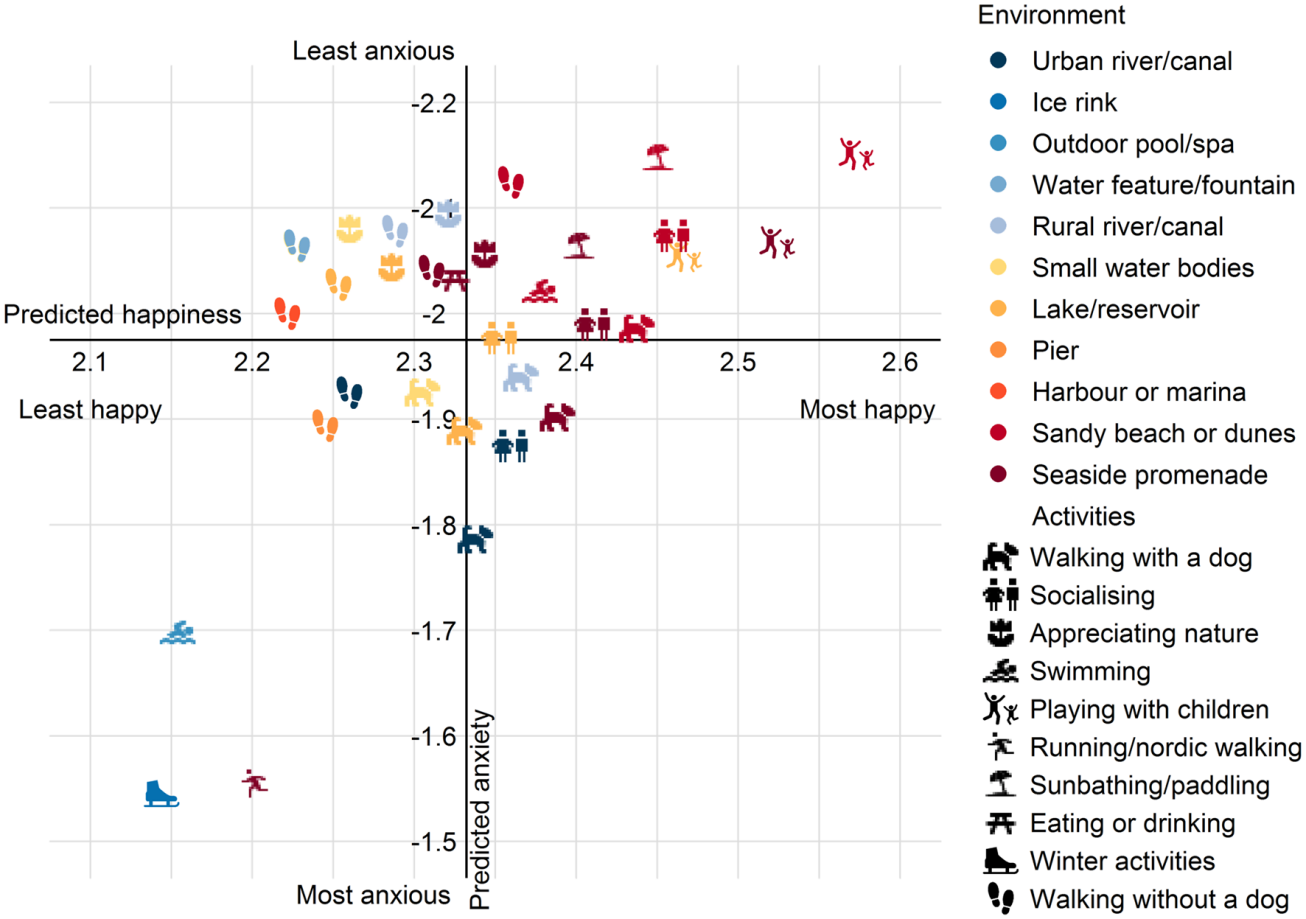
Predicted well-being outcomes by bluespace and activity. Predicted visit anxiety and happiness for combinations of activity and bluespace type where there were at least 100 visits. Predictions are based on fully adjusted models (Step 3) with varying activity and bluespace type. Predictions are the mean predicted anxiety and happiness from 2000 runs for each combination of activity and bluespace type. Environmental qualities, visit duration, travel characteristics and WHO-5 well-being index are specified when predicting with fixed values. All other variables are specified by drawing randomly from the distribution of the full dataset (see method). The origin of the x and y axes are the mean predicted happiness and anxiety respectively. Note that predicted visit anxiety is displayed with a reversed scale such that better well-being is at the top.

To analyse these relationships further, we visualised the combined effect of location and activity on happiness and anxiety. For robustness, we focused on activity-environment combinations with a minimum of 100 visits (excluding “other”) and predicted values of hedonic well-being in terms of happiness and anxiety based on the fully-adjusted models (Step 3) with covariates randomly drawn from the original distribution for visitor characteristics and fixed covariates for the remaining variables. These were plotted on axes of predicted happiness and anxiety, with origins of the axes set at the respective mean predicted values. The axis for visit anxiety is reversed so that, more intuitively, “better” well-being outcomes are displayed in the top right quadrant of the figure (Fig. 4).

With the exception of playing with children at lakes or reservoirs, the visits in the top right quadrant are all located at coastal environments and included appreciating nature, swimming, walking with and without a dog, playing with children, socialising, and sunbathing/paddling. Playing with children consistently exhibited the highest levels of predicted happiness. Playing with children and sunbathing/paddling at a sandy beach were also associated with the least anxiety.

Winter activities at an outdoor ice-skating rink were associated with the lowest levels of happiness and greatest anxiety. Running at a seaside promenade was also associated with lower than average levels of well-being (there were < 100 instances of running in other bluespace settings so comparisons were not possible). Outcomes varied with the environment, activity and well-being type. For example, swimming was associated with relatively high levels of well-being at a sandy beach, but lower than average levels of well-being in an outdoor public pool. Walking was generally associated with lower than average levels of anxiety, but also often lower than average levels of happiness.

##### 3b) Companions

Compared to visiting alone, visiting with other adults was related to both higher levels of worthwhile-ness (*β* = 0.05; 95% CI = 0.02, 0.09) and visit satisfaction (*β* = 0.04; 95% CI = 0.01, 0.08), while levels of visit anxiety and happiness were not significantly different. Visiting with both other adults and children (*β* = 0.23; 95% CI = 0.16, 0.29) and just children (*β* = 0.14; 95% CI = 0.05, 0.23) was associated with significantly greater anxiety than visiting alone.

Although this appears to contradict the results in Fig. 4, where playing with children had high happiness and low anxiety, the overall pattern reflects the fact that most visits with children did not involve playing with children as the main activity (Table S2). Visiting with other adults and children was also associated with significantly lower levels of visit happiness (*β* = − 0.05; 95% CI =− 0.10, − 0.01) and visit satisfaction (*β* = − 0.05; 95% CI = − 0.09, − 0.01) than visiting alone, although this was not the case for visiting with children only.

#### Visitor and travel characteristics

Full results are found in Supplemental Tables 4–7. Being male (vs. female), being limited by an illness or disability (vs. no limiting illness/disability) and having access to a communal garden (vs. no access to any garden or outside space) were consistently associated with worse mental well-being outcomes. However, contrastingly, reporting that they did not work due to disability was associated with better well-being outcomes (vs. being employed).

Better general well-being (as measured with the World Health Organization five-item index) was also generally associated with better visit well-being (apart from anxiety), as was increasing age (apart from happiness). Increasing travel duration was also consistently associated with worse mental well-being outcomes along with starting the travel from work (vs. home).

#### Research question 4—wildlife and well-being across different settings

**Figure 5:**
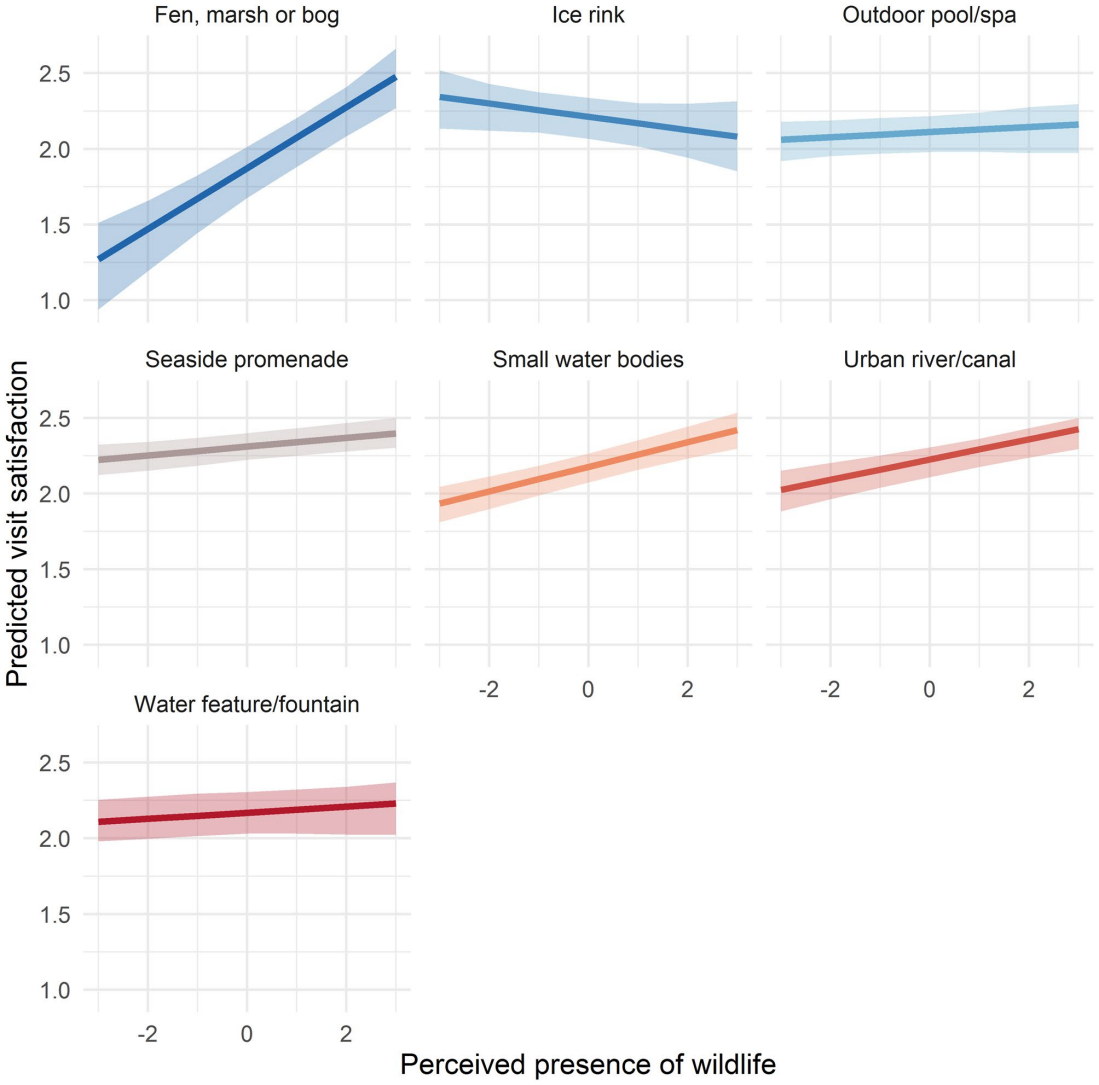
Predicted visit satisfaction by bluespace and wildlife. Predicted visit satisfaction (with 95% confidence interval) estimated from a fully adjusted linear mixed effects model with an interaction effect between wildlife and bluespace type. Other variables were fixed where safety, litter and facilities = 2; water quality = sufficient, proximity =  < 15 min travel; visit duration = 1–1.5 h; activity = walking without a dog; companions = alone; travel mode = private vehicle; start point = home; financial strain = coping; age = 18–29; sex = male; presence of limiting illness/disability = no; garden = no access; employment status = employed; educational attainment = university; member of ethnic minority = no; relationship status = married/cohabiting; dog ownership = no; wave = June 2017; country = United Kingdom and WHO-5 well-being = 80. Environments displayed where the interaction was significant in comparison to the grand mean for bluespace x wildlife (Table S8).

High presence of wildlife was associated with higher visit satisfaction. Research question 4 aimed to explore whether this relationship varied across bluespace settings by testing additional models with interaction terms. Table S8 presents the full results, while Fig. 5 presents a selection of these results where the interaction effect was statistically significantly different to the grand mean associated with bluespace type by perceived wildlife presence.

The strongest relationship (steepest slope) between perceptions of wildlife presence and visit satisfaction was for the wetland types of fen, marsh, and bogs. Both small water bodies and urban rivers/canals showed slightly stronger associations than the average. Weaker than average associations between the presence of wildlife and visit satisfaction were found for some settings, such as seaside promenades and water features. At outdoor ice rinks, greater perceived presence of wildlife was associated with lower visit satisfaction.

## Discussion

We investigated the complex relationships between the environmental characteristics of blue spaces and visit-related mental well-being in a multi-country study including 17 bluespace types and four facets of subjective well-being. Our aim was to operationalise, and consider the utility of, the Bratman et al.^9^ conceptual model that links ecosystem services (ESS) with mental health.

Consistent with the proposed conceptual model, mental well-being outcomes relied on a complex interplay of individual, environmental, and visit characteristics.

### Summary of findings

Overall, bluespace visits were associated with better subjective mental well-being outcomes if the visits took place in nearby coastal areas or rural rivers, were perceived as safe and to have good water quality, and had a long duration. They could involve a range of activities such as playing with children, socialising, or walking. The degree to which the perceived presence of wildlife predicted visit satisfaction varied depending on the bluespace type, suggesting that the importance of ecosystem features such as biodiversity may vary by the setting.

We can also identify the combination of environmental and visit characteristics associated with particularly high levels of well-being for a specific outcome. For example, an optimal visit in terms of happiness might be to sandy beaches where there are high levels of perceived safety and excellent water quality; with a visit lasting at least three hours; and possibly involving playing with children, socialising, sunbathing/paddling and/or walking with a dog; and has short travel times that do not involve public transport.

### RQ1—natural and environmental features

#### Research question 1a—Which bluespace type(s) were associated with the highest levels of recalled visit mental well-being?

Four of the five bluespace types associated with the highest levels of visit satisfaction were coastal (sea cliffs, rocky shore, sandy beaches, rural river and seaside promenade), indicating that these environments may be particularly beneficial for well-being. Visits to these environments were also associated with the lowest levels of visit anxiety, with the exception of seaside promenade and sea cliffs, which were not significantly different to the grand mean. Seaside promenade was the only urban environment in the top five.

In addition, only coastal sites were associated with significantly higher levels of visit happiness (compared to the grand mean), further highlighting the potential importance of these environments. Although not explored here, coastal scenes tend to be associated with particularly high aesthetic and scenic value^25,26^ which may also be positively related to subjective well-being.

These findings are broadly consistent with other studies from the UK^17,27^, but are extended here to our international sample. White et al.^28^ also used data from the BlueHealth International Survey (BIS) and explored visit frequency to different environments and associations with general mental health and well-being outcomes, including the World Health Organisation five-item Well-being index referring to the two weeks prior to the survey. Consistent with the results here, they found that visit frequency to “coastal blue” environments was more strongly associated with psychological well-being in general than visit frequency to “inland blue” environments. Our study adds to these more general findings by showing that these associations may come as a direct result of the recalled well-being experienced on specific visits to these locations.

Confidence in our results was strengthened as we included general mental well-being in our analysis to adjust for whether happier people tend to visit sandy beaches, for example. The results for visit anxiety were not always the inverse of the trends observed in the positive measures of well-being, supporting the need to look at multiple aspects of mental well-being when considering the effects of nature contact.

#### Research question 1b—Which bluespace qualities were associated with the highest levels of recalled visit mental well-being?

Of the range of qualities that we investigated as predictors, perceived safety and ‘excellent’ water quality (vs. ‘sufficient’) consistently exhibited the strongest relationships with subjective mental well-being. Perceived safety has been found to be important when visiting blue spaces in several qualitative studies^29–31^, as well as a quantitative study with older adults in Hong Kong^14^. Blue spaces have particular safety issues with respect to drowning^32,33^, but fear of crime^29,30,33^ or pedestrian safety^34^ may also be relevant.

Water quality has also been found to be important in previous economic valuation studies of recreational use and enjoyment of lakes and estuaries in the USA and Australia^35,36^ as well as a contingent behaviour experiment carried out as part of the BlueHealth International Survey (in European countries only)^37^. We recognise that here we used a metric of perceived water quality, rather than measures based on biological or toxicological sampling. Nevertheless, perceptions have been reported to positively correlate with sampled water quality parameters^38^, although assessments can vary systematically such as by bluespace type^39^. Highly visible harmful algal blooms, for instance, have also been found to affect experiences of blue spaces^40^.

Further, and again consistent with earlier work^15,41,42^, the presence of facilities and wildlife, and absence of litter, were generally associated with better subjective mental well-being. Both perceived presence of wildlife and facilities were also associated with higher levels of anxiety however, indicating complexities between environmental qualities and well-being. Some wildlife may be deemed unpleasant or an ecosystem disservice, for example. The presence of good facilities may indicate the presence of more people; and visitor density in natural environments can be related to preference^43^. These results highlight the importance of environmental quality and not just type, consistent with other frameworks^12,37^.

#### Research question 2—How is exposure, as operationalised by visit duration, related to recalled visit mental well-being?

Broadly consistent with research in the green and bluespace literature^14,17,44^, we found that mental well-being outcomes were generally higher with greater exposure as indicated by visit duration. For decreasing visit anxiety, this was only significant when visits were longer than an hour and a half. As we did not measure pre-visit anxiety levels, we are cautious about identifying this as a potential temporal threshold for reducing anxiety at this stage.

Similarly, also using the BlueHealth International Survey, White et al.^28^ found that well-being outcomes were higher with greater visit exposure to green and blue spaces using a metric of visit frequency. However, in contrast to this and other research which looked at overall weekly aggregated time in nature (e.g.^28,45^), we have no evidence of diminishing marginal returns as the effect sizes associated with specific visit duration continued to increase with increasing duration.

#### Research question 3—What experiences in blue spaces, in terms of activities (3a) and companions (3b), are associated with the most positive recalled visit mental well-being outcomes?

Although walking was the most popular activity, the activity with the highest mental well-being ratings was playing with children, especially in certain locations such as beaches (Fig. 4). However, we also find that anxiety tended to be higher when children were present. We speculate that the purpose of the visit may be important. For example, many who go to the beach with children do so in order to play. However, if children are present on more adult-oriented activities such as hiking, this may increase adult anxiety during the visit. From a representative sample of English adults, White et al.^17^ found that recent nature visits with children were associated with the lowest levels of well-being. Therefore, visits with children may be associated with a more complex set of emotions, being both slightly more stressful, but also potentially more rewarding and ‘meaningful’^46^. Ecosystem features of beaches may be particularly supportive of high well-being activities. A qualitative study in the UK, for instance, highlighted the particular opportunities for adults and children to play together at the beach, including rock-pooling and making sandcastles as well as water-based activities^47^.

Visits with other adults were associated with higher levels of both visit satisfaction and worthwhile-ness, and socialising as an activity was associated with better visit well-being for all outcomes compared to the grand mean. This is consistent with studies using the day reconstruction method, which link activities with experiential well-being, in the USA^48^ and Germany^49^ where socialising was associated with the highest, or second highest, levels of well-being for all the activities assessed. Further, social interactions have been recognised as an important benefit by many of those visiting freshwater blue spaces in a previous study^18^.

#### Research question 4—Does the relationship between wildlife presence and recalled visit well-being vary by bluespace settings?

The relationship between the presence of wildlife and visit satisfaction varied with bluespace type. The strongest positive association was found for fen, marsh and bog areas, which may also be related to the purpose of visit. For instance, those who visit places such as fens, marshes and bogs, may do so for the explicit purpose of observing wildlife (often birds) and the presence of wildlife would therefore be important for satisfaction with the visit.

Perceptions towards wildlife have been found to vary by location in other studies. For example, in Sweden, greater prior experience with geese at beaches was associated with a negative attitude towards geese^50^. Further, the species present are likely to vary across different environments. In three urban areas in the UK, green spaces correlated with the abundance and species richness of birds considered to provide cultural services (songbirds and woodpeckers), while an abundance of birds considered to provide disservices (e.g. some gull species, feral pigeons) was independent of green spaces^51^. Preferences for some species over others may explain some of the negative or null relationships between the presence of wildlife at different blue spaces. These examples from the literature, alongside our own results, indicate the potential for benefits from the management of wildlife for psychological ecosystem services differentially across environments, although these should be considered alongside other conservation and ESS goals.

### Mechanisms

Several mechanisms potentially explain the beneficial effects of visiting blue spaces on mental health and well-being^12^, including the provision of opportunities for physical activity^52,53^; social interaction^18^; cognitive restoration and stress reduction^17,54^; emotion regulation^55^ and connecting with nature^12^. Consistent with these mechanisms, we found that respondents were using blue spaces for both physical activity and social interaction; and that playing with children and socialising were associated with particularly high levels of well-being.

In addition to the positive association we find between some ESS and well-being, including presence of wildlife and water quality, additional bluespace ESS not considered here, may also affect mental health and well-being^12^. For example, the provision of a cooling effect^56^ and air pollution mitigation^57^.

### Strengths and limitations

A key strength is our operationalisation of the Bratman et al.^9^ conceptual model for mental health using data from a large, 18 country survey that included 17 different bluespace types, five quality metrics and four subjective mental well-being outcomes. The relatively high explanatory power of our models suggests all the variables we explored were important for subjective well-being.

Despite the strengths, however, there were also several limitations. The survey was cross-sectional and causality cannot be inferred. For example, happier people may choose to visit a beach rather than another location, although we also controlled for general levels of subjective mental well-being in an attempt to control for this possibility (See Supplemental Materials). Further, although the majority of respondents (53%) recalled a visit within the last 7 days, some were recalling visits up to a month ago, with potential memory biases increasing in line with length of recall.

Although our data were collected by an international market research company to be representative of age, gender and region within country, our online sample may not be fully representative across more characteristics and any country-level conclusions need to be treated with caution. We also acknowledge that there were no results from Africa, the Middle East or South America; and Hong Kong was the only representative from Asia. This suggests far more research is needed in other regions to better understand how bluespace ecosystems interact with subjective well-being globally.

There may also be socioeconomic confounds that we did not include in our models which may account for some of the effects. Not everyone visits nature for recreation^58^, including about 4000 people here who did not visit a bluespace in the four weeks prior to responding to the survey. Some groups may therefore have been under-represented; and we should be careful in assuming that our findings generalise to all sub-population groups.

Nevertheless, our visit sub-sample distributions were generally similar to that of the weighted percentages in the full sample, with the exception of age where those aged over 60 were under-represented (Table S2); therefore, we suspect these issues were not too influential for the overall results, although care needs to be extended to inferences with respect to older adults.

A further limitation was that we only considered the qualities of places where people reported making recreational visits, with respondents presumably less likely to visit places where they feel really unsafe or lacking in facilities^29^. Further research may want to study responses to a broader range of bluespace settings, including those that are less visited, to determine the generalisability of the generally positive results found here. Such studies could use pre-existing tools to objectively assess the quality of blue spaces^59^.

### Implications

Our finding that coastal environments are particularly beneficial adds to the body of evidence linking coastal environments with health and well-being and suggests this is consistent across many countries. Previous research has found that greater proximity to blue spaces, especially coastal settings, predicts visit frequency^14,60,61^ as well as other health outcomes—e.g. reduced risk of mortality and better general health, well-being and physical activity^53,62^. Here, we found that shorter travel times also predict visit well-being, highlighting the importance of having equitable access to good quality natural environments near to people’s homes.

We also identified that different types of coastal and inland blue spaces (e.g. seaside promande, rural rivers), with different qualities (e.g. wildlife present), involving particular types of activities in specific social configurations (e.g. playing with children), were especially good at promoting well-being. This moves beyond a simple location-based assessment of benefit to one that recognises the complex interplay between location, behavioural and social processes. Numerous commentators^63^ (including Bratman et al.^9^ on which we have based this paper) have argued that we need to go beyond the determinate effects of green and blue spaces and develop a far richer, more nuanced understanding. The approach we have taken here is intended as a step in this direction.

In terms of policy applications, these results provide support for the potential health benefits of efforts to improve equitable access to high quality environments, such as the English Coast Path (https://englandcoastpath.co.uk/) and the creation of beaches in Barcelona with the Olympic project in 1992^64^. Our results also hint at the importance of high-level legislation, such as the EU’s Bathing Waters Directive^65^ for mental well-being^37^. If conducted on a more fine-grained geographical level, results could have the potential to leverage public support for more localised conservation initiatives. Furthermore, such results could be used as a basis for integration into more systematic conservation planning^66^.

### Further research

Although we incorporate a range of variables in our analysis, and our *pseudo-R*^2^ values are relatively high for a social research context, considerable variation remains unexplained. Although other individual characteristics may be important, such as nature connectedness^67^ and memories^68^, further research could explore the specific ecosystem features and social contexts associated with the particular positive results from coastal spaces, which would be of interest to policy makers and environmental managers. We also speculated that purpose of visit may explain some of our findings. Further research could explore the interactions between motivations and location, experience, and well-being outcomes.

The presence of wildlife was differentially important across bluespace types and further research could unpack this. Exploring similar possibilities for the other quality metrics, as well as considering additional ecosystem characteristics, would also be informative. For example, identifying which factors are important in perceptions of safety in blue spaces. Bratman et al.^9^ also considered effect modification by visitor characteristics and further research could include interactions, or sub-group analysis, by socio-demographic factors.

Further research could also explore longer-term benefits of these features over repeated visits; the potential for ecosystem disservices, such as the relationships we find between an interaction of wildlife and ice rinks and well-being; the potential for negative outcomes associated with ecosystem degradation^69^; and the potential for positive mental health outcomes from ecological restoration^70^.

We have demonstrated some of the complexities involved in the human-nature relationship and that many factors are related to the outcome from a visit. The conceptual model applied allows the investigation of a wide range of variables including natural features and other environmental qualities, and characteristics of the exposure and experience, as well as individual parameters. We suggest that other researchers can apply this conceptual model and design data collection accordingly to target specific research questions and hypotheses (as opposed to where we have fitted already collected data).

## Conclusions

We highlight the complexity of mental well-being outcomes associated with different aspects of visits to blue spaces. We used the ESS-mental health conceptual model to inform an exploration of these complexities in a structured way. While it is increasingly recognised that nature exposure can improve health and well-being, not all visits are equally beneficial. A range of environmental, visit and travel characteristics, as well as socio-demographic attributes, were all found to predict mental well-being outcomes.

Optimal mental well-being outcomes from bluespace visits may result from visits which are coastal, high quality, safe, several hours in duration, nearby, and involve activities such as walking, socialising, or playing with children. However, we also recognise that individual circumstances, preferences and motivations to visits vary, as well as sociocultural contexts; and that all options will not be available to, or beneficial for, everyone.

The study highlights the utility of using the ESS conceptual model for detailed research into the ways in which nature impacts the mental health and well-being of people differently, depending upon the type and quality of the nature, the details of the exposure and experience, and the characteristics of the visitors themselves.

## Materials and methods

### BlueHealth

This research was part of the BlueHealth project which, as a whole, aimed to “*understand the relationships between exposure to bluespace and health and well-being, to map and quantify the public health impacts of changes to both natural blue spaces and associated urban infrastructure in Europe, and to provide evidence-based information to policymakers on how to maximise health benefits associated with interventions in and around aquatic environments*”^10^. This project recognised that while risks and hazards associated with water were relatively well understood, the potential benefits were less well known^10^. The project hypothesised that many of the pathways providing benefits would be similar to those of green spaces, but there would also be differences. The project developed the bespoke BlueHealth International Survey (BIS)^10,11^.

### Ethics

The content and proposed methodology of the survey was approved by the University Of Exeter College Of Medicine and Heath Research Ethics Committee (reference number 16/06/099).

### Participants and sampling procedure

Respondents from 18 different countries/territories were surveyed using an online self-report survey administered by international market research company, YouGov. The 18 countries/ territories consisted of 14 European countries (the UK, Bulgaria, Czech Republic, Estonia, Finland, France, Germany, Greece, Ireland, Italy, the Netherlands, Portugal, Spain and Sweden); and four countries/regions outside of Europe (Canada, California (USA), Hong Kong (China), and Queensland (Australia)).

In each country or region, a representative sample of approximately 1,000 people were surveyed. Typically, sampling stratification was by geographic region, gender, and age group; though this differed in some countries/territories due to feasibility given the number of registered online panellists within given strata. The survey was administered in four seasonal waves across a year commencing in June 2017. Full details can be found in^11^. The total sample size was 18,838.

The survey first consisted of an information page including the aim of the survey: “*to find out how people use outdoor spaces internationally. These can be green spaces such as parks and the countryside, or blue spaces such as the coastline, lakes and rivers (*i.e.* including water)*.” Participants were asked for their consent and informed they could withdraw at any time. All methods were performed in accordance with the relevant guidelines and regulations. Further details on YouGov’s sampling approach and their adherence to international industry standards for data collection and storage can be found here https://yougov.co.uk/about/panel-methodology/, which includes a link to their detailed response to the World Association for Social, Opinion and Market Research (ESOMAR) guidelines on Online research.

The survey consisted of seven modules, the first four queried subjective well-being; frequencies of visits to different natural environments; general perceptions of neighbourhood natural environments; and details on the most recent visit to a bluespace. Those who responded that they had visited a bluespace at least once in the last four weeks (n = 14,998; 81.6%) were asked to report on their most recent visit. Nineteen percent of visits were on the same day or the day prior to completing the survey; and 53% were within the previous week. Full details of the survey can be found in Elliott and White^11^.

### Outcome measures

Participants were asked about the mental well-being outcomes of their visit, which corresponds with Step 4- “*Effects*” in Bratman et al.^9^. On seven-point Likert items from strongly disagree (− 3) to strongly agree (+ 3), participants were asked the extent to which the visit made them feel happy, anxious, how worthwhile they found the visit, and how satisfied they were with the visit. These correspond to positive and negative affect, eudaimonic, and evaluative well-being measures (respectively) recommended by the OECD for national subjective well-being measurement^20,21^. A composite measure of these items has been shown to be positively associated with bluespace visit duration and activity intensity in an older-adult sample in Hong Kong^14^. Details of all the variables and their categorisations are given in Table S1.

### Predictors

#### Natural and environmental features

##### Bluespace type

Respondents were asked to select one option from a list which best describes the place they visited for the majority of the time. These were: fen, marsh or bog; harbour or marina; natural or artificial lake or reservoir; open sea; ornamental water feature or fountain; outdoor public pool, lido or thermal spa; outdoor ice skating rink or ice hockey rink; pier; rocky or stony shore; rural river/canal (with vegetated banks); salt marsh, estuary or lagoon; sandy beach or dunes; sea cliffs; seaside promenade; small water bodies (e.g. streams and ponds); urban river/canal (surrounded by buildings); and waterfalls or rapids (missing n = 4). These categories have been slightly adapted from the bluespace typology presented in Bell et al.^71^.

##### Bluespace qualities

Respondents were also asked about their perceptions of the environmental qualities of the bluespace they visited. They were asked to state how much they agreed with four statements: “*I felt safe”, “there was wildlife to see and enjoy”, “the area was free from litter/vandalism” and “there were good facilities (e.g. parking, footpaths, toilets)*”. These responses were also 7-point Likert items from strongly disagree (− 3) to strongly agree (+ 3). Missing values ranged from 5 to 13. The presence of good facilities and wildlife at urban blue spaces has been shown to be related to bluespace visit frequency in Hong Kong^14^.

In addition, respondents were asked to “*rate the quality of the water at the bluespace you visited? Think about the colour, smell, any litter that was in the water *etc.” with four response options of poor, sufficient (ref), good or excellent which correspond with the EU bathing water quality designations^24^.

Of these, we selected the interaction between presence of wildlife and bluespace type to explore in more detail, as an example of the types of analyses that can be conducted. We selected this variable given the importance of species diversity in ESS frameworks and the relatively small literature on the relationship between biodiversity and promotion of mental health^22,23^.

#### Exposure

##### Visit duration

In line with Step 2 of Bratman et al.^9^, where it was recommend that frequency and duration of exposure should be considered, we include ‘visit duration’ as it is particularly relevant to the context of a specific visit to a bluespace. Respondents were asked* “*approximately how much time did you spend at that bluespace?” with responses options in 10-min intervals up to “*4 h or more*”. Numbers of responses were skewed towards standard durations such as 1 h or 1 h and a half. Therefore, durations were categorised to < 30 min (ref), 30–< 60 min, 1–< 1.5, 1.5–< 2 h, 2–3 h and greater than or equal to 3 h (missing n = 7). Longer visit durations have been found to be positively related to recalled well-being on bluespace visits in Hong Kong^14^ and in England^17^.

#### Experience

##### Visit activity

Respondents were asked to choose the main activity on the visit from a list, and state how many adults and children were with them on the visit. Activities included were informed by previous surveys^72^; and underwent consultation across multiple countries within the project consortium and public engagement groups to ensure the list was comprehensive and culturally sensitive. Where the potential for ambiguity existed, examples were given (e.g. ‘*adventure sport*’ examples included coasteering, climbing, paragliding, off-road driving, and mountain biking).

These activities were further categorised for this analysis as they originally consisted of 30 categories, some with very small sample sizes (min n = 21 [hunting]). They were categorised subjectively according to similarity between activities and maintaining activities of particular interest (such as those with direct water contact e.g. fishing; see Table S1).

##### Visit companions

Companions on the visit were categorised according to the number of adults and children present (with other adults only, other children only, other adults and children or alone (ref); missing n = 35). Children companions have been previously associated with reduced restoration on nature visits^17^.

#### Visitor characteristics

All socio-demographic variables were categorical (with full details of all categories in the Supplementary Materials; Table S1). These were: age (ref = 18–29), sex (ref = female), perceived financial strain (ref = coping on present income), limiting long-term illness/disability (ref = no), garden access (ref = no access), employment status (ref = employed), highest educational attainment (ref = university), minority ethnic group member (ref = no), marital status (ref = not married/living with a partner), dog ownership (ref = no) and country of residence (ref = UK). We also included survey wave to account for seasonal variation (ref = June 2017 ≈ summer in the northern hemisphere). Country was included as a random effect.

Well-being, mental health, or nature visit frequency/likelihood have been found to vary with these characteristics^17,18,20,27,58,73,74^. Missing values for perceived financial strain (missing n = 268) and relationship status (missing n = 861) were retained as “missing”; and, due to there being a relatively high number, these respondents’ data were included in analysis under a “missing” category heading.

We added a measure of general subjective mental well-being to at least partially account for the possibility that people predisposed to feeling more positive about their quality of life visited certain types of places, which might distort results. For example, if generally happier people visit coastal promenades, then an association between promenades and visit happiness might be due to differences in general well-being rather than resulting from the location itself. For instance, prior stress has been found to moderate psychological and physiological responses to a bluespace visit^54^. By accounting statistically for these dispositions, therefore, we reduce the possibility of this potential confound affecting results.

To do this we used the World Health Organisation five-item Well-being Index^75^. Respondents were asked to consider, for the last two weeks, how often they felt cheerful and in good spirits, calm and relaxed, active and vigorous, awaking fresh and rested, and that daily life is filled with interesting things. Responses are on a scale from at no time (= 0) to all of the time (= 5). These values are then added together and multiplied by 4 to give a maximum score of 100^75^ (missing n = 0).

#### Travel characteristics

Respondents were asked from where their journey started, with home (ref), work, holiday accommodation, or elsewhere as response options (missing n = 4). These questions were adapted from the English Monitor of Engagement with the Natural Environment (MENE) survey^72^. They were also asked how they travelled to the site with 10 options collapsed into private vehicle, on foot/bike (including wheelchair/mobility scooter use; ref), public transport and other (missing n = 11) for analytical purposes. Finally, respondents were asked: “*Approximately how long was your total journey time from your start point to the bluespace you visited*?” as an open (numeric) question. The responses were right-skewed and were thus categorised: < 15 min (ref), 15–< 30 min, 30–< 60 min, 60–< 120 min, and >  = 120 min (missing n = 7). Details of all the variables and both the survey response options and categories are in Table S1.

### Analysis

All analyses were carried out in RStudio (Version 2022.02.3 Build 492 (2009–2022) RStudio, Inc.) with R version 4.1.0 (2021-05-18) (R Development Core Team 2019) using a linear mixed effects regression approach, including a random intercept for country (with packages “lme4”^76^ and “lmerTest”^77^). Much of the variation in outcome responses was at the higher values of recalled visit well-being outcomes, which we wanted to retain in analyses (and therefore have not implemented binomial models). It should be noted that there was considerable skewness in the outcome measures. However, with Likert scales, the results are typically consistent regardless of whether binomial or linear models are used and we also note that dichotomising data (into binary outcomes) can reduce the information in the data and thus reduce statistical power^78,79^. Further, considering an ordinal model for the outcomes typically makes little difference to results, compared to a linear (Gaussian) model when the response options are limited, while the assumption of underlying linearity produces results that are far easier to interpret^80^.

For both bluespace type and visit activity, we applied sum contrasts. This means that instead of the resulting coefficients being in comparison to a specified reference value (as they would be with treatment coding), they are in comparison to the grand mean (or the mean of means for each category). The intercept of the model is fixed to the grand mean rather than being estimated.

We added sets of variables in a multi-step process (Fig. 1; Table 2). Models were fitted for each of the four mental well-being outcomes separately (see Tables S4–S7 for RQ1-3 and Table S8 for RQ4 results).

**Table 2:** Model variables. Variables included at each model step.

Associated RQ		Natural and environmental features—Bluespace type	Visitor characteristics	Natural and environmental features—Bluespace qualities	Exposure	Travel	Experience	Interactions
		Results table column headers						
		Step 1a	Step 1 covariates	Step 1b	Step 2	Step 2 covariates	Step 3	
1a	Bluespace type	✓	✓	✓	✓	✓	✓	✓
	Visitor characteristic Perceived financial strain Age Gender Limiting illness/disability Garden access Economic status Educational attainment Ethnicity Marital status Dog ownership Survey wave Country (random effect) WHO-5 Well-being Index		✓	✓	✓	✓	✓	✓
1b	Perceived environmental qualities Safety Presence of wildlife Absence of litter Good facilities Water quality			✓	✓	✓	✓	✓
2	Exposure Visit duration				✓	✓	✓	✓
	Travel characteristics Travel duration Travel mode Travel origin					✓	✓	✓
3	Experience Visit activity Visit companions						✓	✓
4	Interaction Wildlife × bluespace type							✓

1. Unadjusted, bluespace type only (RQ1a)
2. Adjusted with visitor characteristics (including global well-being and a random effect for country).
3. Addition of bluespace qualities (RQ1b)
4. Addition of exposure variable (RQ2)
5. Additional adjustment with travel characteristics
6. Addition of experience variables (RQ3)
7. Additional interaction between wildlife and bluespace type (RQ4)

We provide the AIC and *R*^2^ values as indicators of explained variance. For the mixed effects models, the *pseudo-R*^2^ is provided, this is the value provided by the “export_summs” function in the “jtools” package^81^ and is calculated using the approach in Nakagawa and Schielzeth^82^. Missing values were excluded (except for financial strain and relationship status). The modelling sample therefore varied with each outcome and modelling stage. Final, modelling sample sizes (from Step 3 in the above process) ranged from 14,891 (anxious and worthwhile) to 14,892 (happy and satisfied). Normality of model residuals was assessed using visual inspection. The results for happiness, worthwhile and satisfaction showed no large discrepancies compared to a normal distribution, although the residuals for anxiety exhibited a small positive skew.

For those combinations of activity and environment reported on more than 100 visits, predicted values for visit anxiety and visit happiness were calculated based on the fully adjusted model (including all variables, corresponding to Step 3). Activity “other” was excluded. When predicting, the environmental qualities perceived safety, perceived absence of litter, perceived presence of good facilities and perceived presence of wildlife were fixed = 2 and perceived water quality was fixed at “sufficient”; visit duration = 1–< 1.5 h; travel mode = “private vehicle”; travel time =  < 15 min; travel origin = “your home”; and WHO-5 Wellbeing Index = 80. All other variables were drawn randomly from the distribution in the original data (“rmultinom” function). The mean predicted visit anxiety and visit happiness was calculated from 2000 runs of the prediction function for each combination of activity and environment. These mean values were then plotted on axes of predicted happiness and anxiety, with axes origins of the mean of means.

## Supplementary Information

Supplementary Information.

## Data Availability

For queries about the specific data and analysis, including the R Markdown code, used in the present manuscript please contact the corresponding author. Some of the data are governed by memorandums of understanding, but excerpts of the data (EU-funded; 11 countries) are available at the UK Data Service http://doi.org/10.5255/UKDA-SN-8874-2.
