# Supplementary material for: Applying an ecosystem services framework on nature and mental health to recreational blue space visits across 18 countries
Source: Sci Rep. 2023 Mar 6;13:2209. doi: 10.1038/s41598-023-28544-w (PMC9988977; doi:10.1038/s41598-023-28544-w)
Supplement: Supplementary file 1 — Supplementary Information. [file 41598_2023_28544_MOESM1_ESM.docx]

Supplementary materials

Table S1.

Variables and categories. See (1) for further details

| Variable | Question | Survey response options | Response categories (if different from survey response options) |
| --- | --- | --- | --- |
| **RQ1 Natural and environmental features** | | | |
| Blue space type | Which of the following best describes the type of blue space that you visited? Please only select one option which best describes the place you visited for the majority of your time | Blue space type |  |
|  |  | Fen, marsh or bog |  |
|  |  | Harbour or marina |  |
|  |  | Natural or artificial lake or reservoir |  |
|  |  | Open sea |  |
|  |  | Ornamental water feature or fountain |  |
|  |  | Outdoor public pool or thermal spa |  |
|  |  | Outdoor skating or ice hockey rink |  |
|  |  | Pier |  |
|  |  | Rocky or stony shore |  |
|  |  | Rural river/canal |  |
|  |  | Salt marsh, estuary or lagoon |  |
|  |  | Sandy beach or dunes |  |
|  |  | Sea cliffs |  |
|  |  | Seaside promenade |  |
|  |  | Small water bodies |  |
|  |  | Waterfall or rapids |  |
|  |  | Urban river/canal (ref) |  |
| **Environmental characteristics** | | |  |
| Perceived safety  Presence of wildlife  Presence of litter/vandalism  Presence of good facilities | How much do you agree with the statements below about your visit?  “I felt safe” (i.e. protected from danger)  “There was wildlife to see and enjoy”  “The area was free from litter/vandalism”  “There were good facilities (e.g. parking, footpaths, toilets)” | Strongly disagree  Disagree  Slightly disagree  Neither agree nor disagree  Slightly agree  Agree  Strongly agree | -3  -2  -1  0  1  2  3  (numerical) |
| Water quality | How would you rate the quality of the water at the blue space you visited? Think about the colour, smell, any litter that was in the water *etc.* | Poor  Sufficient (ref)  Good  Excellent | Categorical |
| **RQ2 ‘Exposure’ variables** | | |  |
| Visit duration | And approximately how much time did you spend at that blue space? | 10 minutes  20 minutes  30 minutes  *…..categories continue for every 10 mins…*  4 hours or more | <30 mins (ref)  30 mins - <1 hr  1 - <1.5 hr  1.5 - <2 hr  2 - <3 hr  >=3 hr |
| **RQ3 Experience** |  |  |  |
| Visit activity | On this visit which of these activities, if any, was the main activity you did | Walking with a dog  Walking without a dog  Nordic walking (i.e. with poles)  Running  Cycling  Horse riding  Golf  Adventure sport (e.g. coasteering, climbing, paragliding, off-road driving, mountain biking)  Informal games and sport (e.g. Frisbee, bat and ball, beach ball)  Fishing (including angling, crabbing)  Hunting or shooting  Conservation activity (e.g. litter-picking)  Sunbathing  Visiting an attraction  Quiet activities (e.g. reading meditating)  Playing with children  Appreciating scenery from a car  Eating or drinking  Socialising with friends  Watching wildlife  Boating (e.g. yachting, canoeing, kayaking, pedalo/paddle boat)  Commercial boat trip (e.g. organised fishing trip, marine wildlife trip)  Paddling (i.e. walking in shallow water)  Swimming  Watersport (e.g. surfing, windsurfing, kitesurfing, Jet Ski)  Diving (e.g. Scuba diving, snorkelling)  Ice skating  Ice fishing  Snow sports (e.g. skiing, snowboarding, cross-country skiing, sledding)  Any other activity not on the list | Walking with a dog  Walking without a dog  Running  Running  Cycling  Sport  Sport  Sport  Sport  Fishing  Other  Conservation  Sunbathing/paddling  Visiting an attraction  Quiet activities  Playing with children  Appreciating nature  Eating or drinking  Socialising  Appreciating nature  Watersports/boating  Watersports/boating  Sunbathing/paddling  Swimming  Watersports/boating  Watersports/boating  Winter activities  Winter activities  Winter activities  Other |
| Companions on visit | How many adults aged 16 and over, including yourself, were on this visit?  How many children aged under 16 were on this visit? | 1 to 9  10 or more  1 to 9  10 or more | Adults and children (where there were >=1 adults and >1 children)  Other adults only (>=1 adults and 0 children)  Other children only (0 adults and >=1 children)  Alone (0 adults and 0 children; ref) |
| **Well-being outcomes** | | | |
| Happiness  Anxious  Worthwhile  Satisfied | How much do you agree with the statements below about your visit?  It made me feel happy  It made me feel anxious  I found the visit worthwhile  I was satisfied with the visit | Strongly disagree  Disagree  Slightly disagree  Neither agree nor disagree  Slightly agree  Agree  Strongly agree | -3  -2  -1  0  1  2  3  (numeric) |
| **Covariates** | |  |  |
| **Visitor characteristics** | |  |  |
| Age | (coded from panel registration information in most cases) | 18-29 (ref)  30-39  40-49  50-59  60+ |  |
| Sex | coded from panel registration information | Female (ref)  Male |  |
| Perceived financial strain | Which of these descriptions comes closest to how you feel about your household’s income nowadays? | Finding it very difficult on present income  Finding it difficult on present income  Coping on present income (ref)  Living comfortably on present income  Do not know |  |
|  |  |  |  |
| Limited by a long term limiting illness | Are you hampered in your daily activities in any way by any longstanding illness, or disability, infirmity or mental health problem? | No (ref)  Yes to some extent  Yes a lot |  |
| Garden access | Which of the following best applies to you? | I don’t have access to a private garden or outdoor space  I have access to a private communal garden  I have access to a private outdoor space, but not a garden (balcony, yard, patio area)  I have access to a private garden | No access (ref)  Communal garden  Private outdoor space (not a garden)  Private garden |
| Employment status | Which of these descriptions best describes your situation (in the last seven days)? Please select only one. | In paid work (or away temporarily) (employee, self-employed, working for your family business)  Unemployed and actively looking for a job  Unemployed, wanting a job but not actively looking for a job  In education, (not paid for by employer) even if on vacation  Doing housework, looking after children, or other persons  Retired  Permanently sick or disabled  In community or military service  Other  Do not know | Employed (ref)  Unemployed  Unemployed  In education  Housework  Retired  Disabled  Other/unsure  Other/unsure  Other/unsure |
| Educational attainment | Which of the following best describes your highest educational achievement? | Did not complete primary education  Completed primary education  Completed secondary/further education (up to 18 years of age)  Completed higher education (e.g. university degree or higher) | Primary  Primary  Secondary  University (ref) |
| Perceived minority ethnic group member | Do you belong to a minority ethnic group in the [COUNTRY]? “Belong” refers to attachment or identification | No (ref)  Yes  Do not know |  |
| Marital status | Which of the following best describes your marital status now? | Married, in a civil union, or living with your partner (cohabiting)  Single, separated/divorced/ civil union dissolved or widowed/civil partner died  Neither of these  Prefer not to answer | With partner  Not with partner (ref)  Missing  Missing |
| Dog ownership | Do you have a dog? | No (ref)  Yes |  |
| Country |  | Australia  Bulgaria  California  Canada  Czech Republic  Estonia  Finland  France  Germany  Greece  Hong Kong  Ireland  Italy  Netherlands  Portugal  Spain  Sweden  United Kingdom (ref) |  |
| Survey wave |  | Mar-18  Dec-17  Sep-17  Jun-17 (ref) |  |
|  |  |  |  |
| **Underlying well-being** | |  |  |
| World Health Organisation 5-item Wellbeing Index (WHO-5) | Please indicate for each of the five statements which is closest to how you have been feeling over the last two weeks.  “I have felt cheerful and in good spirits”  “I have felt calm and relaxed”  “I have felt active and vigorous”  “I woke up feeling fresh and rested”  “My daily life has been filled with things that interest me” | At no time = 0  Some of the time = 1  <Half the time = 2  >Half the time = 3  Most of the time = 4  All of the time = 5 | Values for all five variables added together and multiplied by 4. Range of values 0 - 100 |
| **Travel characteristics** | |  |  |
| Origin | Where did your journey start from? | Your home  Holiday accommodation  Elsewhere  Your place of work |  |
| Travel mode | What form of transport did you use on this journey for the majority of the distance? | Personal motorised transport (e.g. car, van, motorbike)  Walking (including wheelchair use and mobility scooters)  Bicycle  Ran/jogged  Bus  Train  Taxi  Hire car  Ferry or other public boat  Other (e.g. horseback) | Private vehicle  On foot/bike (ref)  On foot/bike (ref)  On foot/bike (ref)  Public transport  Public transport  Private vehicle  Private vehicle  Public transport  Other |
| Travel time | Approximately how long was your total journey time from your start point to the blue space you visited? | Free numeric response box with boxes for hours and minutes separately | <15 mins (ref)  15 - <30 mins  30 - <60 mins  60 - <120 mins  >=120 mins |

Table S2.

Counts for each variable for both the full sample, and the analytical samples used for the fully adjusted (Step 3) regressions for all four well-being outcome variables (smaller due to missing data)

|  | Full sample | | Happy | | Anxious | | Worthwhile | | Satisfied | |
| --- | --- | --- | --- | --- | --- | --- | --- | --- | --- | --- |
| Response | Counts | Weighted %age | Counts | %age | Counts | %age | Counts | %age | Counts | %age |
| **RQ1 Natural and environmental features** | | | | | | | | | | |
| 1a. Bluespace type | | | | | | | | | | |
| *Seaside promenade* | 2,528 | 17.01 | 2,505 | 16.82 | 2,505 | 16.82 | 2,504 | 16.81 | 2,504 | 16.81 |
| *Natural or artificial lake or reservoir* | 1,940 | 13.04 | 1,937 | 13.00 | 1,937 | 13.01 | 1,937 | 13.01 | 1,937 | 13.00 |
| *Urban river/canal (surrounded by buildings)* | 1,907 | 12.78 | 1,901 | 12.76 | 1,901 | 12.76 | 1,901 | 12.76 | 1,901 | 12.76 |
| *Sandy beach or dunes* | 1,554 | 10.14 | 1,547 | 10.39 | 1,547 | 10.39 | 1,547 | 10.39 | 1,547 | 10.39 |
| *Small water bodies (e.g. streams and ponds)* | 1,482 | 9.79 | 1,476 | 9.91 | 1,476 | 9.91 | 1,476 | 9.91 | 1,476 | 9.91 |
| *Rural river/canal (with vegetated banks)* | 1,447 | 9.56 | 1,445 | 9.70 | 1,445 | 9.70 | 1,445 | 9.70 | 1,445 | 9.70 |
| *Harbour or marina* | 804 | 5.40 | 800 | 5.37 | 800 | 5.37 | 800 | 5.37 | 800 | 5.37 |
| *Ornamental water feature or fountain* | 743 | 4.96 | 732 | 4.91 | 732 | 4.91 | 732 | 4.91 | 732 | 4.91 |
| *Outdoor public pool, lido, or thermal spa* | 580 | 3.94 | 573 | 3.85 | 572 | 3.84 | 573 | 3.85 | 573 | 3.85 |
| *Pier* | 425 | 2.84 | 413 | 2.77 | 413 | 2.77 | 412 | 2.77 | 413 | 2.77 |
| *Open sea* | 412 | 2.74 | 404 | 2.71 | 404 | 2.71 | 405 | 2.72 | 405 | 2.72 |
| *Waterfall or rapids* | 261 | 1.69 | 257 | 1.73 | 257 | 1.73 | 257 | 1.73 | 257 | 1.73 |
| *Outdoor skating or ice hockey rink* | 252 | 1.70 | 250 | 1.68 | 250 | 1.68 | 250 | 1.68 | 250 | 1.68 |
| *Rocky or stony shore* | 244 | 1.61 | 243 | 1.63 | 243 | 1.63 | 243 | 1.63 | 243 | 1.63 |
| *Fen, marsh or bog* | 190 | 1.25 | 189 | 1.27 | 189 | 1.27 | 189 | 1.27 | 189 | 1.27 |
| *Sea cliffs* | 155 | 1.07 | 155 | 1.04 | 155 | 1.04 | 155 | 1.04 | 155 | 1.04 |
| *Salt marsh, estuary or lagoon* | 68 | 0.48 | 68 | 0.46 | 68 | 0.46 | 68 | 0.46 | 68 | 0.46 |
| **1b Bluespace qualities** | | | | | | | |  |  |  |
| Perceived safety | |  |  |  |  |  |  |  |  |  |
| *-3 (strongly disagree)* | 126 | 0.85 | 126 | 0.85 | 126 | 0.85 | 126 | 0.85 | 126 | 0.85 |
| *-2* | 214 | 1.51 | 214 | 1.44 | 214 | 1.44 | 214 | 1.44 | 214 | 1.44 |
| *-1* | 446 | 3.00 | 443 | 2.97 | 443 | 2.97 | 443 | 2.97 | 443 | 2.97 |
| *0* | 2,667 | 17.56 | 2,642 | 17.74 | 2,643 | 17.75 | 2,643 | 17.75 | 2,643 | 17.74 |
| *1* | 3,241 | 21.74 | 3,206 | 21.52 | 3,206 | 21.53 | 3,206 | 21.53 | 3,206 | 21.52 |
| *2* | 5,263 | 34.97 | 5,241 | 35.19 | 5,239 | 35.18 | 5,239 | 35.18 | 5,240 | 35.18 |
| *3 (strongly agree)* | 3,028 | 20.36 | 3,023 | 20.30 | 3,023 | 20.30 | 3,023 | 20.30 | 3,023 | 20.30 |
| Perceived presence of wildlife | | |  |  |  |  |  |  |  |  |
| *-3 (strongly disagree)* | 1,383 | 9.66 | 1,373 | 9.22 | 1,372 | 9.21 | 1,373 | 9.22 | 1,373 | 9.22 |
| *-2* | 1,634 | 11.00 | 1,625 | 10.91 | 1,624 | 10.90 | 1,624 | 10.90 | 1,625 | 10.91 |
| *-1* | 1,168 | 7.77 | 1,149 | 7.71 | 1,149 | 7.71 | 1,149 | 7.71 | 1,149 | 7.71 |
| *0* | 2,155 | 14.47 | 2,116 | 14.21 | 2,117 | 14.21 | 2,117 | 14.21 | 2,116 | 14.21 |
| *1* | 3,225 | 21.04 | 3,214 | 21.58 | 3,214 | 21.58 | 3,214 | 21.58 | 3,214 | 21.58 |
| *2* | 3,368 | 22.39 | 3,358 | 22.54 | 3,358 | 22.55 | 3,357 | 22.54 | 3,358 | 22.54 |
| *3 (strongly agree)* | 2,060 | 13.66 | 2,060 | 13.83 | 2,060 | 13.83 | 2,060 | 13.83 | 2,060 | 13.83 |
| Perceived absence of litter | | | |  |  |  |  |  |  |  |
| *-3 (strongly disagree)* | 433 | 2.84 | 431 | 2.89 | 431 | 2.89 | 431 | 2.89 | 431 | 2.89 |
| *-2* | 886 | 5.94 | 879 | 5.90 | 879 | 5.90 | 878 | 5.89 | 879 | 5.90 |
| *-1* | 1,643 | 11.13 | 1,623 | 10.90 | 1,622 | 10.89 | 1,623 | 10.90 | 1,623 | 10.90 |
| *0* | 1,914 | 12.65 | 1,887 | 12.67 | 1,887 | 12.67 | 1,886 | 12.66 | 1,887 | 12.67 |
| *1* | 3,272 | 21.68 | 3,245 | 21.79 | 3,245 | 21.79 | 3,246 | 21.79 | 3,246 | 21.79 |
| *2* | 4,446 | 29.37 | 4,436 | 29.78 | 4,436 | 29.78 | 4,436 | 29.78 | 4,435 | 29.78 |
| *3 (strongly agree)* | 2,397 | 16.40 | 2,394 | 16.07 | 2,394 | 16.07 | 2,394 | 16.07 | 2,394 | 16.07 |
| Perceived presence of good facilities | | | | |  |  |  |  |  |  |
| *-3 (strongly disagree)* | 847 | 5.69 | 847 | 5.69 | 847 | 5.69 | 847 | 5.69 | 847 | 5.69 |
| *-2* | 1,159 | 7.80 | 1,154 | 7.75 | 1,154 | 7.75 | 1,153 | 7.74 | 1,154 | 7.75 |
| *-1* | 1,375 | 9.20 | 1,365 | 9.16 | 1,365 | 9.16 | 1,365 | 9.16 | 1,365 | 9.16 |
| *0* | 2,838 | 18.80 | 2,807 | 18.85 | 2,808 | 18.85 | 2,808 | 18.85 | 2,808 | 18.85 |
| *1* | 3,272 | 21.73 | 3,245 | 21.79 | 3,245 | 21.79 | 3,245 | 21.79 | 3,245 | 21.79 |
| *2* | 3,650 | 24.23 | 3,633 | 24.39 | 3,631 | 24.38 | 3,632 | 24.39 | 3,632 | 24.38 |
| *3 (strongly agree)* | 1,852 | 12.55 | 1,844 | 12.38 | 1,844 | 12.38 | 1,844 | 12.38 | 1,844 | 12.38 |
| Perceived water quality | | |  |  |  |  |  |  |  |  |
| *Poor* | 1,079 | 7.13 | 1,062 | 7.13 | 1,061 | 7.12 | 1,062 | 7.13 | 1,062 | 7.13 |
| *Sufficient* | 3,371 | 22.52 | 3,344 | 22.45 | 3,344 | 22.45 | 3,344 | 22.45 | 3,343 | 22.44 |
| *Good* | 6,998 | 46.57 | 6,948 | 46.65 | 6,948 | 46.65 | 6,947 | 46.64 | 6,949 | 46.65 |
| *Excellent* | 3,547 | 23.78 | 3,541 | 23.77 | 3,541 | 23.77 | 3,541 | 23.77 | 3,541 | 23.77 |
| **RQ2 Exposure** | | |  |  |  |  |  |  |  |  |
| Visit duration |  |  |  |  |  |  |  |  |  |  |
| *<30 mins* | 2,892 | 19.23 | 2,870 | 19.27 | 2,870 | 19.27 | 2,870 | 19.27 | 2,869 | 19.26 |
| *30 mins - <1 hr* | 2,987 | 20.18 | 2,956 | 19.85 | 2,955 | 19.84 | 2,954 | 19.83 | 2,956 | 19.85 |
| *1 - <1.5 hr* | 3,011 | 20.07 | 2,992 | 20.09 | 2,992 | 20.09 | 2,992 | 20.09 | 2,992 | 20.09 |
| *1.5 - <2 hr* | 1,178 | 7.81 | 1,172 | 7.87 | 1,172 | 7.87 | 1,172 | 7.87 | 1,172 | 7.87 |
| *2 - <3 hr* | 2,481 | 16.47 | 2,473 | 16.60 | 2,472 | 16.60 | 2,473 | 16.60 | 2,473 | 16.60 |
| *>=3 hr* | 2,442 | 16.24 | 2,432 | 16.33 | 2,433 | 16.34 | 2,433 | 16.34 | 2,433 | 16.33 |
| **RQ3. Experience** | |  |  |  |  |  |  |  |  |  |
| 3a. Activity |  |  |  |  |  |  |  |  |  |  |
| *Walking without a dog* | 4,540 | 30.31 | 4,497 | 30.19 | 4,497 | 30.19 | 4,496 | 30.19 | 4,496 | 30.18 |
| *Walking with a dog* | 1,628 | 10.52 | 1,626 | 10.92 | 1,626 | 10.92 | 1,626 | 10.92 | 1,626 | 10.92 |
| *Socialising* | 1,102 | 7.32 | 1,091 | 7.32 | 1,091 | 7.33 | 1,090 | 7.32 | 1,091 | 7.32 |
| *Appreciating nature* | 884 | 5.98 | 884 | 5.93 | 884 | 5.94 | 884 | 5.94 | 884 | 5.93 |
| *Swimming* | 878 | 5.92 | 872 | 5.85 | 871 | 5.85 | 872 | 5.85 | 872 | 5.85 |
| *Other* | 830 | 5.84 | 823 | 5.53 | 823 | 5.53 | 823 | 5.53 | 823 | 5.53 |
| *Playing with children* | 794 | 5.08 | 789 | 5.30 | 789 | 5.30 | 789 | 5.30 | 789 | 5.30 |
| *Running/nordic walking* | 625 | 4.09 | 619 | 4.16 | 619 | 4.16 | 619 | 4.16 | 619 | 4.16 |
| *Sunbathing/paddling* | 592 | 4.04 | 589 | 3.95 | 589 | 3.95 | 589 | 3.95 | 589 | 3.95 |
| *Eating or drinking* | 591 | 3.76 | 588 | 3.95 | 589 | 3.95 | 589 | 3.95 | 589 | 3.95 |
| *Quiet activities* | 472 | 3.20 | 470 | 3.16 | 470 | 3.16 | 470 | 3.16 | 470 | 3.16 |
| *Winter activities* | 455 | 3.06 | 454 | 3.05 | 454 | 3.05 | 454 | 3.05 | 454 | 3.05 |
| *Cycling* | 447 | 3.00 | 445 | 2.99 | 445 | 2.99 | 445 | 2.99 | 445 | 2.99 |
| *Watersports/boating* | 399 | 2.70 | 398 | 2.67 | 398 | 2.67 | 398 | 2.67 | 398 | 2.67 |
| *Visiting an attraction* | 339 | 2.35 | 332 | 2.23 | 331 | 2.22 | 332 | 2.23 | 332 | 2.23 |
| *Fishing* | 222 | 1.51 | 220 | 1.48 | 220 | 1.48 | 220 | 1.48 | 220 | 1.48 |
| *Sport* | 156 | 1.02 | 156 | 1.05 | 156 | 1.05 | 156 | 1.05 | 156 | 1.05 |
| *Conservation* | 42 | 0.28 | 42 | 0.28 | 42 | 0.28 | 42 | 0.28 | 42 | 0.28 |
| 3b. Visit companions |  |  |  |  |  |  |  |  |  |  |
| *Alone* | 5,269 | 35.46 | 5,239 | 35.17 | 5,238 | 35.17 | 5,239 | 35.18 | 5,238 | 35.17 |
| *Other adults only* | 5,562 | 37.35 | 5,546 | 37.23 | 5,546 | 37.24 | 5,545 | 37.23 | 5,547 | 37.24 |
| *Other children only* | 967 | 6.22 | 958 | 6.43 | 958 | 6.43 | 958 | 6.43 | 958 | 6.43 |
| *Adults and children* | 3,165 | 20.98 | 3,152 | 21.16 | 3,152 | 21.16 | 3,152 | 21.16 | 3,152 | 21.16 |
| **Well-being outcomes** |  |  |  |  |  |  |  |  |  |  |
| Visit happiness | |  |  |  |  |  |  |  |  |  |
| *-3 (strongly disagree)* | 94 | 0.61 | 93 | 0.62 | 93 | 0.62 | 93 | 0.62 | 93 | 0.62 |
| *- 2* | 105 | 0.70 | 104 | 0.70 | 104 | 0.70 | 104 | 0.70 | 104 | 0.70 |
| *-1* | 230 | 1.50 | 225 | 1.51 | 225 | 1.51 | 225 | 1.51 | 225 | 1.51 |
| *0* | 1,348 | 9.09 | 1,331 | 8.94 | 1,331 | 8.94 | 1,331 | 8.94 | 1,331 | 8.94 |
| *1* | 3,016 | 20.10 | 2,976 | 19.98 | 2,976 | 19.98 | 2,975 | 19.98 | 2,976 | 19.98 |
| *2* | 5,769 | 38.06 | 5,742 | 38.55 | 5,740 | 38.54 | 5,742 | 38.56 | 5,741 | 38.55 |
| *3 (strongly agree)* | 4,430 | 29.93 | 4,424 | 29.70 | 4,424 | 29.71 | 4,423 | 29.70 | 4,424 | 29.70 |
| Visit anxiety | |  |  |  |  |  |  |  |  |  |
| *-3 (strongly disagree)* | 6,582 | 44.19 | 6,560 | 44.05 | 6,560 | 44.04 | 6,559 | 44.04 | 6,559 | 44.04 |
| *-2* | 4,343 | 28.74 | 4,317 | 28.99 | 4,317 | 28.98 | 4,317 | 28.99 | 4,317 | 28.99 |
| *-1* | 1,470 | 9.76 | 1,446 | 9.71 | 1,446 | 9.71 | 1,445 | 9.70 | 1,446 | 9.71 |
| *0* | 1,294 | 8.66 | 1,277 | 8.57 | 1,277 | 8.57 | 1,277 | 8.58 | 1,277 | 8.57 |
| *1* | 637 | 4.19 | 631 | 4.24 | 632 | 4.24 | 632 | 4.24 | 632 | 4.24 |
| *2* | 453 | 3.05 | 450 | 3.02 | 450 | 3.02 | 450 | 3.02 | 450 | 3.02 |
| *3 (strongly agree)* | 212 | 1.41 | 212 | 1.42 | 212 | 1.42 | 212 | 1.42 | 212 | 1.42 |
| Visit worthwhile | | |  |  |  |  |  |  |  |  |
| *-3 (strongly disagree)* | 73 | 0.48 | 72 | 0.48 | 72 | 0.48 | 72 | 0.48 | 72 | 0.48 |
| *-2* | 96 | 0.66 | 93 | 0.62 | 93 | 0.62 | 93 | 0.62 | 93 | 0.62 |
| *-1* | 226 | 1.48 | 224 | 1.50 | 224 | 1.50 | 224 | 1.50 | 224 | 1.50 |
| *0* | 1,472 | 9.79 | 1,449 | 9.73 | 1,449 | 9.73 | 1,449 | 9.73 | 1,449 | 9.73 |
| *1* | 2,727 | 18.21 | 2,698 | 18.12 | 2,698 | 18.12 | 2,698 | 18.11 | 2,698 | 18.12 |
| *2* | 5,720 | 37.88 | 5,690 | 38.21 | 5,689 | 38.20 | 5,691 | 38.21 | 5,690 | 38.21 |
| *3 (strongly agree)* | 4,676 | 31.50 | 4,667 | 31.34 | 4,667 | 31.34 | 4,667 | 31.33 | 4,667 | 31.34 |
| Visit satisfaction | | |  |  |  |  |  |  |  |  |
| *-3 (strongly disagree)* | 45 | 0.28 | 44 | 0.30 | 44 | 0.30 | 44 | 0.30 | 44 | 0.30 |
| *-2* | 84 | 0.58 | 81 | 0.54 | 81 | 0.54 | 81 | 0.54 | 81 | 0.54 |
| *-1* | 168 | 1.10 | 165 | 1.11 | 165 | 1.11 | 165 | 1.11 | 165 | 1.11 |
| *0* | 1,106 | 7.36 | 1,087 | 7.30 | 1,087 | 7.30 | 1,087 | 7.30 | 1,087 | 7.30 |
| *1* | 2,806 | 18.67 | 2,771 | 18.60 | 2,771 | 18.61 | 2,771 | 18.61 | 2,771 | 18.60 |
| *2* | 6,127 | 40.59 | 6,095 | 40.92 | 6,094 | 40.92 | 6,094 | 40.92 | 6,096 | 40.93 |
| *3 (strongly agree)* | 4,655 | 31.42 | 4,651 | 31.23 | 4,651 | 31.23 | 4,651 | 31.23 | 4,651 | 31.23 |
| **Visitor characteristics** | | |  |  |  |  |  |  |  |  |
| Age | | |  |  |  |  |  |  |  |  |
| *18-29* | 3,509 | 18.55 | 2,920 | 19.60 | 2,920 | 19.61 | 2,920 | 19.61 | 2,921 | 19.61 |
| *30-39* | 3,411 | 16.98 | 2,765 | 18.56 | 2,765 | 18.56 | 2,765 | 18.56 | 2,765 | 18.56 |
| *40-49* | 3,563 | 17.92 | 2,742 | 18.41 | 2,742 | 18.41 | 2,742 | 18.41 | 2,741 | 18.40 |
| *50-59* | 3,398 | 16.84 | 2,612 | 17.54 | 2,611 | 17.53 | 2,611 | 17.53 | 2,612 | 17.54 |
| *60+* | 4,957 | 29.70 | 3,856 | 25.89 | 3,856 | 25.89 | 3,856 | 25.89 | 3,856 | 25.89 |
| Sex |  |  |  |  |  |  |  |  |  |  |
| *Female* | 9,645 | 51.79 | 7,428 | 49.87 | 7,428 | 49.87 | 7,429 | 49.88 | 7,428 | 49.87 |
| *Male* | 9,193 | 48.21 | 7,467 | 50.13 | 7,466 | 50.13 | 7,465 | 50.12 | 7,467 | 50.13 |
| Perceived financial strain | | | |  |  |  |  |  |  |  |
| *Finding it very difficult on present income* | 1,219 | 6.78 | 837 | 5.62 | 837 | 5.62 | 837 | 5.62 | 837 | 5.62 |
| *Finding it difficult on present income* | 3,329 | 17.87 | 2,574 | 17.28 | 2,574 | 17.28 | 2,574 | 17.28 | 2,574 | 17.28 |
| *Coping on present income* | 8,758 | 46.24 | 6,970 | 46.79 | 6,970 | 46.80 | 6,970 | 46.80 | 6,971 | 46.80 |
| *Living comfortably on present income* | 5,264 | 27.64 | 4,330 | 29.07 | 4,329 | 29.07 | 4,329 | 29.07 | 4,329 | 29.06 |
| *Missing* | 268 | 1.47 | 184 | 1.24 | 184 | 1.24 | 184 | 1.24 | 184 | 1.24 |
| Presence of limiting illness/disability | | | | | |  |  |  |  |  |
| *No* | 11,885 | 63.10 | 9,603 | 64.47 | 9,602 | 64.47 | 9,602 | 64.47 | 9,603 | 64.47 |
| *Yes to some extent* | 5,210 | 27.53 | 3,983 | 26.74 | 3,983 | 26.74 | 3,983 | 26.74 | 3,983 | 26.74 |
| *Yes a lot* | 1,739 | 9.36 | 1,307 | 8.77 | 1,307 | 8.78 | 1,307 | 8.78 | 1,307 | 8.77 |
| *Missing (excluded)* | 4 | 0.02 | 2 | 0.01 | 2 | 0.01 | 2 | 0.01 | 2 | 0.01 |
| Garden access | |  |  |  |  |  |  |  |  |  |
| *Missing (excluded)* | 1 | 0.01 | 1 | 0.01 | 1 | 0.01 | 1 | 0.01 | 1 | 0.01 |
| *No access* | 3,884 | 20.57 | 2,825 | 18.97 | 2,825 | 18.97 | 2,826 | 18.97 | 2,825 | 18.97 |
| *Communal Garden* | 2,077 | 11.18 | 1,814 | 12.18 | 1,814 | 12.18 | 1,813 | 12.17 | 1,814 | 12.18 |
| *Private outdoor space (not a garden)* | 3,732 | 20.01 | 3,004 | 20.17 | 3,003 | 20.16 | 3,004 | 20.17 | 3,004 | 20.17 |
| *Private garden* | 9,144 | 48.24 | 7,251 | 48.68 | 7,251 | 48.68 | 7,250 | 48.68 | 7,251 | 48.68 |
| Employment status | | | |  |  |  |  |  |  |  |
| *Employed* | 10,392 | 54.02 | 8,346 | 56.03 | 8,345 | 56.03 | 8,345 | 56.03 | 8,347 | 56.04 |
| *In education* | 1,202 | 6.29 | 1,012 | 6.79 | 1,012 | 6.79 | 1,012 | 6.79 | 1,012 | 6.79 |
| *Housework* | 1,005 | 5.40 | 753 | 5.06 | 753 | 5.06 | 753 | 5.06 | 752 | 5.05 |
| *Retired* | 3,564 | 20.28 | 2,793 | 18.75 | 2,793 | 18.75 | 2,793 | 18.75 | 2,793 | 18.75 |
| *Disabled* | 664 | 3.45 | 434 | 2.91 | 434 | 2.91 | 434 | 2.91 | 434 | 2.91 |
| *Unemployed* | 1,221 | 6.47 | 942 | 6.32 | 942 | 6.32 | 942 | 6.32 | 942 | 6.32 |
| *Other/unsure* | 786 | 4.09 | 615 | 4.13 | 615 | 4.13 | 615 | 4.13 | 615 | 4.13 |
| Educational attainment | | | |  |  |  |  |  |  |  |
| *University* | 9,594 | 50.16 | 7,881 | 52.91 | 7,880 | 52.91 | 7,880 | 52.91 | 7,881 | 52.91 |
| *Secondary* | 7,582 | 40.52 | 5,836 | 39.18 | 5,836 | 39.18 | 5,836 | 39.18 | 5,836 | 39.18 |
| *Primary* | 1,660 | 9.32 | 1,178 | 7.91 | 1,178 | 7.91 | 1,178 | 7.91 | 1,178 | 7.91 |
| Perceived member of ethnic minority group | | | | |  |  |  |  |  |  |
| *No* | 16,841 | 89.41 | 13,352 | 89.64 | 13,351 | 89.64 | 13,351 | 89.64 | 13,352 | 89.64 |
| *Yes* | 1,331 | 7.02 | 1,045 | 7.02 | 1,045 | 7.02 | 1,045 | 7.02 | 1,045 | 7.02 |
| *Do not know* | 666 | 3.58 | 498 | 3.34 | 498 | 3.34 | 498 | 3.34 | 498 | 3.34 |
| Relationship status | | | |  |  |  |  |  |  |  |
| *Missing* | 861 | 4.56 | 703 | 4.72 | 702 | 4.71 | 703 | 4.72 | 703 | 4.72 |
| *Not married* | 6,866 | 36.83 | 5,179 | 34.77 | 5,180 | 34.78 | 5,180 | 34.78 | 5,180 | 34.78 |
| *Married/cohabiting* | 11,110 | 58.61 | 9,013 | 60.51 | 9,012 | 60.51 | 9,011 | 60.50 | 9,012 | 60.50 |
| Dog ownership | | |  |  |  |  |  |  |  |  |
| *No* | 13,179 | 70.15 | 10,206 | 68.52 | 10,206 | 68.52 | 10,205 | 68.52 | 10,206 | 68.52 |
| *Yes* | 5,656 | 29.85 | 4,689 | 31.48 | 4,688 | 31.48 | 4,689 | 31.48 | 4,689 | 31.48 |
| Country of residence | | | |  |  |  |  |  |  |  |
| *Australia* | 1,001 | 5.33 | 785 | 5.27 | 785 | 5.27 | 785 | 5.27 | 785 | 5.27 |
| *Bulgaria* | 1,054 | 5.61 | 929 | 6.24 | 929 | 6.24 | 929 | 6.24 | 929 | 6.24 |
| *California* | 1,078 | 5.74 | 737 | 4.95 | 737 | 4.95 | 737 | 4.95 | 737 | 4.95 |
| *Canada* | 1,030 | 5.48 | 690 | 4.63 | 690 | 4.63 | 690 | 4.63 | 690 | 4.63 |
| *Czech Republic* | 1,080 | 5.75 | 857 | 5.75 | 857 | 5.75 | 857 | 5.75 | 857 | 5.75 |
| *Estonia* | 961 | 5.08 | 793 | 5.32 | 793 | 5.32 | 793 | 5.32 | 793 | 5.32 |
| *Finland* | 1,061 | 5.65 | 863 | 5.79 | 863 | 5.79 | 863 | 5.79 | 863 | 5.79 |
| *France* | 1,071 | 5.70 | 780 | 5.24 | 780 | 5.24 | 780 | 5.24 | 780 | 5.24 |
| *Germany* | 1,025 | 5.46 | 770 | 5.17 | 770 | 5.17 | 770 | 5.17 | 770 | 5.17 |
| *Greece* | 970 | 5.16 | 901 | 6.05 | 901 | 6.05 | 901 | 6.05 | 901 | 6.05 |
| *Hong Kong* | 984 | 5.23 | 715 | 4.80 | 714 | 4.79 | 714 | 4.79 | 715 | 4.80 |
| *Ireland* | 1,059 | 5.64 | 856 | 5.75 | 856 | 5.75 | 856 | 5.75 | 856 | 5.75 |
| *Italy* | 1,066 | 5.67 | 896 | 6.02 | 896 | 6.02 | 896 | 6.02 | 896 | 6.02 |
| *Netherlands* | 1,062 | 5.65 | 779 | 5.23 | 779 | 5.23 | 779 | 5.23 | 779 | 5.23 |
| *Portugal* | 946 | 5.01 | 823 | 5.53 | 823 | 5.53 | 823 | 5.53 | 823 | 5.53 |
| *Spain* | 1,054 | 5.61 | 954 | 6.40 | 954 | 6.41 | 954 | 6.41 | 954 | 6.40 |
| *Sweden* | 1,069 | 5.69 | 839 | 5.63 | 839 | 5.63 | 839 | 5.63 | 839 | 5.63 |
| *United Kingdom* | 1,267 | 6.54 | 928 | 6.23 | 928 | 6.23 | 928 | 6.23 | 928 | 6.23 |
| Survey wave | | |  |  |  |  |  |  |  |  |
| *Jun-17* | 4,728 | 25.08 | 3,910 | 26.25 | 3,909 | 26.25 | 3,910 | 26.25 | 3,910 | 26.25 |
| *Sep-17* | 4,544 | 24.11 | 3,792 | 25.46 | 3,793 | 25.47 | 3,792 | 25.46 | 3,792 | 25.46 |
| *Dec-17* | 4,716 | 25.05 | 3,554 | 23.86 | 3,554 | 23.86 | 3,554 | 23.86 | 3,554 | 23.86 |
| *Mar-18* | 4,850 | 25.76 | 3,639 | 24.43 | 3,638 | 24.43 | 3,638 | 24.43 | 3,639 | 24.43 |
| **Travel characteristics** | | |  |  |  |  |  |  |  |  |
| Travel origin |  |  |  |  |  |  |  |  |  |  |
| *Your home* | 12,311 | 82.15 | 12,234 | 82.13 | 12,233 | 82.13 | 12,232 | 82.13 | 12,233 | 82.13 |
| *Your place of work* | 646 | 4.19 | 638 | 4.28 | 638 | 4.28 | 638 | 4.28 | 638 | 4.28 |
| *Holiday accommodation* | 1,106 | 7.44 | 1,100 | 7.39 | 1,099 | 7.38 | 1,100 | 7.39 | 1,100 | 7.39 |
| *Elsewhere* | 931 | 6.22 | 923 | 6.20 | 924 | 6.20 | 924 | 6.20 | 924 | 6.20 |
| Travel mode | |  |  |  |  |  |  |  |  |  |
| *On foot/bike* | 5,540 | 36.90 | 5,496 | 36.90 | 5,496 | 36.90 | 5,494 | 36.89 | 5,495 | 36.89 |
| *Other (e.g. horseback)* | 268 | 1.89 | 265 | 1.78 | 265 | 1.78 | 266 | 1.79 | 266 | 1.79 |
| *Public transport* | 1,426 | 9.54 | 1,395 | 9.37 | 1,394 | 9.36 | 1,395 | 9.37 | 1,395 | 9.37 |
| *Private vehicle* | 7,753 | 51.67 | 7,739 | 51.96 | 7,739 | 51.96 | 7,739 | 51.96 | 7,739 | 51.96 |
| Travel time | |  |  |  |  |  |  |  |  |  |
| *<15 mins* | 3,730 | 24.80 | 3,711 | 24.91 | 3,711 | 24.92 | 3,710 | 24.91 | 3,710 | 24.91 |
| *15 - <30 mins* | 3,640 | 24.32 | 3,626 | 24.34 | 3,626 | 24.35 | 3,626 | 24.35 | 3,626 | 24.34 |
| *30 - <60 mins* | 2,671 | 17.79 | 2,658 | 17.84 | 2,658 | 17.85 | 2,657 | 17.84 | 2,658 | 17.84 |
| *60 - <120 mins* | 2,821 | 18.90 | 2,792 | 18.74 | 2,792 | 18.75 | 2,792 | 18.75 | 2,792 | 18.74 |
| *>=120 mins* | 2,129 | 14.18 | 2,108 | 14.15 | 2,107 | 14.15 | 2,109 | 14.16 | 2,109 | 14.16 |

Table S3

Mean and standard deviation (SD) for each outcome variable for the full sample and each variable. Sample counts for the full modelling sample are available in Table S2.

|  | Happy | | Anxious | | Worthwhile | | Satisfied | |
| --- | --- | --- | --- | --- | --- | --- | --- | --- |
| Response | Mean | SD | Mean | SD | Mean | SD | Mean | SD |
| **Full sample** | 1.81 | 1.11 | -1.85 | 1.4 | 1.84 | 1.10 | 1.91 | 1.02 |
| **RQ1. Natural and environmental features** | | | | |  |  |  |  |
| **1a. Bluespace type** | | | |  |  |  |  |  |
| *Seaside promenade* | 1.88 | 0.98 | -1.90 | 1.4 | 1.94 | 1.03 | 1.97 | 0.95 |
| *Lake/reservoir* | 1.89 | 1.04 | -1.95 | 1.5 | 1.91 | 1.04 | 2.02 | 0.94 |
| *Urban river/canal (ref)* | 1.54 | 1.16 | -1.74 | 1.5 | 1.53 | 1.17 | 1.64 | 1.08 |
| *Sandy beach or dunes* | 2.21 | 0.91 | -2.02 | 1.3 | 2.27 | 0.88 | 2.26 | 0.84 |
| *Small water bodies* | 1.70 | 1.16 | -1.97 | 1.3 | 1.67 | 1.15 | 1.79 | 1.06 |
| *Rural river/canal* | 1.85 | 1.07 | -1.93 | 1.4 | 1.87 | 1.06 | 1.96 | 0.96 |
| *Harbour or marina* | 1.84 | 1.08 | -1.90 | 1.4 | 1.92 | 1.04 | 1.98 | 0.97 |
| *Water feature/fountain* | 1.45 | 1.19 | -1.77 | 1.4 | 1.47 | 1.19 | 1.55 | 1.14 |
| *Outdoor pool/spa* | 1.73 | 1.20 | -1.51 | 1.6 | 1.77 | 1.09 | 1.82 | 1.04 |
| *Pier* | 1.65 | 1.16 | -1.61 | 1.5 | 1.64 | 1.13 | 1.73 | 1.08 |
| *Open sea* | 2.03 | 1.07 | -1.76 | 1.5 | 2.09 | 1.04 | 2.11 | 0.96 |
| *Waterfall or rapids* | 1.57 | 1.49 | -1.07 | 2.0 | 1.71 | 1.35 | 1.71 | 1.34 |
| *Ice rink* | 1.60 | 1.29 | -1.56 | 1.6 | 1.64 | 1.22 | 1.74 | 1.14 |
| *Rocky or stony shore* | 2.16 | 0.92 | -2.16 | 1.2 | 2.21 | 0.91 | 2.24 | 0.82 |
| *Fen, marsh or bog* | 1.51 | 1.45 | -1.20 | 1.8 | 1.53 | 1.38 | 1.60 | 1.37 |
| *Sea cliffs* | 2.12 | 1.03 | -2.04 | 1.3 | 2.15 | 0.97 | 2.23 | 0.84 |
| *Salt marsh, estuary or lagoon* | 1.97 | 0.98 | -2.06 | 1.4 | 2.07 | 1.19 | 2.01 | 1.01 |
| **1b. Bluespace qualities** | | | |  |  |  |  |  |
| Perceived safety | |  |  |  |  |  |  |  |
| *Strongly disagree* | -0.12 | 1.95 | -1.42 | 2.0 | 0.23 | 1.96 | 0.40 | 1.88 |
| *Disagree* | 0.55 | 1.63 | -1.35 | 1.5 | 0.65 | 1.61 | 0.72 | 1.58 |
| *Slightly disagree* | 0.84 | 1.39 | -1.20 | 1.3 | 0.92 | 1.30 | 0.90 | 1.33 |
| *Neither agree nor disagree* | 1.23 | 1.16 | -1.59 | 1.3 | 1.25 | 1.17 | 1.33 | 1.10 |
| *Slightly agree* | 1.55 | 0.94 | -1.58 | 1.4 | 1.54 | 0.96 | 1.63 | 0.83 |
| *Agree* | 2.02 | 0.86 | -1.97 | 1.4 | 2.06 | 0.84 | 2.11 | 0.74 |
| *Strongly agree* | 2.56 | 0.79 | -2.31 | 1.5 | 2.59 | 0.80 | 2.66 | 0.67 |
| Perceived presence of wildlife | | | |  |  |  |  |  |
| *Strongly disagree* | 1.53 | 1.37 | -2.20 | 1.3 | 1.67 | 1.32 | 1.78 | 1.22 |
| *Disagree* | 1.55 | 1.14 | -1.96 | 1.1 | 1.59 | 1.16 | 1.68 | 1.07 |
| *Slightly disagree* | 1.37 | 1.26 | -1.73 | 1.2 | 1.45 | 1.21 | 1.53 | 1.13 |
| *Neither agree nor disagree* | 1.45 | 1.15 | -1.56 | 1.4 | 1.43 | 1.17 | 1.50 | 1.10 |
| *Slightly agree* | 1.70 | 0.97 | -1.71 | 1.4 | 1.73 | 0.98 | 1.79 | 0.90 |
| *Agree* | 2.08 | 0.84 | -1.87 | 1.5 | 2.09 | 0.86 | 2.13 | 0.77 |
| *Strongly agree* | 2.59 | 0.80 | -2.09 | 1.7 | 2.59 | 0.81 | 2.65 | 0.71 |
| Perceived presence of litter | | |  |  |  |  |  |  |
| *Strongly disagree* | 1.36 | 1.60 | -1.80 | 1.6 | 1.41 | 1.63 | 1.39 | 1.59 |
| *Disagree* | 1.45 | 1.32 | -1.83 | 1.3 | 1.51 | 1.25 | 1.53 | 1.19 |
| *Slightly disagree* | 1.57 | 1.17 | -1.86 | 1.2 | 1.60 | 1.16 | 1.65 | 1.08 |
| *Neither agree nor disagree* | 1.30 | 1.17 | -1.48 | 1.3 | 1.31 | 1.13 | 1.37 | 1.10 |
| *Slightly agree* | 1.69 | 1.00 | -1.70 | 1.4 | 1.70 | 1.03 | 1.78 | 0.89 |
| *Agree* | 1.99 | 0.91 | -1.97 | 1.4 | 2.02 | 0.91 | 2.09 | 0.80 |
| *Strongly agree* | 2.45 | 0.90 | -2.16 | 1.6 | 2.50 | 0.88 | 2.58 | 0.77 |
| Perceived presence of good facilities | | | |  |  |  |  |  |
| *Strongly disagree* | 1.58 | 1.42 | -2.08 | 1.4 | 1.62 | 1.45 | 1.75 | 1.35 |
| *Disagree* | 1.57 | 1.26 | -1.96 | 1.2 | 1.56 | 1.26 | 1.62 | 1.19 |
| *Slightly disagree* | 1.54 | 1.21 | -1.87 | 1.2 | 1.54 | 1.17 | 1.61 | 1.08 |
| *Neither agree nor disagree* | 1.45 | 1.16 | -1.75 | 1.3 | 1.46 | 1.16 | 1.54 | 1.10 |
| *Slightly agree* | 1.74 | 0.96 | -1.71 | 1.4 | 1.76 | 0.98 | 1.83 | 0.88 |
| *Agree* | 2.04 | 0.87 | -1.87 | 1.5 | 2.09 | 0.83 | 2.14 | 0.75 |
| *Strongly agree* | 2.53 | 0.87 | -2.03 | 1.8 | 2.59 | 0.82 | 2.64 | 0.73 |
| Perceived water quality | | |  |  |  |  |  |  |
| *Poor* | 1.30 | 1.31 | -1.66 | 1.4 | 1.32 | 1.30 | 1.31 | 1.27 |
| *Sufficient (ref)* | 1.55 | 1.12 | -1.83 | 1.3 | 1.59 | 1.12 | 1.66 | 1.03 |
| *Good* | 1.79 | 1.04 | -1.85 | 1.4 | 1.81 | 1.04 | 1.89 | 0.95 |
| *Excellent* | 2.27 | 0.98 | -1.94 | 1.6 | 2.30 | 0.98 | 2.37 | 0.87 |
| **RQ2. Exposure – visit duration** | | | | |  |  |  |  |
| *<30 mins (ref)* | 1.29 | 1.20 | -1.87 | 1.3 | 1.28 | 1.22 | 1.43 | 1.13 |
| *30 mins - <1 hr* | 1.69 | 1.08 | -1.79 | 1.4 | 1.72 | 1.08 | 1.80 | 0.99 |
| *1 - <1.5 hr* | 1.87 | 1.02 | -1.81 | 1.5 | 1.89 | 0.98 | 1.95 | 0.92 |
| *1.5 - <2 hr* | 1.98 | 1.01 | -1.89 | 1.5 | 2.02 | 1.03 | 2.04 | 1.01 |
| *2 - <3 hr* | 2.05 | 1.04 | -1.83 | 1.5 | 2.09 | 1.00 | 2.11 | 0.95 |
| *>=3 hr* | 2.20 | 0.95 | -1.97 | 1.4 | 2.26 | 0.95 | 2.29 | 0.85 |
| **RQ3. Experience** | | |  |  |  |  |  |  |
| **3a. Visit activity** | |  |  |  |  |  |  |  |
| *Walking without a dog* | 1.72 | 1.06 | -2.01 | 1.3 | 1.75 | 1.10 | 1.83 | 1.00 |
| *Walking with a dog* | 1.93 | 1.07 | -1.87 | 1.6 | 1.94 | 1.01 | 2.02 | 0.95 |
| *Socialising* | 1.93 | 0.98 | -1.89 | 1.3 | 1.94 | 1.04 | 2.00 | 0.92 |
| *Appreciating nature* | 1.81 | 1.11 | -2.01 | 1.3 | 1.78 | 1.15 | 1.89 | 1.07 |
| *Swimming* | 2.06 | 1.08 | -1.84 | 1.4 | 2.13 | 1.01 | 2.13 | 0.98 |
| *Other* | 1.53 | 1.25 | -1.69 | 1.5 | 1.59 | 1.28 | 1.68 | 1.20 |
| *Playing with children* | 2.09 | 0.92 | -1.86 | 1.4 | 2.08 | 0.95 | 2.11 | 0.89 |
| *Running/nordic walking* | 1.54 | 1.32 | -1.13 | 1.9 | 1.61 | 1.20 | 1.63 | 1.17 |
| *Sunbathing/paddling* | 2.13 | 0.92 | -2.00 | 1.4 | 2.13 | 0.95 | 2.15 | 0.85 |
| *Eating or drinking* | 1.88 | 0.99 | -1.97 | 1.3 | 1.90 | 0.97 | 1.98 | 0.87 |
| *Quiet activities* | 1.93 | 1.02 | -2.07 | 1.2 | 1.97 | 1.00 | 2.00 | 0.94 |
| *Winter activities* | 1.65 | 1.31 | -1.36 | 1.7 | 1.67 | 1.23 | 1.86 | 1.11 |
| *Cycling* | 1.56 | 1.24 | -1.66 | 1.6 | 1.57 | 1.21 | 1.64 | 1.17 |
| *Watersports/boating* | 1.93 | 1.26 | -1.73 | 1.6 | 2.06 | 1.13 | 2.12 | 1.00 |
| *Visiting an attraction* | 1.71 | 1.05 | -1.74 | 1.4 | 1.75 | 1.11 | 1.85 | 1.05 |
| *Fishing* | 2.11 | 0.98 | -1.86 | 1.4 | 2.01 | 1.04 | 2.07 | 0.95 |
| *Sport* | 1.42 | 1.56 | -0.88 | 1.9 | 1.56 | 1.37 | 1.52 | 1.39 |
| *Conservation* | 1.07 | 1.69 | -0.95 | 1.9 | 1.64 | 1.30 | 1.52 | 1.45 |
| **3b. Visit companions** | | |  |  |  |  |  |  |
| *Alone* | 1.66 | 1.13 | -1.95 | 1.3 | 1.67 | 1.14 | 1.77 | 1.05 |
| *Other adults only* | 1.92 | 1.03 | -1.96 | 1.4 | 1.96 | 1.03 | 2.02 | 0.95 |
| *Other children only* | 1.85 | 1.07 | -1.78 | 1.5 | 1.83 | 1.09 | 1.88 | 1.04 |
| *Adults and children* | 1.87 | 1.17 | -1.53 | 1.7 | 1.92 | 1.13 | 1.96 | 1.06 |
| **Visitor characteristics** | | |  |  |  |  |  |  |
| Age |  |  |  |  |  |  |  |  |
| *18-29 (ref)* | 1.59 | 1.23 | -1.42 | 1.6 | 1.56 | 1.22 | 1.67 | 1.11 |
| *30-39* | 1.74 | 1.17 | -1.55 | 1.6 | 1.74 | 1.13 | 1.80 | 1.06 |
| *40-49* | 1.91 | 1.07 | -1.89 | 1.4 | 1.96 | 1.07 | 1.99 | 1.00 |
| *50-59* | 1.92 | 1.02 | -2.11 | 1.2 | 1.98 | 1.00 | 2.02 | 0.95 |
| *60+* | 1.90 | 1.01 | -2.19 | 1.2 | 1.96 | 1.03 | 2.04 | 0.93 |
| Gender |  |  |  |  |  |  |  |  |
| *Female* | 1.90 | 1.09 | -1.93 | 1.4 | 1.91 | 1.10 | 1.98 | 1.01 |
| *Male* | 1.73 | 1.12 | -1.78 | 1.5 | 1.78 | 1.10 | 1.85 | 1.03 |
| Perceived financial strain | | |  |  |  |  |  |  |
| *Finding it very difficult on present income* | 1.68 | 1.31 | -1.76 | 1.5 | 1.69 | 1.27 | 1.74 | 1.21 |
| *Finding it difficult on present income* | 1.75 | 1.15 | -1.79 | 1.4 | 1.80 | 1.15 | 1.84 | 1.07 |
| *Coping on present income* | 1.82 | 1.06 | -1.89 | 1.4 | 1.85 | 1.07 | 1.92 | 0.98 |
| *Living comfortably on present income* | 1.90 | 1.07 | -1.88 | 1.6 | 1.93 | 1.06 | 2.01 | 0.98 |
| *Missing* | 1.02 | 1.44 | -1.10 | 1.5 | 1.03 | 1.41 | 1.21 | 1.27 |
| Presence of limiting illness/disability | | | |  |  |  |  |  |
| *No* | 1.90 | 1.03 | -2.02 | 1.3 | 1.92 | 1.05 | 1.99 | 0.96 |
| *Yes to some extent* | 1.69 | 1.17 | -1.79 | 1.4 | 1.72 | 1.16 | 1.78 | 1.08 |
| *Yes a lot* | 1.57 | 1.33 | -0.80 | 2.0 | 1.62 | 1.26 | 1.71 | 1.17 |
| Garden access | |  |  |  |  |  |  |  |
| *No access* | 1.63 | 1.14 | -1.75 | 1.4 | 1.68 | 1.14 | 1.72 | 1.08 |
| *Communal Garden* | 1.71 | 1.18 | -1.43 | 1.7 | 1.78 | 1.16 | 1.82 | 1.10 |
| *Private outdoor space (not a garden)* | 1.79 | 1.13 | -1.91 | 1.4 | 1.83 | 1.12 | 1.90 | 1.04 |
| *Private garden* | 1.92 | 1.05 | -1.97 | 1.4 | 1.93 | 1.05 | 2.01 | 0.96 |
| Economic status | | | |  |  |  |  |  |
| *Employed* | 1.84 | 1.08 | -1.79 | 1.5 | 1.86 | 1.09 | 1.92 | 1.00 |
| *In education* | 1.58 | 1.18 | -1.63 | 1.5 | 1.58 | 1.17 | 1.68 | 1.08 |
| *Housework* | 1.93 | 1.07 | -1.79 | 1.4 | 1.93 | 1.13 | 1.97 | 1.03 |
| *Retired* | 1.89 | 1.02 | -2.19 | 1.2 | 1.95 | 1.02 | 2.04 | 0.94 |
| *Disabled* | 1.73 | 1.25 | -1.88 | 1.4 | 1.85 | 1.14 | 1.94 | 1.05 |
| *Unemployed* | 1.64 | 1.25 | -1.74 | 1.4 | 1.71 | 1.19 | 1.75 | 1.15 |
| *Other/unsure* | 1.69 | 1.25 | -1.78 | 1.4 | 1.67 | 1.26 | 1.72 | 1.23 |
| Educational attainment | | | |  |  |  |  |  |
| *University* | 1.85 | 1.06 | -1.86 | 1.4 | 1.89 | 1.07 | 1.93 | 0.99 |
| *Secondary* | 1.82 | 1.10 | -1.87 | 1.4 | 1.83 | 1.11 | 1.92 | 1.02 |
| *Primary* | 1.52 | 1.35 | -1.72 | 1.5 | 1.61 | 1.26 | 1.72 | 1.21 |
| Self-reported ethnic minority member | | | |  |  |  |  |  |
| *No* | 1.83 | 1.08 | -1.93 | 1.4 | 1.85 | 1.09 | 1.92 | 1.01 |
| *Yes* | 1.81 | 1.23 | -1.21 | 1.8 | 1.86 | 1.14 | 1.92 | 1.07 |
| *Do not know* | 1.45 | 1.41 | -1.14 | 1.7 | 1.52 | 1.35 | 1.59 | 1.26 |
| Marital status | |  |  |  |  |  |  |  |
| *Not married* | 1.71 | 1.17 | -1.83 | 1.4 | 1.74 | 1.15 | 1.81 | 1.07 |
| *Married/cohabiting* | 1.90 | 1.04 | -1.89 | 1.4 | 1.93 | 1.04 | 1.99 | 0.96 |
| *Missing* | 1.48 | 1.32 | -1.55 | 1.6 | 1.48 | 1.33 | 1.60 | 1.25 |
| Dog ownership | | |  |  |  |  |  |  |
| *No* | 1.77 | 1.09 | -1.91 | 1.4 | 1.80 | 1.10 | 1.87 | 1.02 |
| *Yes* | 1.92 | 1.12 | -1.73 | 1.6 | 1.94 | 1.10 | 2.01 | 1.02 |
| Country |  |  |  |  |  |  |  |  |
| *Australia* | 2.07 | 1.01 | -1.76 | 1.5 | 2.03 | 0.99 | 2.09 | 0.93 |
| *Bulgaria* | 1.78 | 1.28 | -1.91 | 1.4 | 1.99 | 1.20 | 1.99 | 1.16 |
| *California* | 2.02 | 1.05 | -1.83 | 1.5 | 2.08 | 1.06 | 2.11 | 1.00 |
| *Canada* | 1.89 | 1.11 | -1.51 | 1.7 | 1.93 | 1.04 | 1.94 | 1.01 |
| *Czech Republic* | 1.96 | 1.06 | -2.07 | 1.2 | 1.54 | 1.26 | 1.83 | 1.05 |
| *Estonia* | 1.56 | 1.18 | -1.78 | 1.4 | 1.75 | 1.09 | 1.89 | 0.96 |
| *Finland* | 1.67 | 1.11 | -2.35 | 1.1 | 1.67 | 1.12 | 1.93 | 0.98 |
| *France* | 1.78 | 1.11 | -2.18 | 1.2 | 1.80 | 1.14 | 1.90 | 1.02 |
| *Germany* | 1.55 | 1.20 | -1.97 | 1.3 | 1.73 | 1.15 | 1.80 | 1.10 |
| *Greece* | 2.09 | 0.96 | -1.68 | 1.4 | 2.16 | 0.94 | 2.06 | 0.98 |
| *Hong Kong* | 1.29 | 1.03 | -1.40 | 1.3 | 1.32 | 1.04 | 1.32 | 1.00 |
| *Ireland* | 1.90 | 1.10 | -1.60 | 1.6 | 1.86 | 1.09 | 1.92 | 1.05 |
| *Italy* | 1.73 | 1.06 | -1.64 | 1.6 | 1.81 | 1.04 | 1.82 | 1.00 |
| *Netherlands* | 1.49 | 1.15 | -2.04 | 1.4 | 1.59 | 1.11 | 1.70 | 0.99 |
| *Portugal* | 2.10 | 0.88 | -1.68 | 1.5 | 2.16 | 0.90 | 2.16 | 0.87 |
| *Spain* | 1.94 | 1.07 | -1.45 | 1.7 | 2.00 | 1.07 | 1.95 | 1.05 |
| *Sweden* | 1.78 | 1.13 | -2.21 | 1.3 | 1.70 | 1.19 | 1.90 | 1.04 |
| *United Kingdom (ref)* | 1.94 | 0.98 | -2.22 | 1.1 | 1.95 | 0.97 | 2.00 | 0.88 |
| Survey wave | |  |  |  |  |  |  |  |
| *Jun-17* | 1.86 | 1.08 | -1.87 | 1.4 | 1.88 | 1.08 | 1.94 | 1.00 |
| *Sep-17* | 1.86 | 1.10 | -1.89 | 1.4 | 1.90 | 1.10 | 1.96 | 1.02 |
| *Dec-17* | 1.75 | 1.09 | -1.81 | 1.5 | 1.78 | 1.10 | 1.85 | 1.03 |
| *Mar-18* | 1.78 | 1.15 | -1.83 | 1.5 | 1.80 | 1.13 | 1.89 | 1.03 |
| **Travel characteristics** | |  |  |  |  |  |  |  |
| Visit origin | |  |  |  |  |  |  |  |
| *Your home (ref)* | 1.85 | 1.07 | -1.88 | 1.4 | 1.87 | 1.08 | 1.94 | 0.99 |
| *Your place of work* | 1.19 | 1.40 | -1.36 | 1.6 | 1.31 | 1.28 | 1.40 | 1.18 |
| *Holiday accommodation* | 1.95 | 1.09 | -1.92 | 1.4 | 2.02 | 1.07 | 2.07 | 1.01 |
| *Elsewhere* | 1.57 | 1.17 | -1.79 | 1.4 | 1.60 | 1.18 | 1.68 | 1.14 |
| Travel mode | |  |  |  |  |  |  |  |
| *On foot/bike* | 1.72 | 1.08 | -1.99 | 1.3 | 1.74 | 1.11 | 1.83 | 1.01 |
| *Other (e.g. horseback)* | 1.82 | 1.27 | -1.84 | 1.5 | 1.80 | 1.27 | 1.88 | 1.15 |
| *Public transport* | 1.48 | 1.29 | -1.47 | 1.6 | 1.54 | 1.22 | 1.64 | 1.15 |
| *Private vehicle* | 1.94 | 1.06 | -1.82 | 1.5 | 1.98 | 1.05 | 2.02 | 0.98 |
| Travel duration | |  |  |  |  |  |  |  |
| *<15 mins (ref)* | 1.79 | 1.08 | -2.10 | 1.2 | 1.83 | 1.08 | 1.92 | 1.00 |
| *15 - <30 mins* | 1.85 | 1.02 | -2.05 | 1.2 | 1.86 | 1.04 | 1.94 | 0.94 |
| *30 - <60 mins* | 1.86 | 1.01 | -1.89 | 1.4 | 1.87 | 1.04 | 1.91 | 0.98 |
| *60 - <120 mins* | 1.82 | 1.16 | -1.56 | 1.6 | 1.83 | 1.15 | 1.90 | 1.06 |
| *>=120 mins* | 1.73 | 1.31 | -1.42 | 1.7 | 1.81 | 1.25 | 1.86 | 1.17 |

Table S4 Linear modelling results for visit happiness and each modelling step.

| Happy | | | | | | |
| --- | --- | --- | --- | --- | --- | --- |
| names | Bluespace type | Visitor characteristics | Bluespace qualities | Exposure | Travel | Experience |
|  | Coefficient [95% confidence interval] | | | | | |
| Intercept | **1.80 *** [1.77, 1.83]** | **0.79 *** [0.66, 0.91]** | **0.78 *** [0.67, 0.89]** | **0.55 *** [0.44, 0.65]** | **0.60 *** [0.49, 0.71]** | **0.55 *** [0.44, 0.67]** |
| **RQ1 Natural and environmental features** | |  |  |  |  |  |
| 1a) Bluespace type |  |  |  |  |  |  |
| *Seaside promenade* | **0.08 ** [0.03, 0.12]** | **0.08 ** [0.03, 0.12]** | **0.09 *** [0.04, 0.13]** | **0.08 *** [0.04, 0.13]** | **0.07 *** [0.03, 0.11]** | **0.06 ** [0.02, 0.10]** |
| *Lake/reservoir* | **0.09 *** [0.04, 0.14]** | **0.09 *** [0.04, 0.14]** | 0.02 [-0.02, 0.07] | 0.02 [-0.02, 0.07] | 0.01 [-0.04, 0.05] | 0.00 [-0.04, 0.04] |
| *Sandy beach or dunes* | **0.41 *** [0.35, 0.47]** | **0.27 *** [0.22, 0.33]** | **0.19 *** [0.14, 0.24]** | **0.14 *** [0.09, 0.19]** | **0.13 *** [0.08, 0.18]** | **0.11 *** [0.06, 0.16]** |
| *Small water bodies* | **-0.11 *** [-0.17, -0.05]** | **-0.11 *** [-0.17, -0.06]** | **-0.08 ** [-0.13, -0.03]** | -0.01 [-0.06, 0.04] | -0.02 [-0.07, 0.03] | -0.03 [-0.08, 0.03] |
| *Rural river/canal* | 0.05 [-0.01, 0.11] | 0.02 [-0.04, 0.07] | 0.02 [-0.03, 0.07] | 0.05 [-0.00, 0.10] | 0.04 [-0.01, 0.09] | 0.04 [-0.02, 0.09] |
| *Harbour or marina* | 0.03 [-0.04, 0.11] | -0.02 [-0.09, 0.05] | -0.01 [-0.07, 0.06] | -0.02 [-0.08, 0.04] | -0.02 [-0.08, 0.04] | -0.03 [-0.09, 0.03] |
| *Water feature/fountain* | **-0.37 *** [-0.45, -0.29]** | **-0.27 *** [-0.35, -0.20]** | **-0.14 *** [-0.20, -0.07]** | -0.01 [-0.08, 0.05] | -0.01 [-0.08, 0.05] | -0.03 [-0.09, 0.04] |
| *Outdoor pool/spa* | -0.08 [-0.17, 0.01] | -0.07 [-0.15, 0.01] | -0.06 [-0.13, 0.02] | **-0.11 ** [-0.18, -0.03]** | **-0.11 ** [-0.19, -0.04]** | **-0.12 ** [-0.19, -0.04]** |
| *Pier* | **-0.17 ** [-0.27, -0.06]** | -0.05 [-0.15, 0.04] | -0.01 [-0.09, 0.08] | 0.00 [-0.08, 0.09] | 0.01 [-0.08, 0.09] | -0.01 [-0.09, 0.08] |
| *Open sea* | **0.22 *** [0.12, 0.33]** | **0.19 *** [0.10, 0.29]** | **0.13 ** [0.04, 0.21]** | 0.07 [-0.02, 0.15] | 0.08 [-0.00, 0.17] | 0.09 [-0.00, 0.17] |
| *Waterfall or rapids* | **-0.24 *** [-0.37, -0.11]** | **-0.17 ** [-0.28, -0.05]** | **-0.22 *** [-0.32, -0.11]** | **-0.20 *** [-0.30, -0.10]** | **-0.18 ** [-0.28, -0.07]** | **-0.15 ** [-0.26, -0.05]** |
| *Ice rink* | **-0.21 ** [-0.34, -0.08]** | **-0.17 ** [-0.29, -0.05]** | -0.02 [-0.13, 0.09] | -0.05 [-0.16, 0.06] | -0.06 [-0.17, 0.05] | 0.03 [-0.08, 0.15] |
| *Rocky or stony shore* | **0.35 *** [0.22, 0.48]** | **0.31 *** [0.19, 0.43]** | **0.19 *** [0.08, 0.30]** | **0.15 ** [0.04, 0.26]** | **0.15 ** [0.04, 0.25]** | **0.12 * [0.02, 0.23]** |
| *Fen, marsh or bog* | **-0.29 *** [-0.44, -0.14]** | **-0.21 ** [-0.35, -0.08]** | **-0.21 *** [-0.33, -0.09]** | **-0.23 *** [-0.35, -0.11]** | **-0.18 ** [-0.30, -0.06]** | **-0.17 ** [-0.29, -0.05]** |
| *Sea cliffs* | **0.32 *** [0.15, 0.48]** | **0.25 ** [0.10, 0.40]** | **0.17 * [0.04, 0.31]** | **0.14 * [0.01, 0.27]** | **0.14 * [0.01, 0.27]** | 0.13 [-0.00, 0.26] |
| *Salt marsh, estuary or lagoon* | 0.17 [-0.07, 0.41] | 0.07 [-0.16, 0.29] | -0.01 [-0.22, 0.19] | -0.05 [-0.25, 0.15] | -0.05 [-0.25, 0.15] | -0.05 [-0.25, 0.14] |
| *Urban river/canal* | - | - | - | - | - | - |
| **Visitor characteristics** |  |  |  |  |  |  |
| Perceived financial strain |  |  |  |  |  |  |
| *Finding it very difficult* |  | **0.09 * [0.02, 0.17]** | **0.12 *** [0.05, 0.19]** | **0.12 *** [0.05, 0.19]** | **0.11 *** [0.05, 0.18]** | **0.12 *** [0.05, 0.18]** |
| *Finding it difficult* |  | 0.04 [-0.01, 0.09] | **0.06 ** [0.02, 0.10]** | **0.05 * [0.01, 0.09]** | **0.05 * [0.01, 0.09]** | **0.05 * [0.01, 0.09]** |
| *Coping (ref)* |  |  |  |  |  |  |
| *Living comfortably* |  | **-0.04 * [-0.08, -0.01]** | **-0.07 *** [-0.10, -0.03]** | **-0.07 *** [-0.10, -0.03]** | **-0.07 *** [-0.10, -0.03]** | **-0.07 *** [-0.10, -0.03]** |
| *Missing* |  | **-0.46 *** [-0.61, -0.30]** | **-0.28 *** [-0.42, -0.14]** | **-0.28 *** [-0.41, -0.14]** | **-0.25 *** [-0.39, -0.12]** | **-0.24 *** [-0.37, -0.10]** |
| Age |  |  |  |  |  |  |
| *60+* |  | **0.11 *** [0.05, 0.18]** | 0.03 [-0.03, 0.09] | 0.04 [-0.02, 0.10] | 0.03 [-0.03, 0.08] | 0.02 [-0.04, 0.08] |
| *50-59* |  | **0.15 *** [0.09, 0.21]** | 0.05 [-0.00, 0.10] | 0.05 [-0.00, 0.10] | 0.04 [-0.01, 0.09] | 0.03 [-0.02, 0.09] |
| *40-49* |  | **0.15 *** [0.09, 0.21]** | **0.06 * [0.00, 0.11]** | **0.06 * [0.01, 0.11]** | 0.05 [-0.00, 0.10] | 0.05 [-0.00, 0.10] |
| *30-39* |  | 0.04 [-0.01, 0.10] | 0.00 [-0.05, 0.06] | 0.01 [-0.04, 0.06] | 0.00 [-0.05, 0.05] | -0.00 [-0.05, 0.05] |
| *18 – 29 (ref)* |  |  |  |  |  |  |
| Gender |  |  |  |  |  |  |
| *Male* |  | **-0.20 *** [-0.23, -0.17]** | **-0.18 *** [-0.21, -0.15]** | **-0.18 *** [-0.21, -0.15]** | **-0.18 *** [-0.21, -0.15]** | **-0.17 *** [-0.20, -0.15]** |
| *Female (ref)* |  |  |  |  |  |  |
| Limiting illness/disability | |  |  |  |  |  |
| *Yes a lot* |  | **-0.11 *** [-0.17, -0.04]** | **-0.21 *** [-0.27, -0.16]** | **-0.22 *** [-0.28, -0.17]** | **-0.19 *** [-0.25, -0.14]** | **-0.17 *** [-0.23, -0.12]** |
| *Yes to some extent* |  | **-0.05 ** [-0.09, -0.01]** | **-0.06 *** [-0.10, -0.03]** | **-0.06 *** [-0.10, -0.03]** | **-0.05 ** [-0.09, -0.02]** | **-0.05 ** [-0.08, -0.01]** |
| *No (ref)* |  |  |  |  |  |  |
| Garden access |  |  |  |  |  |  |
| *Private garden* |  | **0.08 *** [0.04, 0.13]** | 0.02 [-0.02, 0.06] | 0.02 [-0.02, 0.07] | 0.02 [-0.02, 0.06] | 0.02 [-0.02, 0.07] |
| *Private outdoor space* |  | 0.05 [-0.00, 0.10] | 0.02 [-0.03, 0.06] | 0.02 [-0.03, 0.07] | 0.02 [-0.03, 0.07] | 0.02 [-0.02, 0.07] |
| *Communal Garden* |  | -0.02 [-0.08, 0.04] | **-0.08 ** [-0.13, -0.03]** | **-0.09 ** [-0.14, -0.03]** | **-0.07 ** [-0.13, -0.02]** | **-0.07 * [-0.12, -0.02]** |
| *No access (ref)* |  |  |  |  |  |  |
| Employment status |  |  |  |  |  |  |
| *Employed (ref)* |  |  |  |  |  |  |
| *In education* |  | -0.04 [-0.12, 0.03] | -0.01 [-0.08, 0.05] | 0.01 [-0.06, 0.07] | 0.00 [-0.06, 0.07] | -0.00 [-0.07, 0.06] |
| *Housework* |  | 0.05 [-0.02, 0.13] | 0.04 [-0.03, 0.11] | 0.04 [-0.03, 0.11] | 0.03 [-0.04, 0.10] | 0.02 [-0.05, 0.09] |
| *Retired* |  | -0.05 [-0.11, 0.01] | -0.04 [-0.09, 0.01] | -0.04 [-0.09, 0.01] | -0.05 [-0.10, 0.00] | -0.05 [-0.10, 0.00] |
| *Disabled* |  | **0.18 *** [0.08, 0.28]** | **0.16 *** [0.07, 0.26]** | **0.16 *** [0.07, 0.25]** | **0.14 ** [0.05, 0.23]** | **0.13 ** [0.04, 0.22]** |
| *Unemployed* |  | -0.07 [-0.14, 0.00] | **-0.07 * [-0.13, -0.00]** | **-0.07 * [-0.13, -0.00]** | **-0.07 * [-0.13, -0.00]** | -0.06 [-0.12, 0.00] |
| *Other/unsure* |  | -0.03 [-0.12, 0.05] | -0.01 [-0.09, 0.06] | -0.02 [-0.09, 0.05] | -0.02 [-0.10, 0.05] | -0.01 [-0.09, 0.06] |
| Educational attainment |  |  |  |  |  |  |
| *Primary* |  | **-0.20 *** [-0.27, -0.14]** | **-0.15 *** [-0.21, -0.09]** | **-0.16 *** [-0.22, -0.10]** | **-0.14 *** [-0.20, -0.09]** | **-0.14 *** [-0.20, -0.08]** |
| *Secondary* |  | -0.00 [-0.04, 0.03] | 0.01 [-0.02, 0.04] | 0.01 [-0.02, 0.04] | 0.01 [-0.02, 0.04] | 0.00 [-0.03, 0.03] |
| *University (ref)* |  |  |  |  |  |  |
| Perceived minority ethnic group member | |  |  |  |  |  |
| *Yes* |  | **-0.07 * [-0.13, -0.00]** | -0.05 [-0.11, 0.01] | **-0.07 * [-0.12, -0.01]** | -0.04 [-0.10, 0.01] | -0.03 [-0.09, 0.02] |
| *Do not know* |  | **-0.26 *** [-0.35, -0.17]** | **-0.17 *** [-0.26, -0.09]** | **-0.17 *** [-0.25, -0.08]** | **-0.15 *** [-0.23, -0.07]** | **-0.14 *** [-0.22, -0.06]** |
| *No (ref)* |  |  |  |  |  |  |
| Relationship status |  |  |  |  |  |  |
| *Married/cohabiting* |  | **0.05 ** [0.01, 0.09]** | **0.05 ** [0.01, 0.08]** | **0.04 * [0.00, 0.07]** | **0.03 * [0.00, 0.06]** | 0.03 [-0.00, 0.06] |
| *Missing* |  | -0.05 [-0.13, 0.03] | -0.02 [-0.09, 0.05] | -0.03 [-0.10, 0.05] | -0.02 [-0.09, 0.05] | -0.01 [-0.08, 0.06] |
| *Single (ref)* |  |  |  |  |  |  |
| Dog owner |  |  |  |  |  |  |
| *Yes* |  | **0.06 ** [0.02, 0.09]** | 0.02 [-0.01, 0.05] | 0.01 [-0.02, 0.04] | 0.01 [-0.02, 0.04] | -0.00 [-0.04, 0.03] |
| *No (ref)* |  |  |  |  |  |  |
| Survey wave |  |  |  |  |  |  |
| *Mar-18* |  | **-0.07 ** [-0.11, -0.02]** | -0.04 [-0.08, 0.00] | -0.01 [-0.05, 0.03] | -0.01 [-0.05, 0.03] | -0.00 [-0.04, 0.04] |
| *Dec-17* |  | **-0.08 *** [-0.13, -0.04]** | **-0.06 ** [-0.10, -0.02]** | -0.03 [-0.07, 0.01] | -0.03 [-0.07, 0.01] | -0.03 [-0.07, 0.01] |
| *Sep-17* |  | -0.03 [-0.07, 0.01] | -0.02 [-0.06, 0.02] | -0.03 [-0.07, 0.01] | -0.03 [-0.07, 0.01] | -0.03 [-0.06, 0.01] |
| *June-17 (ref)* |  |  |  |  |  |  |
| WHO-5 Wellbeing Index |  | **0.02 *** [0.02, 0.02]** | **0.01 *** [0.01, 0.01]** | **0.01 *** [0.01, 0.01]** | **0.01 *** [0.01, 0.01]** | **0.01 *** [0.01, 0.01]** |
| **1b) Perceived bluespace qualities** | |  |  |  |  |  |
| Safety |  |  | **0.30 *** [0.29, 0.31]** | **0.29 *** [0.28, 0.30]** | **0.29 *** [0.27, 0.30]** | **0.28 *** [0.27, 0.30]** |
| Presence of wildlife |  |  | **0.07 *** [0.06, 0.08]** | **0.07 *** [0.06, 0.08]** | **0.07 *** [0.06, 0.08]** | **0.07 *** [0.06, 0.08]** |
| Absence of litter/vandalism |  |  | **0.03 *** [0.02, 0.05]** | **0.04 *** [0.03, 0.05]** | **0.04 *** [0.03, 0.05]** | **0.04 *** [0.03, 0.05]** |
| Presence of good facilities |  |  | **0.03 *** [0.02, 0.04]** | **0.02 *** [0.01, 0.03]** | **0.02 *** [0.01, 0.03]** | **0.03 *** [0.01, 0.04]** |
| Water quality |  |  |  |  |  |  |
| *Excellent* |  |  | **0.21 *** [0.16, 0.26]** | **0.19 *** [0.14, 0.23]** | **0.20 *** [0.15, 0.24]** | **0.20 *** [0.15, 0.25]** |
| *Good* |  |  | **0.05 ** [0.01, 0.09]** | **0.04 * [0.01, 0.08]** | **0.05 * [0.01, 0.08]** | **0.05 * [0.01, 0.09]** |
| *Sufficient (ref)* |  |  |  |  |  |  |
| *Poor* |  |  | -0.01 [-0.07, 0.05] | 0.01 [-0.06, 0.07] | -0.01 [-0.07, 0.06] | -0.01 [-0.07, 0.06] |
| **RQ2. Exposure** |  |  |  |  |  |  |
| *>=3 hr* |  |  |  | **0.45 *** [0.39, 0.50]** | **0.47 *** [0.42, 0.53]** | **0.45 *** [0.39, 0.51]** |
| *2 - <3 hr* |  |  |  | **0.39 *** [0.34, 0.44]** | **0.40 *** [0.35, 0.45]** | **0.39 *** [0.33, 0.44]** |
| *1.5 - <2 hr* |  |  |  | **0.38 *** [0.31, 0.44]** | **0.38 *** [0.31, 0.44]** | **0.37 *** [0.31, 0.43]** |
| *1 - <1.5 hr* |  |  |  | **0.31 *** [0.27, 0.36]** | **0.32 *** [0.27, 0.36]** | **0.31 *** [0.26, 0.36]** |
| *30 mins - <1 hr* |  |  |  | **0.22 *** [0.17, 0.26]** | **0.22 *** [0.17, 0.26]** | **0.21 *** [0.16, 0.26]** |
| *<30 mins (ref)* |  |  |  |  |  |  |
| **Travel characteristics** |  |  |  |  |  |  |
| Travel duration |  |  |  |  |  |  |
| *>=120 mins* |  |  |  |  | **-0.15 *** [-0.20, -0.09]** | **-0.12 *** [-0.17, -0.07]** |
| *60 - <120 mins* |  |  |  |  | **-0.05 * [-0.09, -0.00]** | -0.03 [-0.08, 0.01] |
| *30 - <60 mins* |  |  |  |  | 0.04 [-0.01, 0.08] | 0.04 [-0.01, 0.09] |
| *15 - <30 mins* |  |  |  |  | 0.03 [-0.01, 0.07] | 0.03 [-0.01, 0.07] |
| *<15 mins (ref)* |  |  |  |  |  |  |
| Travel mode |  |  |  |  |  |  |
| *Private vehicle* |  |  |  |  | -0.01 [-0.04, 0.03] | -0.01 [-0.05, 0.02] |
| *Public transport* |  |  |  |  | **-0.09 *** [-0.15, -0.04]** | **-0.09 ** [-0.15, -0.03]** |
| *Other* |  |  |  |  | 0.03 [-0.09, 0.14] | 0.04 [-0.07, 0.15] |
| *On foot/bike (ref)* |  |  |  |  |  |  |
| Travel origin |  |  |  |  |  |  |
| *Place of work* |  |  |  |  | **-0.26 *** [-0.33, -0.18]** | **-0.24 *** [-0.31, -0.17]** |
| *Holiday accommodation* |  |  |  |  | -0.01 [-0.07, 0.04] | -0.01 [-0.07, 0.04] |
| *Elsewhere* |  |  |  |  | **-0.06 * [-0.12, -0.00]** | **-0.07 * [-0.13, -0.01]** |
| *Home (ref)* |  |  |  |  |  |  |
| **RQ3. Experience** |  |  |  |  |  |  |
| Activity |  |  |  |  |  |  |
| *Walking without a dog (ref)* |  |  |  |  |  |  |
| *Walking with a dog* |  |  |  |  |  | **0.10 *** [0.05, 0.16]** |
| *Socialising* |  |  |  |  |  | **0.12 *** [0.07, 0.18]** |
| *Appreciating nature* |  |  |  |  |  | 0.06 [-0.00, 0.12] |
| *Swimming* |  |  |  |  |  | 0.04 [-0.02, 0.11] |
| *Other* |  |  |  |  |  | **-0.13 *** [-0.19, -0.07]** |
| *Playing with children* |  |  |  |  |  | **0.24 *** [0.17, 0.31]** |
| *Running/nordic walking* |  |  |  |  |  | **-0.08 * [-0.15, -0.01]** |
| *Sunbathing/paddling* |  |  |  |  |  | **0.12 ** [0.04, 0.19]** |
| *Eating or drinking* |  |  |  |  |  | 0.04 [-0.03, 0.11] |
| *Quiet activities* |  |  |  |  |  | **0.09 * [0.01, 0.17]** |
| *Winter activities* |  |  |  |  |  | **-0.12 ** [-0.21, -0.03]** |
| *Cycling* |  |  |  |  |  | -0.02 [-0.10, 0.06] |
| *Watersports/boating* |  |  |  |  |  | -0.01 [-0.09, 0.08] |
| *Visiting an attraction* |  |  |  |  |  | 0.04 [-0.05, 0.14] |
| *Fishing* |  |  |  |  |  | 0.11 [-0.00, 0.23] |
| *Sport* |  |  |  |  |  | **-0.22 ** [-0.35, -0.08]** |
| *Conservation* |  |  |  |  |  | **-0.41 ** [-0.66, -0.16]** |
| Visit companions |  |  |  |  |  |  |
| *Adults and children* |  |  |  |  |  | **-0.05 * [-0.10, -0.01]** |
| *Other children only* |  |  |  |  |  | -0.01 [-0.08, 0.05] |
| *Other adults only* |  |  |  |  |  | 0.04 [-0.00, 0.07] |
| *Alone (ref)* |  |  |  |  |  |  |
|  |  |  |  |  |  |  |
| N | 14985 | 14957 | 14936 | 14930 | 14919 | 14892 |
| *R^2^* | 0.04 |  |  |  |  |  |
| *Pseudo-R^2^* |  | 0.19 | 0.35 | 0.36 | 0.37 | 0.38 |
| AIC | 45073.53 | 42609.82 | 39362.38 | 39025.14 | 38929.94 | 38847.61 |
| *** *p* < 0.001; ** *p* < 0.01; * *p* < 0.05. | | | | | | |

Table S5 Linear modelling results for visit anxiety and each modelling step.

| Anxious | | | | | | |
| --- | --- | --- | --- | --- | --- | --- |
| names | Bluespace type | Visitor characteristics | Bluespace qualities | Exposure | Travel | Experience |
|  | Coefficient [95% confidence interval] | | | | | |
| Intercept | **-1.77 *** [-1.81, -1.73]** | **-1.62 *** [-1.79, -1.45]** | **-1.50 *** [-1.67, -1.33]** | **-1.50 *** [-1.67, -1.32]** | **-1.63 *** [-1.81, -1.46]** | **-1.50 *** [-1.68, -1.31]** |
| **RQ1 Natural and environmental features** | |  |  |  |  |  |
| 1a) Bluespace type |  |  |  |  |  |  |
| *Seaside promenade* | **-0.13 *** [-0.19, -0.06]** | **-0.11 *** [-0.18, -0.05]** | **-0.11 *** [-0.17, -0.05]** | **-0.11 *** [-0.17, -0.05]** | **-0.09 ** [-0.15, -0.03]** | -0.05 [-0.11, 0.01] |
| *Lake/reservoir* | **-0.18 *** [-0.25, -0.11]** | -0.05 [-0.12, 0.02] | -0.06 [-0.12, 0.01] | -0.06 [-0.12, 0.01] | -0.04 [-0.11, 0.02] | -0.03 [-0.10, 0.03] |
| *Sandy beach or dunes* | **-0.25 *** [-0.33, -0.17]** | **-0.23 *** [-0.30, -0.16]** | **-0.20 *** [-0.27, -0.13]** | **-0.18 *** [-0.26, -0.11]** | **-0.17 *** [-0.24, -0.10]** | **-0.13 *** [-0.20, -0.06]** |
| *Small water bodies* | **-0.20 *** [-0.28, -0.12]** | -0.06 [-0.14, 0.01] | **-0.08 * [-0.16, -0.01]** | **-0.09 * [-0.17, -0.02]** | -0.07 [-0.14, 0.01] | -0.07 [-0.14, 0.00] |
| *Rural river/canal* | **-0.16 *** [-0.24, -0.08]** | **-0.09 * [-0.17, -0.02]** | **-0.10 ** [-0.18, -0.03]** | **-0.11 ** [-0.18, -0.04]** | **-0.09 * [-0.17, -0.02]** | **-0.08 * [-0.16, -0.01]** |
| *Harbour or marina* | **-0.12 * [-0.22, -0.02]** | -0.03 [-0.13, 0.06] | -0.04 [-0.13, 0.05] | -0.04 [-0.13, 0.05] | -0.03 [-0.13, 0.06] | -0.01 [-0.10, 0.09] |
| *Water feature/fountain* | 0.02 [-0.08, 0.12] | -0.07 [-0.17, 0.03] | -0.07 [-0.17, 0.02] | -0.09 [-0.19, 0.01] | -0.08 [-0.18, 0.01] | -0.07 [-0.17, 0.03] |
| *Outdoor pool/spa* | **0.27 *** [0.15, 0.38]** | **0.13 * [0.02, 0.24]** | **0.18 ** [0.07, 0.28]** | **0.19 *** [0.08, 0.30]** | **0.21 *** [0.10, 0.32]** | **0.19 *** [0.08, 0.31]** |
| *Pier* | **0.15 * [0.02, 0.29]** | 0.08 [-0.04, 0.21] | 0.08 [-0.04, 0.20] | 0.08 [-0.04, 0.20] | 0.07 [-0.05, 0.19] | 0.10 [-0.02, 0.22] |
| *Open sea* | 0.03 [-0.11, 0.16] | 0.03 [-0.10, 0.15] | 0.03 [-0.09, 0.16] | 0.05 [-0.07, 0.18] | 0.02 [-0.10, 0.15] | 0.02 [-0.11, 0.15] |
| *Waterfall or rapids* | **0.70 *** [0.53, 0.87]** | **0.33 *** [0.17, 0.49]** | **0.32 *** [0.17, 0.47]** | **0.32 *** [0.16, 0.47]** | **0.25 ** [0.09, 0.40]** | **0.21 ** [0.06, 0.36]** |
| *Ice rink* | **0.22 * [0.05, 0.39]** | **0.20 * [0.04, 0.36]** | **0.20 * [0.04, 0.36]** | **0.20 * [0.04, 0.36]** | **0.22 ** [0.07, 0.38]** | -0.01 [-0.18, 0.15] |
| *Rocky or stony shore* | **-0.38 *** [-0.55, -0.21]** | **-0.27 ** [-0.43, -0.10]** | **-0.23 ** [-0.39, -0.07]** | **-0.23 ** [-0.38, -0.07]** | **-0.22 ** [-0.37, -0.06]** | **-0.18 * [-0.33, -0.02]** |
| *Fen, marsh or bog* | **0.56 *** [0.37, 0.76]** | **0.33 *** [0.15, 0.51]** | **0.30 *** [0.13, 0.48]** | **0.30 *** [0.12, 0.48]** | **0.24 ** [0.06, 0.42]** | **0.22 * [0.04, 0.40]** |
| *Sea cliffs* | **-0.27 * [-0.48, -0.05]** | -0.18 [-0.38, 0.02] | -0.16 [-0.36, 0.03] | -0.16 [-0.35, 0.04] | -0.17 [-0.37, 0.02] | -0.13 [-0.32, 0.06] |
| *Salt marsh, estuary or lagoon* | -0.29 [-0.61, 0.03] | -0.09 [-0.38, 0.21] | -0.10 [-0.39, 0.20] | -0.10 [-0.39, 0.19] | -0.08 [-0.37, 0.21] | -0.06 [-0.35, 0.23] |
| *Urban river/canal* |  |  |  |  |  |  |
| **Visitor characteristics** |  |  |  |  |  |  |
| Perceived financial strain |  |  |  |  |  |  |
| *Finding it very difficult* |  | -0.02 [-0.12, 0.08] | -0.03 [-0.13, 0.07] | -0.02 [-0.12, 0.07] | -0.02 [-0.11, 0.08] | -0.02 [-0.12, 0.08] |
| *Finding it difficult* |  | 0.04 [-0.02, 0.10] | 0.04 [-0.02, 0.10] | 0.04 [-0.02, 0.10] | 0.04 [-0.02, 0.10] | 0.03 [-0.03, 0.09] |
| *Coping (ref)* |  |  |  |  |  |  |
| *Living comfortably* |  | 0.03 [-0.02, 0.08] | 0.03 [-0.02, 0.09] | 0.03 [-0.02, 0.09] | 0.04 [-0.02, 0.09] | 0.03 [-0.02, 0.08] |
| *Missing* |  | **0.40 *** [0.20, 0.60]** | **0.30 ** [0.10, 0.50]** | **0.30 ** [0.10, 0.50]** | **0.25 * [0.05, 0.45]** | **0.21 * [0.02, 0.41]** |
| Age |  |  |  |  |  |  |
| *60+* |  | **-0.59 *** [-0.68, -0.50]** | **-0.55 *** [-0.64, -0.46]** | **-0.56 *** [-0.64, -0.47]** | **-0.53 *** [-0.62, -0.44]** | **-0.49 *** [-0.58, -0.40]** |
| *50-59* |  | **-0.61 *** [-0.69, -0.53]** | **-0.56 *** [-0.64, -0.48]** | **-0.56 *** [-0.64, -0.48]** | **-0.54 *** [-0.62, -0.46]** | **-0.50 *** [-0.58, -0.42]** |
| *40-49* |  | **-0.42 *** [-0.50, -0.35]** | **-0.39 *** [-0.46, -0.31]** | **-0.39 *** [-0.47, -0.31]** | **-0.37 *** [-0.45, -0.30]** | **-0.38 *** [-0.46, -0.31]** |
| *30-39* |  | **-0.16 *** [-0.24, -0.09]** | **-0.15 *** [-0.22, -0.07]** | **-0.15 *** [-0.22, -0.08]** | **-0.14 *** [-0.22, -0.07]** | **-0.16 *** [-0.23, -0.09]** |
| *18 – 29 (ref)* |  |  |  |  |  |  |
| Gender |  |  |  |  |  |  |
| *Male* |  | **0.13 *** [0.09, 0.18]** | **0.12 *** [0.08, 0.16]** | **0.12 *** [0.08, 0.16]** | **0.13 *** [0.08, 0.17]** | **0.11 *** [0.07, 0.16]** |
| *Female (ref)* |  |  |  |  |  |  |
| Limiting illness/disability |  |  |  |  |  |  |
| *Yes a lot* |  | **1.13 *** [1.05, 1.22]** | **1.14 *** [1.06, 1.22]** | **1.14 *** [1.06, 1.22]** | **1.07 *** [0.99, 1.15]** | **1.03 *** [0.95, 1.11]** |
| *Yes to some extent* |  | **0.33 *** [0.27, 0.38]** | **0.31 *** [0.25, 0.36]** | **0.30 *** [0.25, 0.36]** | **0.28 *** [0.23, 0.33]** | **0.27 *** [0.22, 0.32]** |
| *No (ref)* |  |  |  |  |  |  |
| Garden access |  |  |  |  |  |  |
| *Private garden* |  | -0.03 [-0.10, 0.03] | -0.02 [-0.08, 0.05] | -0.02 [-0.08, 0.05] | -0.01 [-0.07, 0.05] | -0.02 [-0.08, 0.04] |
| *Private outdoor space* |  | **-0.08 * [-0.15, -0.01]** | -0.07 [-0.14, 0.00] | **-0.07 * [-0.14, -0.00]** | -0.06 [-0.13, 0.01] | **-0.07 * [-0.14, -0.00]** |
| *Communal Garden* |  | **0.23 *** [0.15, 0.31]** | **0.24 *** [0.16, 0.31]** | **0.24 *** [0.16, 0.31]** | **0.22 *** [0.14, 0.29]** | **0.19 *** [0.12, 0.27]** |
| *No access (ref)* |  |  |  |  |  |  |
| Employment status |  |  |  |  |  |  |
| *Employed (ref)* |  | **-0.15 ** [-0.25, -0.06]** | **-0.15 ** [-0.24, -0.05]** | **-0.15 ** [-0.25, -0.06]** | **-0.14 ** [-0.24, -0.05]** | **-0.12 * [-0.21, -0.03]** |
| *In education* |  |  |  |  |  |  |
| *Housework* |  | 0.03 [-0.07, 0.13] | 0.04 [-0.06, 0.14] | 0.04 [-0.06, 0.14] | 0.05 [-0.05, 0.15] | 0.03 [-0.07, 0.13] |
| *Retired* |  | -0.08 [-0.16, 0.00] | **-0.09 * [-0.17, -0.01]** | **-0.09 * [-0.16, -0.01]** | **-0.09 * [-0.16, -0.01]** | **-0.08 * [-0.16, -0.00]** |
| *Disabled* |  | **-0.46 *** [-0.60, -0.33]** | **-0.46 *** [-0.59, -0.32]** | **-0.46 *** [-0.59, -0.32]** | **-0.42 *** [-0.55, -0.29]** | **-0.40 *** [-0.53, -0.26]** |
| *Unemployed* |  | **-0.10 * [-0.19, -0.00]** | **-0.10 * [-0.19, -0.01]** | **-0.10 * [-0.19, -0.01]** | **-0.11 * [-0.20, -0.02]** | **-0.10 * [-0.19, -0.01]** |
| *Other/unsure* |  | -0.08 [-0.19, 0.03] | -0.08 [-0.19, 0.03] | -0.08 [-0.19, 0.03] | -0.09 [-0.20, 0.02] | -0.09 [-0.20, 0.02] |
| Educational attainment |  |  |  |  |  |  |
| *Primary* |  | **0.17 *** [0.08, 0.25]** | **0.13 ** [0.05, 0.22]** | **0.13 ** [0.05, 0.22]** | **0.11 * [0.02, 0.19]** | 0.08 [-0.00, 0.17] |
| *Secondary* |  | 0.03 [-0.02, 0.07] | 0.02 [-0.02, 0.07] | 0.03 [-0.02, 0.07] | 0.02 [-0.02, 0.07] | 0.02 [-0.02, 0.07] |
| *University (ref)* |  |  |  |  |  |  |
| Perceived minority ethnic group member | |  |  |  |  |  |
| *Yes* |  | **0.42 *** [0.33, 0.51]** | **0.40 *** [0.31, 0.48]** | **0.40 *** [0.31, 0.49]** | **0.35 *** [0.27, 0.44]** | **0.32 *** [0.24, 0.41]** |
| *Do not know* |  | **0.49 *** [0.37, 0.61]** | **0.45 *** [0.33, 0.57]** | **0.45 *** [0.33, 0.57]** | **0.42 *** [0.30, 0.54]** | **0.39 *** [0.27, 0.51]** |
| *No (ref)* |  |  |  |  |  |  |
| Relationship status |  |  |  |  |  |  |
| *Married/cohabiting* |  | **0.06 * [0.02, 0.11]** | **0.06 * [0.01, 0.11]** | **0.07 ** [0.02, 0.11]** | **0.06 * [0.01, 0.11]** | 0.04 [-0.01, 0.08] |
| *Missing* |  | 0.10 [-0.00, 0.21] | 0.08 [-0.03, 0.18] | 0.08 [-0.03, 0.18] | 0.07 [-0.03, 0.18] | 0.05 [-0.05, 0.16] |
| *Single (ref)* |  |  |  |  |  |  |
| Dog owner |  |  |  |  |  |  |
| *Yes* |  | **0.06 * [0.01, 0.11]** | **0.06 * [0.01, 0.10]** | **0.06 * [0.01, 0.10]** | **0.05 * [0.01, 0.10]** | 0.01 [-0.04, 0.06] |
| *No (ref)* |  |  |  |  |  |  |
| Survey wave |  |  |  |  |  |  |
| *Mar-18* |  | 0.03 [-0.03, 0.09] | 0.03 [-0.03, 0.09] | 0.03 [-0.03, 0.08] | 0.03 [-0.03, 0.08] | 0.02 [-0.04, 0.08] |
| *Dec-17* |  | **0.06 * [0.00, 0.12]** | **0.06 * [0.01, 0.12]** | 0.06 [-0.00, 0.12] | 0.05 [-0.01, 0.11] | 0.04 [-0.02, 0.10] |
| *Sep-17* |  | 0.01 [-0.05, 0.07] | 0.02 [-0.04, 0.07] | 0.02 [-0.04, 0.08] | 0.02 [-0.04, 0.08] | 0.01 [-0.05, 0.07] |
| *June-17 (ref)* |  |  |  |  |  |  |
| WHO-5 Wellbeing Index |  | **-0.00 *** [-0.00, -0.00]** | -0.00 [-0.00, 0.00] | -0.00 [-0.00, 0.00] | -0.00 [-0.00, 0.00] | **-0.00 * [-0.00, -0.00]** |
| **1b) Perceived bluespace qualities** | |  |  |  |  |  |
| Safety |  |  | **-0.21 *** [-0.23, -0.19]** | **-0.21 *** [-0.23, -0.19]** | **-0.20 *** [-0.22, -0.18]** | **-0.20 *** [-0.22, -0.18]** |
| Presence of wildlife |  |  | **0.06 *** [0.04, 0.07]** | **0.06 *** [0.05, 0.07]** | **0.06 *** [0.04, 0.07]** | **0.06 *** [0.05, 0.07]** |
| Absence of litter/vandalism |  |  | **-0.02 * [-0.04, -0.00]** | **-0.02 * [-0.04, -0.00]** | **-0.02 * [-0.03, -0.00]** | -0.01 [-0.03, 0.00] |
| Presence of good facilities |  |  | **0.04 *** [0.02, 0.05]** | **0.04 *** [0.03, 0.06]** | **0.03 *** [0.02, 0.05]** | **0.03 *** [0.02, 0.05]** |
| Water quality |  |  |  |  |  |  |
| *Excellent* |  |  | **-0.10 ** [-0.17, -0.03]** | **-0.09 * [-0.16, -0.02]** | **-0.11 ** [-0.18, -0.04]** | **-0.11 ** [-0.18, -0.04]** |
| *Good* |  |  | -0.02 [-0.08, 0.03] | -0.02 [-0.08, 0.04] | -0.03 [-0.09, 0.02] | -0.04 [-0.09, 0.02] |
| *Sufficient (ref)* |  |  |  |  |  |  |
| *Poor* |  |  | -0.03 [-0.12, 0.06] | -0.03 [-0.12, 0.06] | -0.01 [-0.10, 0.08] | -0.02 [-0.11, 0.07] |
| **RQ2. Exposure** |  |  |  |  |  |  |
| Visit duration |  |  |  |  |  |  |
| *>=3 hr* |  |  |  | -0.07 [-0.15, 0.01] | **-0.19 *** [-0.27, -0.11]** | **-0.23 *** [-0.32, -0.15]** |
| *2 - <3 hr* |  |  |  | -0.02 [-0.10, 0.05] | **-0.10 * [-0.18, -0.02]** | **-0.14 *** [-0.22, -0.06]** |
| *1.5 - <2 hr* |  |  |  | -0.06 [-0.16, 0.03] | **-0.12 * [-0.21, -0.02]** | **-0.16 *** [-0.25, -0.06]** |
| *1 - <1.5 hr* |  |  |  | 0.01 [-0.06, 0.08] | -0.04 [-0.11, 0.03] | -0.07 [-0.14, 0.00] |
| *30 mins - <1 hr* |  |  |  | 0.07 [-0.00, 0.13] | 0.04 [-0.03, 0.11] | 0.02 [-0.05, 0.09] |
| *<30 mins (ref)* |  |  |  |  |  |  |
| **Travel characteristics** |  |  |  |  |  |  |
| Travel duration |  |  |  |  |  |  |
| *>=120 mins* |  |  |  |  | **0.41 *** [0.33, 0.48]** | **0.36 *** [0.29, 0.44]** |
| *60 - <120 mins* |  |  |  |  | **0.34 *** [0.27, 0.40]** | **0.32 *** [0.25, 0.39]** |
| *30 - <60 mins* |  |  |  |  | **0.11 *** [0.05, 0.18]** | **0.12 *** [0.05, 0.19]** |
| *15 - <30 mins* |  |  |  |  | -0.01 [-0.07, 0.05] | 0.00 [-0.06, 0.06] |
| *<15 mins (ref)* |  |  |  |  |  |  |
| Travel mode |  |  |  |  |  |  |
| *Private vehicle* |  |  |  |  | **0.07 ** [0.02, 0.12]** | **0.07 ** [0.02, 0.12]** |
| *Public transport* |  |  |  |  | **0.15 *** [0.07, 0.23]** | **0.14 *** [0.06, 0.22]** |
| *Other* |  |  |  |  | 0.01 [-0.16, 0.17] | -0.01 [-0.17, 0.16] |
| *On foot/bike (ref)* |  |  |  |  |  |  |
| Travel origin |  |  |  |  |  |  |
| *Place of work* |  |  |  |  | **0.12 * [0.02, 0.23]** | **0.11 * [0.00, 0.21]** |
| *Holiday accommodation* |  |  |  |  | -0.06 [-0.14, 0.02] | -0.05 [-0.13, 0.04] |
| *Elsewhere* |  |  |  |  | -0.01 [-0.09, 0.08] | 0.01 [-0.08, 0.10] |
| *Home (ref)* |  |  |  |  |  |  |
| **RQ3. Experience** |  |  |  |  |  |  |
| Activity |  |  |  |  |  |  |
| *Walking without a dog (ref)* |  |  |  |  |  |  |
| *Walking with a dog* |  |  |  |  |  | -0.05 [-0.12, 0.03] |
| *Socialising* |  |  |  |  |  | **-0.13 ** [-0.22, -0.05]** |
| *Appreciating nature* |  |  |  |  |  | **-0.20 *** [-0.29, -0.11]** |
| *Swimming* |  |  |  |  |  | -0.08 [-0.18, 0.01] |
| *Other* |  |  |  |  |  | 0.04 [-0.05, 0.13] |
| *Playing with children* |  |  |  |  |  | **-0.21 *** [-0.31, -0.11]** |
| *Running/nordic walking* |  |  |  |  |  | **0.30 *** [0.20, 0.40]** |
| *Sunbathing/paddling* |  |  |  |  |  | **-0.21 *** [-0.31, -0.10]** |
| *Eating or drinking* |  |  |  |  |  | **-0.18 ** [-0.28, -0.07]** |
| *Quiet activities* |  |  |  |  |  | **-0.27 *** [-0.39, -0.16]** |
| *Winter activities* |  |  |  |  |  | **0.28 *** [0.15, 0.41]** |
| *Cycling* |  |  |  |  |  | 0.03 [-0.09, 0.15] |
| *Watersports/boating* |  |  |  |  |  | 0.05 [-0.08, 0.17] |
| *Visiting an attraction* |  |  |  |  |  | -0.10 [-0.24, 0.04] |
| *Fishing* |  |  |  |  |  | -0.00 [-0.17, 0.16] |
| *Sport* |  |  |  |  |  | **0.30 ** [0.11, 0.50]** |
| *Conservation* |  |  |  |  |  | **0.63 *** [0.26, 1.00]** |
| Visit companions |  |  |  |  |  |  |
| *Adults and children* |  |  |  |  |  | **0.23 *** [0.16, 0.29]** |
| *Other children only* |  |  |  |  |  | **0.14 ** [0.05, 0.23]** |
| *Other adults only* |  |  |  |  |  | -0.02 [-0.07, 0.04] |
| *Alone (ref)* |  |  |  |  |  |  |
|  |  |  |  |  |  |  |
| N | 14984 | 14956 | 14935 | 14929 | 14918 | 14891 |
| *R^2^* | 0.02 |  |  |  |  |  |
| *Pseudo-R^2^* |  | 0.16 | 0.19 | 0.20 | 0.21 | 0.22 |
| AIC | 53279.80 | 51092.76 | 50529.78 | 50527.38 | 50306.38 | 50088.25 |
| *** *p* < 0.001; ** *p* < 0.01; * *p* < 0.05. | | | | | | |

**Table S6** Linear modelling results for visit worthwhileness and each modelling step.

| Worthwhile | | | | | | |
| --- | --- | --- | --- | --- | --- | --- |
| names | Bluespace type | Visitor characteristics | Bluespace qualities | Exposure | Travel | Experience |
|  | Coefficient [95% confidence interval] | | | | | |
| Intercept | **1.84 *** [1.81, 1.87]** | **0.84 *** [0.72, 0.96]** | **0.86 *** [0.74, 0.97]** | **0.59 *** [0.48, 0.70]** | **0.67 *** [0.55, 0.78]** | **0.64 *** [0.53, 0.76]** |
| **RQ1 Natural and environmental features** | |  |  |  |  |  |
| 1a) Bluespace type |  |  |  |  |  |  |
| *Seaside promenade* | **0.09 *** [0.05, 0.14]** | **0.08 ** [0.03, 0.12]** | **0.08 *** [0.04, 0.12]** | **0.08 *** [0.04, 0.12]** | **0.07 *** [0.03, 0.11]** | **0.06 ** [0.02, 0.10]** |
| *Lake/reservoir* | **0.07 * [0.02, 0.12]** | **0.07 ** [0.02, 0.12]** | 0.00 [-0.04, 0.05] | -0.00 [-0.05, 0.04] | -0.01 [-0.06, 0.03] | -0.02 [-0.06, 0.03] |
| *Sandy beach or dunes* | **0.42 *** [0.37, 0.48]** | **0.28 *** [0.22, 0.33]** | **0.19 *** [0.14, 0.24]** | **0.14 *** [0.09, 0.19]** | **0.13 *** [0.08, 0.18]** | **0.11 *** [0.06, 0.16]** |
| *Small water bodies* | **-0.18 *** [-0.24, -0.12]** | **-0.16 *** [-0.21, -0.10]** | **-0.12 *** [-0.17, -0.07]** | -0.04 [-0.09, 0.01] | **-0.05 * [-0.10, -0.00]** | **-0.06 * [-0.11, -0.01]** |
| *Rural river/canal* | 0.02 [-0.04, 0.08] | -0.01 [-0.07, 0.04] | -0.00 [-0.05, 0.05] | 0.03 [-0.02, 0.08] | 0.02 [-0.03, 0.07] | 0.02 [-0.03, 0.07] |
| *Harbour or marina* | 0.07 [-0.00, 0.15] | 0.01 [-0.06, 0.08] | 0.02 [-0.04, 0.09] | 0.01 [-0.05, 0.07] | 0.01 [-0.05, 0.07] | 0.00 [-0.06, 0.07] |
| *Water feature/fountain* | **-0.39 *** [-0.47, -0.31]** | **-0.32 *** [-0.39, -0.24]** | **-0.19 *** [-0.26, -0.12]** | -0.05 [-0.12, 0.01] | -0.06 [-0.12, 0.01] | -0.06 [-0.13, 0.00] |
| *Outdoor pool/spa* | -0.08 [-0.17, 0.01] | -0.04 [-0.12, 0.04] | -0.04 [-0.12, 0.03] | **-0.10 * [-0.17, -0.02]** | **-0.10 ** [-0.18, -0.03]** | **-0.12 ** [-0.19, -0.04]** |
| *Pier* | **-0.21 *** [-0.31, -0.11]** | -0.09 [-0.18, 0.01] | -0.05 [-0.13, 0.04] | -0.03 [-0.12, 0.05] | -0.03 [-0.11, 0.06] | -0.03 [-0.12, 0.05] |
| *Open sea* | **0.23 *** [0.13, 0.33]** | **0.20 *** [0.10, 0.29]** | **0.13 ** [0.05, 0.22]** | 0.07 [-0.02, 0.15] | 0.08 [-0.00, 0.17] | 0.07 [-0.01, 0.16] |
| *Waterfall or rapids* | **-0.14 * [-0.27, -0.02]** | -0.09 [-0.21, 0.03] | **-0.14 * [-0.24, -0.03]** | **-0.12 * [-0.22, -0.01]** | -0.09 [-0.20, 0.01] | -0.08 [-0.18, 0.03] |
| *Ice rink* | **-0.21 ** [-0.34, -0.08]** | **-0.13 * [-0.25, -0.01]** | 0.01 [-0.10, 0.12] | -0.02 [-0.13, 0.09] | -0.03 [-0.13, 0.08] | 0.06 [-0.05, 0.18] |
| *Rocky or stony shore* | **0.36 *** [0.23, 0.49]** | **0.31 *** [0.19, 0.43]** | **0.20 *** [0.09, 0.31]** | **0.15 ** [0.05, 0.26]** | **0.15 ** [0.04, 0.26]** | **0.13 * [0.02, 0.24]** |
| *Fen, marsh or bog* | **-0.30 *** [-0.45, -0.16]** | **-0.23 *** [-0.37, -0.10]** | **-0.22 *** [-0.35, -0.10]** | **-0.24 *** [-0.36, -0.12]** | **-0.21 *** [-0.33, -0.09]** | **-0.20 ** [-0.33, -0.08]** |
| *Sea cliffs* | **0.31 *** [0.15, 0.48]** | **0.25 *** [0.10, 0.41]** | **0.18 ** [0.05, 0.32]** | **0.15 * [0.01, 0.28]** | **0.15 * [0.02, 0.28]** | **0.14 * [0.01, 0.28]** |
| *Salt marsh, estuary or lagoon* | 0.23 [-0.01, 0.47] | 0.12 [-0.10, 0.34] | 0.04 [-0.16, 0.25] | 0.01 [-0.19, 0.21] | 0.00 [-0.19, 0.20] | 0.01 [-0.19, 0.21] |
| *Urban river/canal* |  |  |  |  |  |  |
| **Visitor characteristics** |  |  |  |  |  |  |
| Perceived financial strain | |  |  |  |  |  |
| *Finding it very difficult* | | 0.01 [-0.07, 0.09] | 0.04 [-0.03, 0.11] | 0.04 [-0.03, 0.11] | 0.03 [-0.03, 0.10] | 0.04 [-0.03, 0.11] |
| *Finding it difficult* |  | 0.03 [-0.02, 0.07] | **0.05 * [0.00, 0.09]** | 0.04 [-0.00, 0.08] | 0.04 [-0.00, 0.08] | 0.04 [-0.00, 0.08] |
| *Coping (ref)* |  |  |  |  |  |  |
| *Living comfortably* |  | -0.02 [-0.06, 0.02] | **-0.05 * [-0.08, -0.01]** | **-0.05 * [-0.08, -0.01]** | **-0.05 ** [-0.08, -0.01]** | **-0.05 ** [-0.08, -0.01]** |
| *Missing* |  | **-0.45 *** [-0.60, -0.30]** | **-0.28 *** [-0.41, -0.14]** | **-0.27 *** [-0.41, -0.14]** | **-0.25 *** [-0.39, -0.11]** | **-0.24 *** [-0.37, -0.10]** |
| Age |  |  |  |  |  |  |
| *60+* |  | **0.21 *** [0.14, 0.28]** | **0.13 *** [0.07, 0.19]** | **0.14 *** [0.08, 0.20]** | **0.13 *** [0.07, 0.19]** | **0.13 *** [0.07, 0.19]** |
| *50-59* |  | **0.24 *** [0.19, 0.30]** | **0.15 *** [0.09, 0.20]** | **0.15 *** [0.09, 0.20]** | **0.14 *** [0.08, 0.19]** | **0.13 *** [0.08, 0.19]** |
| *40-49* |  | **0.23 *** [0.17, 0.29]** | **0.14 *** [0.08, 0.19]** | **0.14 *** [0.09, 0.19]** | **0.13 *** [0.08, 0.18]** | **0.13 *** [0.08, 0.18]** |
| *30-39* |  | **0.07 * [0.01, 0.13]** | 0.03 [-0.02, 0.08] | 0.04 [-0.01, 0.09] | 0.03 [-0.02, 0.08] | 0.03 [-0.02, 0.08] |
| *18 – 29 (ref)* |  |  |  |  |  |  |
| Gender |  |  |  |  |  |  |
| *Male* |  | **-0.16 *** [-0.19, -0.13]** | **-0.13 *** [-0.16, -0.11]** | **-0.14 *** [-0.17, -0.11]** | **-0.14 *** [-0.17, -0.11]** | **-0.13 *** [-0.16, -0.10]** |
| *Female (ref)* |  |  |  |  |  |  |
| Limiting illness/disability | |  |  |  |  |  |
| *Yes a lot* |  | **-0.09 ** [-0.15, -0.02]** | **-0.19 *** [-0.25, -0.13]** | **-0.20 *** [-0.26, -0.14]** | **-0.17 *** [-0.23, -0.12]** | **-0.16 *** [-0.21, -0.10]** |
| *Yes to some extent* |  | -0.04 [-0.08, 0.00] | **-0.04 * [-0.08, -0.01]** | **-0.05 ** [-0.08, -0.01]** | **-0.04 * [-0.07, -0.00]** | -0.03 [-0.07, 0.00] |
| *No (ref)* |  |  |  |  |  |  |
| Garden access |  |  |  |  |  |  |
| *Private garden* |  | 0.05 [-0.00, 0.09] | -0.02 [-0.06, 0.03] | -0.01 [-0.05, 0.03] | -0.01 [-0.06, 0.03] | -0.01 [-0.06, 0.03] |
| *Private outdoor space* |  | 0.03 [-0.03, 0.08] | -0.01 [-0.06, 0.04] | -0.00 [-0.05, 0.04] | -0.00 [-0.05, 0.04] | -0.00 [-0.05, 0.04] |
| *Communal Garden* |  | -0.02 [-0.08, 0.04] | **-0.09 ** [-0.14, -0.03]** | **-0.09 *** [-0.15, -0.04]** | **-0.08 ** [-0.14, -0.03]** | **-0.08 ** [-0.13, -0.03]** |
| *No access (ref)* |  |  |  |  |  |  |
| Employment status |  |  |  |  |  |  |
| *Employed (ref)* |  |  |  |  |  |  |
| *In education* |  | -0.03 [-0.10, 0.05] | 0.00 [-0.06, 0.07] | 0.02 [-0.04, 0.09] | 0.02 [-0.04, 0.09] | 0.02 [-0.05, 0.08] |
| *Housework* |  | 0.03 [-0.04, 0.11] | 0.03 [-0.04, 0.09] | 0.03 [-0.04, 0.09] | 0.02 [-0.05, 0.09] | 0.01 [-0.06, 0.08] |
| *Retired* |  | -0.02 [-0.08, 0.04] | -0.01 [-0.07, 0.04] | -0.01 [-0.07, 0.04] | -0.02 [-0.07, 0.03] | -0.02 [-0.07, 0.04] |
| *Disabled* |  | **0.27 *** [0.17, 0.38]** | **0.26 *** [0.17, 0.35]** | **0.26 *** [0.17, 0.35]** | **0.24 *** [0.15, 0.33]** | **0.24 *** [0.15, 0.33]** |
| *Unemployed* |  | -0.02 [-0.09, 0.05] | -0.01 [-0.08, 0.05] | -0.01 [-0.07, 0.05] | -0.01 [-0.07, 0.05] | -0.01 [-0.07, 0.05] |
| *Other/unsure* |  | **-0.11 * [-0.19, -0.02]** | **-0.09 * [-0.16, -0.01]** | **-0.09 * [-0.17, -0.02]** | **-0.09 * [-0.17, -0.02]** | **-0.08 * [-0.16, -0.01]** |
| Educational attainment |  |  |  |  |  |  |
| *Primary* |  | **-0.18 *** [-0.25, -0.11]** | **-0.13 *** [-0.19, -0.07]** | **-0.14 *** [-0.20, -0.08]** | **-0.13 *** [-0.19, -0.07]** | **-0.12 *** [-0.18, -0.06]** |
| *Secondary* |  | -0.00 [-0.04, 0.03] | 0.01 [-0.02, 0.04] | 0.01 [-0.03, 0.04] | 0.01 [-0.02, 0.04] | 0.01 [-0.02, 0.04] |
| *University (ref)* |  |  |  |  |  |  |
| Perceived minority ethnic group member | |  |  |  |  |  |
| *Yes* |  | -0.02 [-0.09, 0.04] | -0.01 [-0.07, 0.05] | -0.02 [-0.08, 0.04] | -0.00 [-0.06, 0.05] | 0.01 [-0.05, 0.06] |
| *Do not know* |  | **-0.18 *** [-0.27, -0.09]** | **-0.09 * [-0.17, -0.01]** | **-0.09 * [-0.17, -0.00]** | -0.07 [-0.16, 0.01] | -0.06 [-0.14, 0.02] |
| *No (ref)* |  |  |  |  |  |  |
| Relationship status |  |  |  |  |  |  |
| *Married/cohabiting* |  | **0.04 * [0.01, 0.08]** | **0.04 * [0.01, 0.07]** | 0.03 [-0.00, 0.06] | 0.03 [-0.00, 0.06] | 0.02 [-0.01, 0.06] |
| *Missing* |  | **-0.09 * [-0.18, -0.01]** | -0.07 [-0.14, 0.01] | **-0.07 * [-0.15, -0.00]** | -0.07 [-0.14, 0.00] | -0.07 [-0.14, 0.01] |
| *Single (ref)* |  |  |  |  |  |  |
| Dog owner |  |  |  |  |  |  |
| *Yes* |  | **0.07 *** [0.03, 0.11]** | **0.04 * [0.00, 0.07]** | 0.03 [-0.00, 0.06] | 0.03 [-0.00, 0.06] | 0.01 [-0.03, 0.04] |
| *No (ref)* |  |  |  |  |  |  |
| Survey wave |  |  |  |  |  |  |
| *Mar-18* |  | **-0.07 ** [-0.11, -0.02]** | -0.04 [-0.08, 0.00] | -0.01 [-0.05, 0.03] | -0.01 [-0.05, 0.03] | -0.00 [-0.04, 0.04] |
| *Dec-17* |  | **-0.09 *** [-0.13, -0.04]** | **-0.07 ** [-0.11, -0.02]** | -0.04 [-0.08, 0.00] | -0.04 [-0.08, 0.00] | -0.04 [-0.08, 0.01] |
| *Sep-17* |  | -0.03 [-0.07, 0.02] | -0.02 [-0.06, 0.02] | -0.03 [-0.07, 0.01] | -0.03 [-0.07, 0.01] | -0.03 [-0.07, 0.01] |
| *June-17 (ref)* |  |  |  |  |  |  |
| WHO-5 Wellbeing Index | | **0.02 *** [0.01, 0.02]** | **0.01 *** [0.01, 0.01]** | **0.01 *** [0.01, 0.01]** | **0.01 *** [0.01, 0.01]** | **0.01 *** [0.01, 0.01]** |
| **1b) Perceived bluespace qualities** | |  |  |  |  |  |
| Safety |  |  | **0.29 *** [0.28, 0.31]** | **0.28 *** [0.27, 0.30]** | **0.28 *** [0.26, 0.29]** | **0.27 *** [0.26, 0.29]** |
| Presence of wildlife |  |  | **0.07 *** [0.06, 0.08]** | **0.06 *** [0.05, 0.07]** | **0.06 *** [0.05, 0.07]** | **0.06 *** [0.05, 0.07]** |
| Absence of litter/vandalism | |  | **0.04 *** [0.03, 0.05]** | **0.04 *** [0.03, 0.05]** | **0.04 *** [0.03, 0.05]** | **0.04 *** [0.03, 0.05]** |
| Presence of good facilities | |  | **0.04 *** [0.03, 0.05]** | **0.03 *** [0.02, 0.04]** | **0.03 *** [0.02, 0.04]** | **0.03 *** [0.02, 0.04]** |
| Water quality |  |  |  |  |  |  |
| *Excellent* |  |  | **0.18 *** [0.13, 0.22]** | **0.15 *** [0.10, 0.20]** | **0.16 *** [0.11, 0.20]** | **0.16 *** [0.11, 0.20]** |
| *Good* |  |  | 0.02 [-0.01, 0.06] | 0.01 [-0.02, 0.05] | 0.02 [-0.02, 0.05] | 0.02 [-0.02, 0.05] |
| *Sufficient (ref)* |  |  |  |  |  |  |
| *Poor* |  |  | -0.03 [-0.10, 0.03] | -0.01 [-0.08, 0.05] | -0.02 [-0.09, 0.04] | -0.02 [-0.09, 0.04] |
| **RQ2. Exposure** |  |  |  |  |  |  |
| Visit duration |  |  |  |  |  |  |
| *>=3 hr* |  |  |  | **0.49 *** [0.44, 0.54]** | **0.52 *** [0.47, 0.58]** | **0.51 *** [0.45, 0.57]** |
| *2 - <3 hr* |  |  |  | **0.42 *** [0.37, 0.48]** | **0.45 *** [0.39, 0.50]** | **0.43 *** [0.38, 0.49]** |
| *1.5 - <2 hr* |  |  |  | **0.41 *** [0.34, 0.47]** | **0.42 *** [0.35, 0.48]** | **0.40 *** [0.34, 0.47]** |
| *1 - <1.5 hr* |  |  |  | **0.33 *** [0.29, 0.38]** | **0.34 *** [0.30, 0.39]** | **0.33 *** [0.28, 0.38]** |
| *30 mins - <1 hr* |  |  |  | **0.25 *** [0.20, 0.29]** | **0.25 *** [0.21, 0.30]** | **0.24 *** [0.19, 0.29]** |
| *<30 mins (ref)* |  |  |  |  |  |  |
| **Travel characteristics** |  |  |  |  |  |  |
| Travel duration |  |  |  |  |  |  |
| *>=120 mins* |  |  |  |  | **-0.15 *** [-0.20, -0.10]** | **-0.13 *** [-0.19, -0.08]** |
| *60 - <120 mins* |  |  |  |  | **-0.09 *** [-0.13, -0.04]** | **-0.08 *** [-0.12, -0.03]** |
| *30 - <60 mins* |  |  |  |  | -0.01 [-0.06, 0.03] | -0.01 [-0.06, 0.03] |
| *15 - <30 mins* |  |  |  |  | -0.02 [-0.06, 0.02] | -0.02 [-0.06, 0.02] |
| *<15 mins (ref)* |  |  |  |  |  |  |
| Travel mode |  |  |  |  |  |  |
| *Private vehicle* |  |  |  |  | -0.03 [-0.06, 0.01] | -0.03 [-0.06, 0.01] |
| *Public transport* |  |  |  |  | **-0.08 ** [-0.13, -0.02]** | **-0.08 ** [-0.13, -0.02]** |
| *Other* |  |  |  |  | -0.02 [-0.14, 0.09] | -0.02 [-0.13, 0.09] |
| *On foot/bike (ref)* |  |  |  |  |  |  |
| Travel origin |  |  |  |  |  |  |
| *Place of work* |  |  |  |  | **-0.16 *** [-0.23, -0.08]** | **-0.14 *** [-0.21, -0.07]** |
| *Holiday accommodation* |  |  |  |  | 0.02 [-0.04, 0.07] | 0.01 [-0.04, 0.07] |
| *Elsewhere* |  |  |  |  | -0.04 [-0.10, 0.02] | -0.05 [-0.11, 0.01] |
| *Home (ref)* |  |  |  |  |  |  |
| **RQ3. Experience** |  |  |  |  |  |  |
| Activity |  |  |  |  |  |  |
| *Walking without a dog (ref)* | |  |  |  |  |  |
| *Walking with a dog* |  |  |  |  |  | **0.10 *** [0.04, 0.15]** |
| *Socialising* |  |  |  |  |  | **0.06 * [0.01, 0.12]** |
| *Appreciating nature* |  |  |  |  |  | -0.03 [-0.09, 0.04] |
| *Swimming* |  |  |  |  |  | 0.06 [-0.01, 0.12] |
| *Other* |  |  |  |  |  | **-0.14 *** [-0.20, -0.08]** |
| *Playing with children* |  |  |  |  |  | **0.19 *** [0.12, 0.26]** |
| *Running/nordic walking* | |  |  |  |  | -0.07 [-0.14, 0.00] |
| *Sunbathing/paddling* |  |  |  |  |  | 0.03 [-0.04, 0.10] |
| *Eating or drinking* |  |  |  |  |  | -0.02 [-0.10, 0.05] |
| *Quiet activities* |  |  |  |  |  | 0.06 [-0.02, 0.14] |
| *Winter activities* |  |  |  |  |  | **-0.12 ** [-0.21, -0.04]** |
| *Cycling* |  |  |  |  |  | -0.05 [-0.14, 0.03] |
| *Watersports/boating* |  |  |  |  |  | 0.04 [-0.05, 0.13] |
| *Visiting an attraction* |  |  |  |  |  | 0.01 [-0.09, 0.10] |
| *Fishing* |  |  |  |  |  | -0.06 [-0.17, 0.06] |
| *Sport* |  |  |  |  |  | -0.13 [-0.26, 0.01] |
| *Conservation* |  |  |  |  |  | 0.07 [-0.18, 0.33] |
| Visit companions |  |  |  |  |  |  |
| *Adults and children* |  |  |  |  |  | -0.03 [-0.08, 0.01] |
| *Other children only* |  |  |  |  |  | -0.01 [-0.07, 0.06] |
| *Other adults only* |  |  |  |  |  | **0.05 ** [0.02, 0.09]** |
| *Alone (ref)* |  |  |  |  |  |  |
|  |  |  |  |  |  |  |
| N | 14983 | 14955 | 14935 | 14929 | 14918 | 14891 |
| *R^2^* | 0.04 |  |  |  |  |  |
| *Pseudo-R^2^* |  | 0.18 | 0.34 | 0.36 | 0.36 | 0.36 |
| AIC | 44886.26 | 42667.24 | 39532.42 | 39142.29 | 39105.92 | 39066.91 |
| *** *p* < 0.001; ** *p* < 0.01; * *p* < 0.05. | | | | | | |

**Table S7** Linear modelling results for visit satisfaction and each modelling step.

| Satisfied | | | | | | |
| --- | --- | --- | --- | --- | --- | --- |
| names | Bluespace type | Visitor characteristics | Bluespace qualities | Exposure | Travel | Experience |
|  | Coefficient [95% confidence interval] | | | | | |
| Intercept | **1.90 *** [1.87, 1.92]** | **0.92 *** [0.81, 1.03]** | **0.92 *** [0.83, 1.02]** | **0.71 *** [0.61, 0.80]** | **0.77 *** [0.68, 0.87]** | **0.74 *** [0.64, 0.84]** |
| **RQ1 Natural and environmental features** | |  |  |  |  |  |
| 1a) Bluespace type |  |  |  |  |  |  |
| *Seaside promenade* | **0.07 ** [0.02, 0.11]** | **0.08 *** [0.04, 0.13]** | **0.09 *** [0.05, 0.13]** | **0.09 *** [0.05, 0.12]** | **0.08 *** [0.04, 0.11]** | **0.07 *** [0.03, 0.10]** |
| *Lake/reservoir* | **0.12 *** [0.08, 0.17]** | **0.09 *** [0.04, 0.13]** | 0.03 [-0.01, 0.07] | 0.03 [-0.01, 0.07] | 0.02 [-0.02, 0.06] | 0.01 [-0.03, 0.05] |
| *Sandy beach or dunes* | **0.36 *** [0.30, 0.41]** | **0.25 *** [0.20, 0.30]** | **0.16 *** [0.12, 0.21]** | **0.12 *** [0.07, 0.16]** | **0.11 *** [0.07, 0.16]** | **0.09 *** [0.05, 0.14]** |
| *Small water bodies* | **-0.11 *** [-0.16, -0.05]** | **-0.13 *** [-0.18, -0.08]** | **-0.08 *** [-0.12, -0.03]** | -0.02 [-0.06, 0.03] | -0.02 [-0.07, 0.02] | -0.03 [-0.08, 0.02] |
| *Rural river/canal* | **0.07 * [0.01, 0.12]** | 0.02 [-0.03, 0.08] | 0.05 [-0.00, 0.09] | **0.07 ** [0.03, 0.12]** | **0.07 ** [0.02, 0.11]** | **0.07 ** [0.02, 0.11]** |
| *Harbour or marina* | **0.08 * [0.01, 0.15]** | 0.03 [-0.03, 0.10] | 0.05 [-0.01, 0.10] | 0.03 [-0.02, 0.09] | 0.03 [-0.02, 0.09] | 0.02 [-0.03, 0.08] |
| *Water feature/fountain* | **-0.37 *** [-0.44, -0.29]** | **-0.27 *** [-0.34, -0.21]** | **-0.16 *** [-0.22, -0.10]** | -0.04 [-0.10, 0.02] | -0.05 [-0.11, 0.02] | -0.05 [-0.11, 0.01] |
| *Outdoor pool/spa* | **-0.09 * [-0.17, -0.01]** | -0.04 [-0.12, 0.03] | -0.07 [-0.13, 0.00] | **-0.11 *** [-0.18, -0.05]** | **-0.12 *** [-0.19, -0.05]** | **-0.12 *** [-0.19, -0.05]** |
| *Pier* | **-0.18 *** [-0.27, -0.08]** | -0.04 [-0.12, 0.05] | 0.00 [-0.07, 0.08] | 0.01 [-0.06, 0.09] | 0.02 [-0.06, 0.09] | 0.01 [-0.07, 0.09] |
| *Open sea* | **0.19 *** [0.09, 0.28]** | **0.17 *** [0.08, 0.26]** | **0.11 ** [0.03, 0.19]** | 0.05 [-0.03, 0.12] | 0.06 [-0.02, 0.13] | 0.05 [-0.03, 0.13] |
| *Waterfall or rapids* | **-0.20 *** [-0.32, -0.09]** | **-0.15 ** [-0.26, -0.04]** | **-0.20 *** [-0.29, -0.10]** | **-0.18 *** [-0.28, -0.08]** | **-0.16 ** [-0.25, -0.06]** | **-0.14 ** [-0.23, -0.04]** |
| *Ice rink* | **-0.17 ** [-0.29, -0.05]** | **-0.14 * [-0.25, -0.02]** | -0.01 [-0.11, 0.09] | -0.03 [-0.13, 0.07] | -0.04 [-0.14, 0.06] | 0.02 [-0.09, 0.12] |
| *Rocky or stony shore* | **0.34 *** [0.22, 0.46]** | **0.29 *** [0.18, 0.40]** | **0.18 *** [0.09, 0.28]** | **0.15 ** [0.05, 0.24]** | **0.14 ** [0.05, 0.24]** | **0.13 ** [0.03, 0.23]** |
| *Fen, marsh or bog* | **-0.30 *** [-0.43, -0.16]** | **-0.23 *** [-0.36, -0.11]** | **-0.22 *** [-0.33, -0.10]** | **-0.23 *** [-0.34, -0.12]** | **-0.20 *** [-0.31, -0.09]** | **-0.19 *** [-0.30, -0.08]** |
| *Sea cliffs* | **0.33 *** [0.18, 0.48]** | **0.27 *** [0.13, 0.41]** | **0.19 ** [0.07, 0.31]** | **0.16 ** [0.04, 0.28]** | **0.17 ** [0.05, 0.29]** | **0.16 ** [0.04, 0.28]** |
| *Salt marsh, estuary or lagoon* | 0.12 [-0.11, 0.34] | 0.00 [-0.21, 0.21] | -0.06 [-0.25, 0.12] | -0.09 [-0.27, 0.09] | -0.10 [-0.28, 0.09] | -0.09 [-0.27, 0.09] |
| *Urban river/canal* |  |  |  |  |  |  |
| **Visitor characteristics** |  |  |  |  |  |  |
| Perceived financial strain |  |  |  |  |  |  |
| *Finding it very difficult* |  | 0.02 [-0.05, 0.09] | 0.05 [-0.01, 0.11] | 0.05 [-0.01, 0.11] | 0.05 [-0.02, 0.11] | 0.05 [-0.01, 0.11] |
| *Finding it difficult* |  | 0.02 [-0.03, 0.06] | **0.04 * [0.00, 0.08]** | 0.03 [-0.01, 0.07] | 0.03 [-0.01, 0.07] | 0.03 [-0.00, 0.07] |
| *Coping (ref)* |  |  |  |  |  |  |
| *Living comfortably* |  | -0.01 [-0.05, 0.03] | **-0.03 * [-0.07, -0.00]** | **-0.03 * [-0.07, -0.00]** | **-0.04 * [-0.07, -0.00]** | **-0.04 * [-0.07, -0.00]** |
| *Missing* |  | **-0.35 *** [-0.49, -0.21]** | **-0.17 ** [-0.29, -0.04]** | **-0.17 ** [-0.29, -0.04]** | **-0.14 * [-0.27, -0.02]** | **-0.13 * [-0.25, -0.01]** |
| Age |  |  |  |  |  |  |
| *60+* |  | **0.17 *** [0.11, 0.23]** | **0.09 ** [0.03, 0.14]** | **0.10 *** [0.04, 0.15]** | **0.08 ** [0.03, 0.14]** | **0.08 ** [0.02, 0.13]** |
| *50-59* |  | **0.19 *** [0.14, 0.25]** | **0.09 *** [0.04, 0.14]** | **0.09 *** [0.04, 0.14]** | **0.08 *** [0.03, 0.13]** | **0.07 ** [0.03, 0.12]** |
| *40-49* |  | **0.17 *** [0.12, 0.22]** | **0.08 ** [0.03, 0.12]** | **0.08 ** [0.03, 0.12]** | **0.07 ** [0.02, 0.12]** | **0.07 ** [0.03, 0.12]** |
| *30-39* |  | 0.03 [-0.02, 0.09] | -0.00 [-0.05, 0.04] | -0.00 [-0.05, 0.04] | -0.01 [-0.05, 0.04] | -0.00 [-0.05, 0.04] |
| *18 – 29 (ref)* |  |  |  |  |  |  |
| Gender |  |  |  |  |  |  |
| *Male* |  | **-0.16 *** [-0.19, -0.13]** | **-0.14 *** [-0.16, -0.11]** | **-0.14 *** [-0.16, -0.11]** | **-0.14 *** [-0.16, -0.11]** | **-0.13 *** [-0.16, -0.10]** |
| *Female (ref)* |  |  |  |  |  |  |
| Limiting illness/disability |  |  |  |  |  |  |
| *Yes a lot* |  | **-0.09 ** [-0.15, -0.04]** | **-0.20 *** [-0.25, -0.14]** | **-0.20 *** [-0.26, -0.15]** | **-0.18 *** [-0.23, -0.13]** | **-0.16 *** [-0.21, -0.11]** |
| *Yes to some extent* |  | **-0.07 *** [-0.11, -0.04]** | **-0.08 *** [-0.11, -0.05]** | **-0.08 *** [-0.11, -0.05]** | **-0.07 *** [-0.10, -0.04]** | **-0.07 *** [-0.10, -0.03]** |
| *No (ref)* |  |  |  |  |  |  |
| Garden access |  |  |  |  |  |  |
| *Private garden* |  | **0.07 ** [0.03, 0.12]** | 0.01 [-0.03, 0.05] | 0.01 [-0.03, 0.05] | 0.01 [-0.03, 0.05] | 0.01 [-0.02, 0.05] |
| *Private outdoor space* |  | **0.05 * [0.00, 0.10]** | 0.01 [-0.03, 0.06] | 0.02 [-0.02, 0.06] | 0.02 [-0.03, 0.06] | 0.02 [-0.02, 0.06] |
| *Communal Garden* |  | -0.01 [-0.06, 0.05] | **-0.07 ** [-0.12, -0.02]** | **-0.07 ** [-0.12, -0.02]** | **-0.06 * [-0.11, -0.01]** | **-0.05 * [-0.10, -0.01]** |
| *No access (ref)* |  |  |  |  |  |  |
| Employment status |  |  |  |  |  |  |
| *Employed (ref)* |  |  |  |  |  |  |
| *In education* |  | -0.04 [-0.11, 0.03] | -0.01 [-0.07, 0.05] | 0.01 [-0.05, 0.07] | 0.00 [-0.05, 0.06] | -0.00 [-0.06, 0.05] |
| *Housework* |  | 0.01 [-0.06, 0.09] | 0.00 [-0.06, 0.07] | 0.00 [-0.06, 0.07] | -0.00 [-0.06, 0.06] | -0.01 [-0.07, 0.06] |
| *Retired* |  | -0.01 [-0.07, 0.04] | -0.00 [-0.05, 0.05] | -0.00 [-0.05, 0.05] | -0.01 [-0.05, 0.04] | -0.00 [-0.05, 0.04] |
| *Disabled* |  | **0.27 *** [0.17, 0.36]** | **0.25 *** [0.17, 0.34]** | **0.25 *** [0.17, 0.34]** | **0.23 *** [0.15, 0.32]** | **0.23 *** [0.14, 0.31]** |
| *Unemployed* |  | -0.04 [-0.10, 0.03] | -0.03 [-0.09, 0.03] | -0.03 [-0.09, 0.03] | -0.03 [-0.09, 0.03] | -0.03 [-0.08, 0.03] |
| *Other/unsure* |  | **-0.13 ** [-0.21, -0.05]** | **-0.11 ** [-0.18, -0.04]** | **-0.11 ** [-0.18, -0.04]** | **-0.11 ** [-0.18, -0.04]** | **-0.10 ** [-0.17, -0.04]** |
| Educational attainment |  |  |  |  |  |  |
| *Primary* |  | **-0.14 *** [-0.20, -0.08]** | **-0.09 ** [-0.14, -0.03]** | **-0.10 *** [-0.15, -0.04]** | **-0.08 ** [-0.14, -0.03]** | **-0.08 ** [-0.13, -0.02]** |
| *Secondary* |  | 0.01 [-0.02, 0.04] | 0.02 [-0.00, 0.05] | 0.02 [-0.01, 0.05] | 0.02 [-0.01, 0.05] | 0.02 [-0.01, 0.05] |
| *University (ref)* |  |  |  |  |  |  |
| Perceived minority ethnic group member | |  |  |  |  |  |
| *Yes* |  | -0.00 [-0.06, 0.06] | 0.01 [-0.04, 0.07] | 0.00 [-0.05, 0.05] | 0.02 [-0.03, 0.07] | 0.03 [-0.02, 0.08] |
| *Do not know* |  | **-0.17 *** [-0.26, -0.09]** | **-0.09 * [-0.16, -0.01]** | **-0.08 * [-0.16, -0.01]** | -0.07 [-0.14, 0.00] | -0.06 [-0.13, 0.01] |
| *No (ref)* |  |  |  |  |  |  |
| Relationship status |  |  |  |  |  |  |
| *Married/cohabiting* |  | **0.04 ** [0.01, 0.08]** | **0.04 ** [0.01, 0.07]** | **0.03 * [0.00, 0.06]** | **0.03 * [0.00, 0.06]** | **0.03 * [0.00, 0.06]** |
| *Missing* |  | -0.07 [-0.15, 0.00] | -0.04 [-0.11, 0.02] | -0.05 [-0.12, 0.02] | -0.04 [-0.11, 0.02] | -0.04 [-0.11, 0.02] |
| *Single (ref)* |  |  |  |  |  |  |
| Dog owner |  |  |  |  |  |  |
| *Yes* |  | **0.08 *** [0.04, 0.11]** | **0.05 ** [0.02, 0.08]** | **0.04 * [0.01, 0.07]** | **0.04 * [0.01, 0.07]** | 0.02 [-0.01, 0.06] |
| *No (ref)* |  |  |  |  |  |  |
| Survey wave |  |  |  |  |  |  |
| *Mar-18* |  | -0.04 [-0.08, 0.00] | -0.01 [-0.05, 0.02] | 0.01 [-0.02, 0.05] | 0.01 [-0.02, 0.05] | 0.02 [-0.02, 0.05] |
| *Dec-17* |  | **-0.07 ** [-0.11, -0.03]** | **-0.05 ** [-0.09, -0.01]** | -0.03 [-0.06, 0.01] | -0.03 [-0.06, 0.01] | -0.02 [-0.06, 0.01] |
| *Sep-17* |  | -0.02 [-0.06, 0.02] | -0.02 [-0.05, 0.02] | -0.02 [-0.06, 0.02] | -0.02 [-0.06, 0.02] | -0.02 [-0.06, 0.02] |
| *June-17 (ref)* |  |  |  |  |  |  |
| WHO-5 Wellbeing Index |  | **0.02 *** [0.01, 0.02]** | **0.01 *** [0.01, 0.01]** | **0.01 *** [0.01, 0.01]** | **0.01 *** [0.01, 0.01]** | **0.01 *** [0.01, 0.01]** |
| **1b) Perceived bluespace qualities** | |  |  |  |  |  |
| Safety |  |  | **0.29 *** [0.28, 0.30]** | **0.28 *** [0.27, 0.29]** | **0.28 *** [0.27, 0.29]** | **0.27 *** [0.26, 0.29]** |
| Presence of wildlife |  |  | **0.05 *** [0.04, 0.06]** | **0.05 *** [0.04, 0.05]** | **0.05 *** [0.04, 0.05]** | **0.05 *** [0.04, 0.05]** |
| Absence of litter/vandalism |  |  | **0.05 *** [0.04, 0.06]** | **0.05 *** [0.04, 0.06]** | **0.05 *** [0.04, 0.06]** | **0.05 *** [0.04, 0.06]** |
| Presence of good facilities |  |  | **0.04 *** [0.03, 0.05]** | **0.03 *** [0.02, 0.04]** | **0.03 *** [0.02, 0.04]** | **0.03 *** [0.02, 0.04]** |
| Water quality |  |  |  |  |  |  |
| *Excellent* |  |  | **0.21 *** [0.17, 0.26]** | **0.19 *** [0.15, 0.23]** | **0.20 *** [0.15, 0.24]** | **0.20 *** [0.15, 0.24]** |
| *Good* |  |  | **0.04 * [0.00, 0.07]** | 0.03 [-0.01, 0.06] | 0.03 [-0.00, 0.07] | 0.03 [-0.00, 0.07] |
| *Sufficient (ref)* |  |  |  |  |  |  |
| *Poor* |  |  | **-0.10 *** [-0.16, -0.04]** | **-0.09 ** [-0.14, -0.03]** | **-0.10 *** [-0.15, -0.04]** | **-0.10 *** [-0.15, -0.04]** |
| **RQ2. Exposure** |  |  |  |  |  |  |
| Visit duration |  |  |  |  |  |  |
| *>=3 hr* |  |  |  | **0.43 *** [0.38, 0.47]** | **0.46 *** [0.41, 0.51]** | **0.45 *** [0.39, 0.50]** |
| *2 - <3 hr* |  |  |  | **0.35 *** [0.30, 0.39]** | **0.37 *** [0.32, 0.41]** | **0.36 *** [0.31, 0.41]** |
| *1.5 - <2 hr* |  |  |  | **0.31 *** [0.26, 0.37]** | **0.32 *** [0.26, 0.38]** | **0.31 *** [0.26, 0.37]** |
| *1 - <1.5 hr* |  |  |  | **0.28 *** [0.24, 0.33]** | **0.29 *** [0.25, 0.34]** | **0.28 *** [0.24, 0.33]** |
| *30 mins - <1 hr* |  |  |  | **0.20 *** [0.16, 0.24]** | **0.20 *** [0.16, 0.25]** | **0.20 *** [0.15, 0.24]** |
| *<30 mins (ref)* |  |  |  |  |  |  |
| **Travel characteristics** |  |  |  |  |  |  |
| Travel duration |  |  |  |  |  |  |
| *>=120 mins* |  |  |  |  | **-0.15 *** [-0.20, -0.10]** | **-0.13 *** [-0.18, -0.08]** |
| *60 - <120 mins* |  |  |  |  | **-0.08 *** [-0.12, -0.04]** | **-0.07 ** [-0.11, -0.03]** |
| *30 - <60 mins* |  |  |  |  | -0.03 [-0.07, 0.01] | -0.03 [-0.07, 0.01] |
| *15 - <30 mins* |  |  |  |  | -0.01 [-0.05, 0.03] | -0.01 [-0.05, 0.03] |
| *<15 mins (ref)* |  |  |  |  |  |  |
| Travel mode |  |  |  |  |  |  |
| *Private vehicle* |  |  |  |  | -0.02 [-0.06, 0.01] | -0.03 [-0.06, 0.00] |
| *Public transport* |  |  |  |  | -0.03 [-0.09, 0.02] | -0.03 [-0.09, 0.02] |
| *Other* |  |  |  |  | -0.02 [-0.12, 0.09] | -0.01 [-0.11, 0.09] |
| *On foot/bike (ref)* |  |  |  |  |  |  |
| Travel origin |  |  |  |  |  |  |
| *Place of work* |  |  |  |  | **-0.16 *** [-0.23, -0.10]** | **-0.15 *** [-0.21, -0.08]** |
| *Holiday accommodation* |  |  |  |  | 0.01 [-0.04, 0.07] | 0.01 [-0.04, 0.06] |
| *Elsewhere* |  |  |  |  | **-0.06 * [-0.11, -0.00]** | **-0.06 * [-0.12, -0.01]** |
| *Home (ref)* |  |  |  |  |  |  |
| **RQ3. Experience** |  |  |  |  |  |  |
| Activity |  |  |  |  |  |  |
| *Walking without a dog (ref)* |  |  |  |  |  |  |
| *Walking with a dog* |  |  |  |  |  | **0.09 *** [0.05, 0.14]** |
| *Socialising* |  |  |  |  |  | **0.08 ** [0.03, 0.14]** |
| *Appreciating nature* |  |  |  |  |  | 0.02 [-0.04, 0.08] |
| *Swimming* |  |  |  |  |  | 0.04 [-0.02, 0.10] |
| *Other* |  |  |  |  |  | **-0.11 *** [-0.17, -0.06]** |
| *Playing with children* |  |  |  |  |  | **0.19 *** [0.13, 0.25]** |
| *Running/nordic walking* |  |  |  |  |  | **-0.11 ** [-0.17, -0.04]** |
| *Sunbathing/paddling* |  |  |  |  |  | 0.04 [-0.03, 0.10] |
| *Eating or drinking* |  |  |  |  |  | 0.03 [-0.04, 0.09] |
| *Quiet activities* |  |  |  |  |  | 0.06 [-0.01, 0.13] |
| *Winter activities* |  |  |  |  |  | -0.04 [-0.11, 0.04] |
| *Cycling* |  |  |  |  |  | -0.06 [-0.14, 0.01] |
| *Watersports/boating* |  |  |  |  |  | 0.05 [-0.03, 0.13] |
| *Visiting an attraction* |  |  |  |  |  | 0.05 [-0.04, 0.13] |
| *Fishing* |  |  |  |  |  | -0.05 [-0.15, 0.06] |
| *Sport* |  |  |  |  |  | **-0.22 *** [-0.34, -0.10]** |
| *Conservation* |  |  |  |  |  | -0.10 [-0.33, 0.13] |
| Visit companions |  |  |  |  |  |  |
| *Adults and children* |  |  |  |  |  | **-0.05 * [-0.09, -0.01]** |
| *Other children only* |  |  |  |  |  | -0.04 [-0.10, 0.02] |
| *Other adults only* |  |  |  |  |  | **0.04 ** [0.01, 0.08]** |
| *Alone (ref)* |  |  |  |  |  |  |
|  |  |  |  |  |  |  |
| N | 14984 | 14956 | 14935 | 14929 | 14918 | 14892 |
| *R^2^* | 0.04 |  |  |  |  |  |
| *Pseudo-R^2^* |  | 0.19 | 0.37 | 0.38 | 0.39 | 0.39 |
| AIC | 42686.77 | 40350.77 | 36599.13 | 36262.34 | 36219.04 | 36170.75 |
| *** *p* < 0.001; ** *p* < 0.01; * *p* < 0.05. | | | | | | |

**Table S8.**

Fully adjusted linear model for each wellbeing outcome of visit happiness, anxious, worthwhile and satisfaction including an interaction term between perceived presence of wildlife and blue space type.

| names | Happy | Anxious | Worthwhile | Satisfied |
| --- | --- | --- | --- | --- |
|  | Coefficient [95% confidence interval] | | | |
| Intercept | **0.51 *** [0.40, 0.63]** | **-1.50 *** [-1.68, -1.31]** | **0.60 *** [0.49, 0.72]** | **0.71 *** [0.61, 0.81]** |
| **RQ1 Natural and environmental features** |  |  |  |  |
| 1a) Bluespace type |  |  |  |  |
| *Seaside promenade* | **0.10 *** [0.05, 0.14]** | -0.04 [-0.11, 0.02] | **0.10 *** [0.05, 0.14]** | **0.09 *** [0.05, 0.13]** |
| *Lake/reservoir* | 0.04 [-0.01, 0.09] | -0.02 [-0.10, 0.05] | 0.01 [-0.04, 0.06] | 0.03 [-0.01, 0.08] |
| *Sandy beach or dunes* | **0.17 *** [0.11, 0.22]** | **-0.12 ** [-0.20, -0.04]** | **0.16 *** [0.11, 0.22]** | **0.13 *** [0.08, 0.18]** |
| *Small water bodies* | -0.04 [-0.10, 0.02] | -0.03 [-0.12, 0.06] | -0.06 [-0.12, 0.00] | -0.04 [-0.09, 0.01] |
| *Rural river/canal* | 0.02 [-0.04, 0.08] | -0.06 [-0.15, 0.03] | 0.01 [-0.05, 0.07] | **0.07 * [0.01, 0.12]** |
| *Harbour or marina* | -0.00 [-0.07, 0.06] | 0.01 [-0.09, 0.10] | 0.05 [-0.02, 0.11] | 0.05 [-0.01, 0.11] |
| *Water feature/fountain* | -0.02 [-0.09, 0.05] | -0.05 [-0.15, 0.05] | -0.05 [-0.12, 0.02] | -0.05 [-0.11, 0.02] |
| *Outdoor pool/spa* | **-0.09 * [-0.17, -0.01]** | **0.21 *** [0.09, 0.33]** | **-0.09 * [-0.17, -0.01]** | **-0.11 ** [-0.18, -0.03]** |
| *Pier* | 0.02 [-0.06, 0.11] | 0.10 [-0.03, 0.22] | 0.01 [-0.08, 0.09] | 0.03 [-0.05, 0.11] |
| *Open sea* | **0.10 * [0.01, 0.20]** | 0.03 [-0.11, 0.17] | 0.07 [-0.03, 0.17] | 0.06 [-0.03, 0.15] |
| *Waterfall or rapids* | **-0.20 *** [-0.32, -0.09]** | 0.12 [-0.05, 0.29] | -0.10 [-0.22, 0.02] | **-0.15 ** [-0.25, -0.04]** |
| *Ice rink* | 0.03 [-0.09, 0.14] | -0.02 [-0.19, 0.15] | 0.05 [-0.06, 0.17] | -0.01 [-0.11, 0.10] |
| *Rocky or stony shore* | **0.25 *** [0.13, 0.38]** | **-0.22 * [-0.40, -0.04]** | **0.20 ** [0.08, 0.33]** | **0.20 *** [0.09, 0.31]** |
| *Fen, marsh or bog* | **-0.43 *** [-0.57, -0.28]** | 0.08 [-0.14, 0.29] | **-0.38 *** [-0.53, -0.23]** | **-0.34 *** [-0.48, -0.21]** |
| *Sea cliffs* | **0.15 * [0.00, 0.30]** | -0.18 [-0.40, 0.04] | **0.19 * [0.04, 0.34]** | **0.19 ** [0.05, 0.32]** |
| *Salt marsh, estuary or lagoon* | -0.14 [-0.41, 0.14] | 0.14 [-0.27, 0.54] | -0.16 [-0.44, 0.11] | -0.17 [-0.43, 0.08] |
| *Urban river/canal* |  |  |  |  |
| **1b) Perceived bluespace qualities** |  |  |  |  |
| Presence of wildlife v_wildlife_numeric | **0.09 *** [0.07, 0.10]** | **0.07 *** [0.05, 0.09]** | **0.08 *** [0.06, 0.09]** | **0.05 *** [0.04, 0.07]** |
| Safety | **0.28 *** [0.27, 0.29]** | **-0.20 *** [-0.22, -0.18]** | **0.27 *** [0.26, 0.29]** | **0.27 *** [0.26, 0.28]** |
| Absence of litter/vandalism | **0.04 *** [0.02, 0.05]** | -0.02 [-0.03, 0.00] | **0.04 *** [0.03, 0.05]** | **0.05 *** [0.04, 0.06]** |
| Presence of good facilities | **0.02 *** [0.01, 0.03]** | **0.03 *** [0.02, 0.05]** | **0.03 *** [0.02, 0.04]** | **0.03 *** [0.02, 0.04]** |
| Water quality |  |  |  |  |
| *Excellent* | **0.20 *** [0.15, 0.25]** | **-0.10 ** [-0.17, -0.04]** | **0.16 *** [0.11, 0.20]** | **0.20 *** [0.15, 0.24]** |
| *Good* | **0.05 * [0.01, 0.08]** | -0.03 [-0.09, 0.02] | 0.02 [-0.02, 0.05] | 0.03 [-0.00, 0.07] |
| *Sufficient (ref)* |  |  |  |  |
| *Poor* | -0.00 [-0.07, 0.06] | -0.02 [-0.11, 0.07] | -0.02 [-0.09, 0.04] | **-0.10 *** [-0.15, -0.04]** |
| **RQ2. Exposure** |  |  |  |  |
| Visit duration |  |  |  |  |
| *>=3 hr* | **0.44 *** [0.39, 0.50]** | **-0.24 *** [-0.32, -0.15]** | **0.50 *** [0.44, 0.56]** | **0.44 *** [0.39, 0.49]** |
| *2 - <3 hr* | **0.38 *** [0.33, 0.44]** | **-0.14 *** [-0.22, -0.06]** | **0.43 *** [0.38, 0.48]** | **0.35 *** [0.31, 0.40]** |
| *1.5 - <2 hr* | **0.37 *** [0.30, 0.43]** | **-0.15 ** [-0.25, -0.06]** | **0.40 *** [0.34, 0.46]** | **0.31 *** [0.25, 0.37]** |
| *1 - <1.5 hr* | **0.30 *** [0.25, 0.35]** | -0.07 [-0.14, 0.00] | **0.33 *** [0.28, 0.38]** | **0.28 *** [0.23, 0.32]** |
| *30 mins - <1 hr* | **0.21 *** [0.16, 0.25]** | 0.02 [-0.04, 0.09] | **0.24 *** [0.19, 0.28]** | **0.19 *** [0.15, 0.23]** |
| *<30 mins (ref)* |  |  |  |  |
| **Travel characteristics** |  |  |  |  |
| Travel duration |  |  |  |  |
| *>=120 mins* | **-0.12 *** [-0.17, -0.07]** | **0.36 *** [0.28, 0.43]** | **-0.13 *** [-0.18, -0.08]** | **-0.13 *** [-0.17, -0.08]** |
| *60 - <120 mins* | -0.03 [-0.07, 0.02] | **0.32 *** [0.25, 0.38]** | **-0.07 ** [-0.12, -0.03]** | **-0.06 ** [-0.11, -0.02]** |
| *30 - <60 mins* | 0.04 [-0.00, 0.09] | **0.12 *** [0.05, 0.18]** | -0.01 [-0.05, 0.04] | -0.03 [-0.07, 0.01] |
| *15 - <30 mins* | 0.03 [-0.01, 0.07] | 0.00 [-0.06, 0.06] | -0.02 [-0.06, 0.03] | -0.01 [-0.05, 0.03] |
| *<15 mins (ref)* |  |  |  |  |
| Travel mode |  |  |  |  |
| *Private vehicle* | -0.01 [-0.05, 0.02] | **0.07 ** [0.02, 0.12]** | -0.03 [-0.06, 0.01] | -0.03 [-0.06, 0.00] |
| *Public transport* | **-0.09 ** [-0.14, -0.03]** | **0.15 *** [0.06, 0.23]** | **-0.07 * [-0.13, -0.02]** | -0.03 [-0.08, 0.02] |
| *Other* | 0.03 [-0.08, 0.14] | -0.01 [-0.17, 0.15] | -0.02 [-0.13, 0.09] | -0.01 [-0.11, 0.09] |
| *On foot/bike (ref)* |  |  |  |  |
| Travel origin |  |  |  |  |
| *Place of work* | **-0.22 *** [-0.29, -0.15]** | **0.11 * [0.01, 0.22]** | **-0.13 *** [-0.20, -0.06]** | **-0.14 *** [-0.20, -0.07]** |
| *Holiday accommodation* | -0.01 [-0.07, 0.05] | -0.05 [-0.13, 0.04] | 0.01 [-0.04, 0.07] | 0.01 [-0.04, 0.06] |
| *Elsewhere* | **-0.07 * [-0.13, -0.01]** | 0.01 [-0.08, 0.10] | -0.05 [-0.11, 0.01] | **-0.07 * [-0.12, -0.01]** |
| *Home (ref)* |  |  |  |  |
| **RQ3. Experience** |  |  |  |  |
| Activity |  |  |  |  |
| *Walking without a dog (ref)* |  |  |  |  |
| *Walking with a dog* | **0.10 *** [0.05, 0.15]** | -0.05 [-0.12, 0.03] | **0.10 *** [0.04, 0.15]** | **0.09 *** [0.04, 0.14]** |
| *Socialising* | **0.12 *** [0.06, 0.17]** | **-0.13 ** [-0.22, -0.05]** | **0.06 * [0.00, 0.12]** | **0.08 ** [0.03, 0.13]** |
| *Appreciating nature* | 0.05 [-0.01, 0.12] | **-0.21 *** [-0.30, -0.12]** | -0.03 [-0.09, 0.03] | 0.02 [-0.04, 0.07] |
| *Swimming* | 0.04 [-0.03, 0.10] | -0.08 [-0.17, 0.02] | 0.05 [-0.02, 0.12] | 0.04 [-0.02, 0.10] |
| *Other* | **-0.12 *** [-0.19, -0.06]** | 0.04 [-0.05, 0.13] | **-0.14 *** [-0.20, -0.08]** | **-0.11 *** [-0.16, -0.05]** |
| *Playing with children* | **0.23 *** [0.17, 0.30]** | **-0.21 *** [-0.31, -0.11]** | **0.19 *** [0.12, 0.25]** | **0.19 *** [0.13, 0.25]** |
| *Running/nordic walking* | **-0.08 * [-0.15, -0.01]** | **0.30 *** [0.20, 0.40]** | -0.07 [-0.14, 0.01] | **-0.10 ** [-0.17, -0.04]** |
| *Sunbathing/paddling* | **0.11 ** [0.04, 0.18]** | **-0.21 *** [-0.31, -0.10]** | 0.03 [-0.05, 0.10] | 0.03 [-0.03, 0.10] |
| *Eating or drinking* | 0.04 [-0.03, 0.11] | **-0.18 *** [-0.28, -0.07]** | -0.02 [-0.10, 0.05] | 0.03 [-0.04, 0.09] |
| *Quiet activities* | **0.09 * [0.01, 0.17]** | **-0.27 *** [-0.38, -0.15]** | 0.06 [-0.02, 0.14] | 0.06 [-0.01, 0.13] |
| *Winter activities* | **-0.14 ** [-0.22, -0.05]** | **0.28 *** [0.15, 0.41]** | **-0.14 ** [-0.23, -0.05]** | -0.05 [-0.13, 0.03] |
| *Cycling* | -0.02 [-0.11, 0.06] | 0.02 [-0.10, 0.14] | -0.06 [-0.14, 0.03] | -0.07 [-0.14, 0.01] |
| *Watersports/boating* | 0.00 [-0.09, 0.09] | 0.05 [-0.07, 0.18] | 0.04 [-0.05, 0.13] | 0.06 [-0.02, 0.14] |
| *Visiting an attraction* | 0.04 [-0.05, 0.13] | -0.10 [-0.23, 0.04] | 0.00 [-0.09, 0.10] | 0.05 [-0.04, 0.13] |
| *Fishing* | 0.10 [-0.01, 0.22] | -0.00 [-0.17, 0.16] | -0.06 [-0.18, 0.05] | -0.05 [-0.16, 0.05] |
| *Sport* | **-0.20 ** [-0.33, -0.07]** | **0.31 ** [0.11, 0.50]** | -0.12 [-0.25, 0.01] | **-0.21 *** [-0.33, -0.09]** |
| *Conservation* | **-0.38 ** [-0.63, -0.13]** | **0.61 ** [0.24, 0.98]** | 0.10 [-0.15, 0.35] | -0.07 [-0.30, 0.16] |
| Visit companions |  |  |  |  |
| *Adults and children* | **-0.05 * [-0.10, -0.00]** | **0.23 *** [0.16, 0.29]** | -0.03 [-0.08, 0.01] | **-0.05 * [-0.09, -0.01]** |
| *Other children only* | -0.01 [-0.08, 0.05] | **0.14 ** [0.04, 0.23]** | -0.01 [-0.07, 0.06] | -0.04 [-0.10, 0.01] |
| *Other adults only* | **0.04 * [0.00, 0.07]** | -0.02 [-0.07, 0.04] | **0.06 ** [0.02, 0.09]** | **0.05 ** [0.01, 0.08]** |
| *Alone (ref)* |  |  |  |  |
| **Visitor characteristics** |  |  |  |  |
| Perceived financial strain |  |  |  |  |
| *Finding it very difficult* | **0.12 *** [0.05, 0.18]** | -0.02 [-0.12, 0.08] | 0.04 [-0.03, 0.11] | 0.05 [-0.01, 0.11] |
| *Finding it difficult* | **0.05 * [0.01, 0.09]** | 0.03 [-0.03, 0.09] | 0.04 [-0.00, 0.08] | 0.03 [-0.00, 0.07] |
| *Coping (ref)* |  |  |  |  |
| *Living comfortably* | **-0.06 *** [-0.10, -0.03]** | 0.02 [-0.03, 0.07] | **-0.05 * [-0.08, -0.01]** | **-0.03 * [-0.07, -0.00]** |
| *Missing* | **-0.23 *** [-0.37, -0.10]** | **0.21 * [0.01, 0.40]** | **-0.24 *** [-0.37, -0.10]** | **-0.13 * [-0.25, -0.01]** |
| Age |  |  |  |  |
| *60+* | 0.02 [-0.04, 0.08] | **-0.49 *** [-0.58, -0.40]** | **0.13 *** [0.07, 0.19]** | **0.08 ** [0.02, 0.13]** |
| *50-59* | 0.03 [-0.02, 0.08] | **-0.50 *** [-0.57, -0.42]** | **0.13 *** [0.08, 0.19]** | **0.07 ** [0.03, 0.12]** |
| *40-49* | 0.05 [-0.00, 0.10] | **-0.38 *** [-0.45, -0.30]** | **0.13 *** [0.08, 0.18]** | **0.07 ** [0.03, 0.12]** |
| *30-39* | 0.00 [-0.05, 0.05] | **-0.16 *** [-0.23, -0.09]** | 0.03 [-0.02, 0.08] | -0.00 [-0.05, 0.04] |
| *18 – 29 (ref)* |  |  |  |  |
| Gender |  |  |  |  |
| *Male* | **-0.17 *** [-0.20, -0.14]** | **0.11 *** [0.07, 0.16]** | **-0.13 *** [-0.16, -0.10]** | **-0.13 *** [-0.16, -0.10]** |
| *Female (ref)* |  |  |  |  |
| Limiting illness/disability |  |  |  |  |
| *Yes a lot* | **-0.17 *** [-0.22, -0.11]** | **1.02 *** [0.94, 1.10]** | **-0.15 *** [-0.21, -0.10]** | **-0.16 *** [-0.21, -0.11]** |
| *Yes to some extent* | **-0.04 * [-0.08, -0.01]** | **0.27 *** [0.22, 0.32]** | -0.03 [-0.06, 0.01] | **-0.06 *** [-0.09, -0.03]** |
| *No (ref)* |  |  |  |  |
| Garden access |  |  |  |  |
| *Private garden* | 0.02 [-0.02, 0.07] | -0.02 [-0.08, 0.04] | -0.01 [-0.06, 0.03] | 0.01 [-0.03, 0.05] |
| *Private outdoor space* | 0.03 [-0.02, 0.07] | **-0.07 * [-0.14, -0.00]** | -0.00 [-0.05, 0.04] | 0.02 [-0.02, 0.06] |
| *Communal Garden* | **-0.06 * [-0.12, -0.01]** | **0.20 *** [0.12, 0.27]** | **-0.08 ** [-0.13, -0.02]** | **-0.05 * [-0.10, -0.00]** |
| *No access (ref)* |  |  |  |  |
| Employment status |  |  |  |  |
| *Employed (ref)* |  |  |  |  |
| *In education* | -0.00 [-0.07, 0.06] | **-0.12 * [-0.21, -0.03]** | 0.02 [-0.05, 0.08] | -0.00 [-0.06, 0.05] |
| *Housework* | 0.02 [-0.05, 0.09] | 0.03 [-0.07, 0.13] | 0.01 [-0.06, 0.08] | -0.01 [-0.07, 0.06] |
| *Retired* | -0.05 [-0.10, 0.01] | -0.08 [-0.15, 0.00] | -0.02 [-0.07, 0.04] | -0.00 [-0.05, 0.04] |
| *Disabled* | **0.13 ** [0.04, 0.22]** | **-0.40 *** [-0.53, -0.26]** | **0.23 *** [0.14, 0.33]** | **0.22 *** [0.14, 0.31]** |
| *Unemployed* | -0.06 [-0.12, 0.00] | **-0.10 * [-0.19, -0.01]** | -0.01 [-0.07, 0.06] | -0.03 [-0.08, 0.03] |
| *Other/unsure* | -0.01 [-0.09, 0.06] | -0.09 [-0.20, 0.02] | **-0.08 * [-0.16, -0.01]** | **-0.10 ** [-0.17, -0.04]** |
| Educational attainment |  |  |  |  |
| *Primary* | **-0.13 *** [-0.19, -0.08]** | 0.08 [-0.00, 0.17] | **-0.12 *** [-0.18, -0.06]** | **-0.08 ** [-0.13, -0.02]** |
| *Secondary* | 0.00 [-0.03, 0.04] | 0.02 [-0.02, 0.07] | 0.01 [-0.02, 0.04] | 0.02 [-0.01, 0.05] |
| *University (ref)* |  |  |  |  |
| Perceived minority ethnic group member |  |  |  |  |
| *Yes* | -0.04 [-0.09, 0.02] | **0.32 *** [0.24, 0.41]** | 0.01 [-0.05, 0.06] | 0.03 [-0.02, 0.08] |
| *Do not know* | **-0.14 *** [-0.22, -0.06]** | **0.39 *** [0.27, 0.51]** | -0.06 [-0.14, 0.02] | -0.06 [-0.13, 0.02] |
| *No (ref)* |  |  |  |  |
| Relationship status |  |  |  |  |
| *Married/cohabiting* | 0.03 [-0.00, 0.06] | 0.04 [-0.01, 0.08] | 0.03 [-0.01, 0.06] | **0.03 * [0.00, 0.06]** |
| *Missing* | -0.01 [-0.08, 0.06] | 0.06 [-0.05, 0.16] | -0.06 [-0.14, 0.01] | -0.04 [-0.10, 0.03] |
| *Single (ref)* |  |  |  |  |
| Dog owner |  |  |  |  |
| *Yes* | -0.00 [-0.04, 0.03] | 0.01 [-0.04, 0.06] | 0.01 [-0.03, 0.05] | 0.02 [-0.01, 0.06] |
| *No (ref)* |  |  |  |  |
| Survey wave |  |  |  |  |
| *Mar-18* | 0.00 [-0.04, 0.04] | 0.02 [-0.04, 0.08] | -0.00 [-0.04, 0.04] | 0.02 [-0.02, 0.06] |
| *Dec-17* | -0.02 [-0.06, 0.02] | 0.05 [-0.01, 0.10] | -0.03 [-0.07, 0.01] | -0.02 [-0.06, 0.02] |
| *Sep-17* | -0.02 [-0.06, 0.02] | 0.01 [-0.05, 0.07] | -0.03 [-0.07, 0.01] | -0.02 [-0.06, 0.02] |
| *June-17 (ref)* |  |  |  |  |
| WHO-5 Wellbeing Index | **0.01 *** [0.01, 0.01]** | **-0.00 * [-0.00, -0.00]** | **0.01 *** [0.01, 0.01]** | **0.01 *** [0.01, 0.01]** |
| **Interaction – bluespace type x presence of wildlife** | |  |  |  |
| *Seaside promenade x wildlife* | **-0.04 *** [-0.06, -0.02]** | -0.02 [-0.05, 0.02] | **-0.03 ** [-0.05, -0.01]** | **-0.02 * [-0.05, -0.00]** |
| *Lake/reservoir x wildlife* | -0.02 [-0.04, 0.01] | -0.02 [-0.06, 0.02] | -0.00 [-0.03, 0.02] | -0.00 [-0.03, 0.02] |
| *Sandy beach or dunes x wildlife* | **-0.05 *** [-0.08, -0.03]** | -0.03 [-0.06, 0.01] | **-0.04 ** [-0.07, -0.01]** | -0.02 [-0.05, 0.00] |
| *Small water bodies x wildlife* | 0.03 [-0.00, 0.06] | **-0.05 * [-0.09, -0.00]** | 0.02 [-0.01, 0.05] | **0.03 * [0.00, 0.05]** |
| *Rural river/canal x wildlife* | 0.03 [-0.00, 0.06] | -0.03 [-0.07, 0.01] | 0.03 [-0.00, 0.05] | 0.02 [-0.01, 0.04] |
| *Harbour or marina x wildlife* | -0.00 [-0.04, 0.03] | -0.04 [-0.09, 0.01] | -0.03 [-0.06, 0.01] | -0.02 [-0.06, 0.01] |
| *Water feature/fountain x wildlife* | **-0.07 *** [-0.10, -0.03]** | 0.03 [-0.02, 0.08] | **-0.06 *** [-0.09, -0.02]** | **-0.03 * [-0.06, -0.00]** |
| *Outdoor pool/spa x wildlife* | -0.04 [-0.07, 0.00] | 0.04 [-0.02, 0.09] | **-0.06 ** [-0.09, -0.02]** | **-0.04 * [-0.07, -0.00]** |
| *Pier x wildlife* | -0.01 [-0.05, 0.04] | 0.00 [-0.06, 0.07] | -0.04 [-0.08, 0.01] | -0.01 [-0.05, 0.03] |
| *Open sea x wildlife* | 0.00 [-0.05, 0.05] | -0.02 [-0.09, 0.05] | 0.03 [-0.02, 0.08] | 0.01 [-0.04, 0.05] |
| *Waterfall or rapids x wildlife* | **0.08 ** [0.02, 0.14]** | **0.10 * [0.01, 0.19]** | 0.05 [-0.01, 0.11] | 0.03 [-0.03, 0.08] |
| *Ice rink x wildlife* | **-0.10 *** [-0.16, -0.05]** | -0.02 [-0.10, 0.06] | **-0.10 *** [-0.16, -0.05]** | **-0.10 *** [-0.15, -0.05]** |
| *Rocky or stony shore x wildlife* | **-0.11 *** [-0.17, -0.05]** | 0.03 [-0.05, 0.12] | -0.05 [-0.11, 0.01] | -0.05 [-0.11, 0.00] |
| *Fen, marsh or bog x wildlife* | **0.23 *** [0.16, 0.30]** | **0.12 * [0.01, 0.22]** | **0.16 *** [0.09, 0.24]** | **0.15 *** [0.08, 0.21]** |
| *Sea cliffs x wildlife* | -0.00 [-0.08, 0.07] | 0.05 [-0.07, 0.16] | -0.03 [-0.10, 0.05] | -0.01 [-0.08, 0.06] |
| *Salt marsh, estuary or lagoon x wildlife* | 0.07 [-0.07, 0.21] | -0.15 [-0.35, 0.05] | **0.14 * [0.00, 0.28]** | 0.07 [-0.06, 0.20] |
| *Urban river/canal x wildlife (ref)* |  |  |  |  |
|  |  |  |  |  |
| N | 14892 | 14891 | 14891 | 14892 |
| *Pseudo-R^2^* | 0.38 | 0.22 | 0.37 | 0.39 |
| AIC | 38858.55 | 50176.25 | 39116.92 | 36238.75 |
| *** *p* < 0.001; ** *p* < 0.01; * *p* < 0.05. | | | | |

**Reference**

**1.** L. R. Elliott, M. White, P., "BlueHealth International Survey Methodology and Technical Report" (2020)
